# Evolutionary and Topological Properties of Genes and Community Structures in Human Gene Regulatory Networks

**DOI:** 10.1371/journal.pcbi.1005009

**Published:** 2016-06-30

**Authors:** Anthony Szedlak, Nicholas Smith, Li Liu, Giovanni Paternostro, Carlo Piermarocchi

**Affiliations:** 1 Department of Physics and Astronomy, Michigan State University, East Lansing, Michigan, United States of America; 2 Salgomed Inc., Del Mar, California, United States of America; 3 College of Health Solutions, Arizona State University, Tempe, Arizona, United States of America; 4 Sanford Burnham Prebys Medical Discovery Institute, La Jolla, California, United States of America; University of Chicago, UNITED STATES

## Abstract

The diverse, specialized genes present in today’s lifeforms evolved from a common core of ancient, elementary genes. However, these genes did not evolve individually: gene expression is controlled by a complex network of interactions, and alterations in one gene may drive reciprocal changes in its proteins’ binding partners. Like many complex networks, these gene regulatory networks (GRNs) are composed of communities, or clusters of genes with relatively high connectivity. A deep understanding of the relationship between the evolutionary history of single genes and the topological properties of the underlying GRN is integral to evolutionary genetics. Here, we show that the topological properties of an acute myeloid leukemia GRN and a general human GRN are strongly coupled with its genes’ evolutionary properties. Slowly evolving (“cold”), old genes tend to interact with each other, as do rapidly evolving (“hot”), young genes. This naturally causes genes to segregate into community structures with relatively homogeneous evolutionary histories. We argue that gene duplication placed old, cold genes and communities at the center of the networks, and young, hot genes and communities at the periphery. We demonstrate this with single-node centrality measures and two new measures of efficiency, the set efficiency and the interset efficiency. We conclude that these methods for studying the relationships between a GRN’s community structures and its genes’ evolutionary properties provide new perspectives for understanding evolutionary genetics.

## Author Summary

We found strong relationships between the community structures and evolutionary properties of an acute myeloid leukemia gene regulatory network (GRN) and a general human GRN. Interacting genes tend to have similar evolutionary ages and rates, causing the GRNs to segregate into slowly-evolving (“cold”), old gene communities and rapidly-evolving (“hot”), young gene communities. The coldest, oldest communities are centrally located and are highly enriched for gene groups related to fundamental cellular functions, whereas the hottest, youngest communities are peripheral and enriched for gene groups related to higher order functions.

## Introduction

The evolutionary history of a gene can be mapped in various ways. The absolute evolutionary rate, for example, can be computed from observed differences in orthologs across species in the context of their phylogenetic relationships [1], whereas the age of a gene can be measured by tracing when the gene first appeared in the organism’s phylogenetic tree [2]. Quantities such as these allow researchers to chronicle the journey of individual genes across evolutionary history.

But genes do not exist, and therefore do not evolve, in isolation. Mutations in a transcription factor may affect the expression of the genes it regulates, since changes in a protein’s amino acid sequence can cause it to lose compatibility with former binding partners, and gain compatibility with new partners. Accumulation of these alterations can lead to changes in fitness and, eventually, speciation. The evolution of individual genes is thus coupled with the evolution of the structure of the organism’s gene regulatory network (GRN), and network properties should be related to the evolutionary properties of its constituent nodes and edges.

It has been proposed that GRNs grow and evolve incrementally via gene duplication followed by mutation and functional divergence [3–7], although changes may have occasionally arrived in bursts, as in whole-genome duplication [8]. This time-dependent network formation suggests that GRNs are composed of a core of ancient, conserved genes with fundamental functions, and younger, peripheral genes with species- or cell type-specific function, which mutate frequently until the functions of the newly created pathways are optimized. These mutations can alter GRNs by creating, removing, reassigning, or changing other properties of nodes and edges.

Fraser et al. demonstrated that interacting pairs of proteins have similar evolutionary rates [9]. This constraint is likely driven by the necessity of coevolution, since a change in one protein’s sequence may require a corresponding change in its partner’s sequence in order for the pair to remain compatible. Daub et al. showed that genes which are part of many biological pathways have lower evolutionary rates than genes which belong to few or no known pathways, further supporting the idea that related genes share similar evolutionary properties [10]. It has also been shown that evolutionary rates are weakly, but significantly, negatively correlated with degree, closeness centrality, and betweenness centrality (network measures which quantify the location of individual nodes in different ways) [11, 12], and that essential genes have high centrality and low evolutionary rates [13].

Here, our goal is to establish quantitative relationships between the evolutionary history of genes and their topological properties in an acute myeloid leukemia GRN, AML 2.3 [14], as well as for a general human GRN, HumanNet [15]. In contrast to the earlier studies above, we go beyond an analysis based on single-node centrality and pairwise measures by studying the connection between topology and evolution from the point of view of network community structures. We demonstrate that the evolutionary rates and ages of genes are not randomly distributed across the networks, but are naturally organized in communities with well-defined evolutionary characteristics: old genes cluster with old genes, and young cluster with young. Likewise, “cold genes” (genes with low evolutionary rates) cluster with cold genes, and “hot genes” (genes with high evolutionary rates) cluster with hot genes. This segregation also exists for groups of enriched genes identified by DAVID [16] within the communities. In terms of network topology, we show that genes and DAVID groups which are old and cold tend to be central, and those which are young and hot tend to be peripheral. We demonstrate this with traditional single-node centrality measures as well as two new network measures, the set efficiency [14] and the interset efficiency, which quantify the mean distance between all nodes within a single set and between two sets, respectively (see Methods). We find that PageRank [17], a finite-range centrality measure, shows stronger biological significance than degree (a local measure) and betweenness centrality (a global measure), and that the set efficiency and interset efficiency correlate strongly with the evolutionary histories of individual genes and DAVID groups.

## Results and Discussion

While computed differently, a gene’s evolutionary rate (ER) and its age are related. Young genes with novel functions need time to fine-tune their properties in order to optimize the fitness of the host organism, so young genes tend to be hot. Likewise, old genes with fundamental roles, such as protein translation, have had enough time to sufficiently optimize their functions, and so should change very slowly. As expected, the ERs and ages of the genes present in AML 2.3 are strongly correlated (*R* = 0.504, *p* < 10^−300^).

Consistent with previous results [9], interacting genes tend to have similar ERs and ages. S1–S4 Figs show that the distributions of differences in ERs and ages between genes linked by an edge in AML 2.3 are significantly closer to zero than those of degree-preserving randomizations of the same network, with an approximate z-score of 96.8 for differences in ER and 72.0 for differences in age. This tendency for connected genes to have similar ERs and ages hints that there may be large-scale segregation between clusters of old, cold genes and young, hot genes. Indeed, this is reflected in the natural community structure present in AML 2.3, as well as in the DAVID groups present within these communities.

The main results of the community analysis are in Table 1 for AML 2.3 and Table 2 for HumanNet. These tables list the ER and age properties for the ten largest network communities, and for the three most significantly enriched DAVID groups found within each community. The ERs and ages for many of these DAVID groups reflect their biological functions. Zinc finger proteins, which are enriched in both AML 2.3 and HumanNet, are involved in a large number of heterogeneous cellular processes [18], so their genes need to adapt more often than genes with very specific singular functions. They also have a particularly high rate of duplication and loss, so while the family itself is old (found in animals, plants [19], and fungi [20]), individual genes in this family are young [21]. Genes involved in transcriptional regulation must also be flexible enough to tune the expression of target genes in response to environmental changes over time [22, 23]. The olfactory group is enriched in HumanNet, and it is significantly younger than average. A small number of olfactory genes were present in early chordates, but olfactory systems became far more complex and diverse in land-dwelling animals, particularly in mammals [24]. Conversely, the most fundamental DAVID groups have experienced few changes since early single-celled lifeforms. DAVID groups such as mRNA metabolic process [25] and translational elongation [26] in AML 2.3 as well as ribosome [27] and protein kinase core [28] in HumanNet are old and stable, having long ago optimized their functions.

**Table 1:** Evolutionary properties of communities and DAVID groups in AML 2.3. Gene evolutionary rates (ERs) take real values from 0 (most conserved) to approximately 6.9 (most variable), and ages take integer values from 0 (oldest) to 12 (youngest). The table is organized as follows. "Comm. Index" is the index of the ten largest communities. "Num. genes" is the number of genes in the community. "Comm. ER" indicates whether the community is significantly hotter (i.e. has a higher ER) or colder (i.e. has a lower ER) than the mean of 300 equally-sized sets of genes randomly selected from the network, with a significance threshold of *p* = 10^−3^. "Diff. in mean" is the difference between the mean ER of the community and the mean ER of the 300 randomly selected sets. "p-value" is the significance of the difference. "Comm. age", "Diff. in mean", and "p-value" are the same as previously stated, but for age rather than ER. "DAVID group name" is the name of the DAVID group that DAVID identified as enriched in each community. “Group type” states whether the DAVID group is a protein type (P), location of final gene product (L), biological process (B), or cellular component (C). "Num. genes" is the number of genes in the DAVID group. "DAVID Benjamini" is the significance of the enrichment of the DAVID group, as reported by DAVID. The remaining DAVID group columns are computed in the same manner as the community columns.

*Comm*. *index*	*Num*. *genes*	*Comm*. *ER*	*Diff*. *in mean*	*p-value*	*Comm*. *age*	*Diff*. *in mean*	*p-value*	*DAVID group name*	*Group type*	*Num*. *genes*	*DAVID Benjamini*	*Group ER*	*Diff*. *in mean*	*p-value*	*Group age*	*Diff*. *in mean*	*p-value*
**0**	1760	hot	0.24	2.5E-81	young	1.01	6.7E-28	Zinc finger	P	275	1.2E-19	hot	0.32	6.0E-19	young	0.75	4.4E-05
								Transcription regulation	B	298	1.9E-16	hot	0.28	4.8E-14	young	1.17	9.3E-11
**1**	1579	average	-0.03	2.3E-02	old	-0.39	6.8E-07	Transcription	B	304	4.8E-24	average	-0.05	4.6E-02	young	0.69	5.6E-05
								Intracellular organelle lumen	L	239	9.7E-19	average	-0.07	7.1E-02	old	-1.18	1.7E-08
								Protein localization	B	145	2.9E-10	cold	-0.27	7.3E-05	old	-1.50	5.2E-08
**2**	1208	cold	-0.10	2.6E-10	old	-1.30	1.2E-58	Nuclear lumen	L	332	8.4E-107	cold	-0.21	2.6E-07	old	-1.94	1.6E-28
								Cell cycle phase	B	169	3.6E-98	average	0.06	1.0E-01	old	-1.67	5.4E-07
								Response to DNA damage stimulus	B	135	3.5E-58	average	0.03	3.1E-01	old	-1.97	3.2E-07
**3**	1055	average	0.01	2.4E-01	young	1.41	5.4E-53	Cell-cell signaling	B	146	3.6E-39	cold	-0.16	7.0E-04	young	1.12	1.3E-05
								Plasma membrane part	C	359	1.1E-67	cold	-0.11	2.5E-04	young	0.82	4.1E-06
**4**	867	cold	-0.27	3.7E-55	old	-0.94	1.7E-16	mRNA metabolic process	B	124	9.5E-66	cold	-0.46	2.7E-25	old	-1.84	2.7E-06
								Nuclear lumen	L	212	9.3E-60	cold	-0.34	5.3E-24	old	-1.45	1.2E-09
								mRNA transport	B	27	1.2E-11	average	-0.36	5.8E-03	average	-2.17	7.2E-03
**5**	780	cold	-0.11	1.1E-05	old	-0.91	5.1E-10	Establishment of protein localization	B	105	1.1E-19	cold	-0.33	5.2E-09	old	-2.03	1.6E-12
								Actin filament-based process	B	51	1.9E-16	cold	-0.41	9.0E-06	old	-1.96	9.0E-06
								Regulation of programmed cell death	B	79	2.6E-07	average	-0.03	4.2E-01	average	-0.63	3.0E-02
**6**	748	hot	0.18	3.5E-22	young	1.34	2.8E-29	Lymphocyte activation	B	40	1.2E-11	hot	0.68	1.2E-10	young	3.27	6.2E-11
								Hemoglobin complex	P	13	1.3E-12	average	0.54	5.1E-03	young	4.43	1.1E-04
								Transmembrane	P	252	1.8E-05	hot	0.51	2.0E-34	young	2.55	4.5E-28
**7**	417	average	-0.08	1.2E-02	old	-1.54	8.3E-22	Mitochondrial envelope	C	103	1.0E-68	average	-0.14	2.0E-02	old	-1.98	4.5E-10
								Respiratory chain	C	44	4.9E-47	average	0.03	3.4E-01	old	-1.57	9.3E-05
								Ribosomal protein	P	62	2.4E-53	average	0.18	2.3E-02	old	-1.73	3.2E-05
**8**	296	hot	0.16	1.5E-05	young	1.49	4.8E-14	Homeobox	P	27	2.2E-12	average	-0.22	7.3E-02	young	3.01	3.7E-07
**9**	270	cold	-0.28	5.7E-14	old	-1.96	2.9E-20	Translational elongation	B	77	1.7E-114	cold	-0.60	6.0E-18	old	-3.04	1.7E-13
								rRNA processing	B	27	1.1E-22	average	-0.24	3.2E-02	old	-2.93	1.7E-05

**Table 2:** Evolutionary properties of communities and DAVID groups in HumanNet. See Table 1 for explanation of column headers.

*Comm*. *index*	*Num*. *genes*	*Comm*. *ER*	*Diff*. *in mean*	*p-value*	*Comm*. *age*	*Diff*. *in mean*	*p-value*	*DAVID group name*	*Group type*	*Num*. *genes*	*DAVID Benjamini*	*Group ER*	*Diff*. *in mean*	*p-value*	*Group age*	*Diff*. *in mean*	*p-value*
0	2961	hot	0.38	1.0E-185	young	2.05	7.1E-198	Immunoglobulin domain	P	201	7.6E-66	hot	0.92	4.0E-82	young	4.16	2.2E-58
								EGF-like domain	P	122	6.8E-55	hot	0.25	1.5E-04	young	1.02	3.3E-04
								Chemotaxis	B	93	1.5E-40	hot	0.86	4.7E-31	young	4.09	1.9E-23
1	2849	cold	-0.14	8.7E-30	average	0.10	8.2E-03	Protein kinase core	P	344	3.2E-226	cold	-0.30	1.5E-15	old	-1.69	2.0E-18
								Pos. reg. of transcr. from RNAP II promoter	B	181	4.0E-57	cold	-0.30	1.3E-08	young	0.69	4.6E-03
								Transcription regulation	B	503	4.2E-59	cold	-0.22	2.0E-11	young	1.04	1.8E-12
2	2665	cold	-0.12	9.1E-21	old	-1.50	5.3E-97	Mitochondrial envelope	C	211	2.7E-81	cold	-0.16	5.0E-04	old	-2.24	1.0E-23
								Protein transport	B	243	1.2E-51	cold	-0.35	2.6E-15	old	-2.20	1.9E-21
								Peroxisome	C	54	2.6E-30	average	-0.11	1.2E-01	old	-2.39	2.0E-06
3	1402	cold	-0.07	1.7E-05	old	-1.05	2.7E-28	Response to DNA damage stimulus	B	145	1.9E-71	average	0.03	3.3E-01	old	-2.35	2.0E-16
								Cellular macromolecule catabolic process	B	195	1.6E-70	cold	-0.31	5.4E-10	old	-2.10	7.2E-16
								Mitosis	B	82	1.2E-58	average	0.01	3.8E-01	old	-1.83	1.9E-06
4	1115	cold	-0.24	7.1E-30	old	-2.20	8.3E-89	Ribosome	C	68	7.2E-89	cold	-0.64	9.6E-16	old	-3.50	1.8E-15
								Nuclear lumen	L	241	3.6E-59	cold	-0.29	5.6E-13	old	-2.38	5.6E-27
								Nucleosome	C	61	2.5E-29	cold	-0.50	9.6E-06	average	-0.09	4.0E-01
5	781	cold	-0.26	1.6E-24	old	-1.08	8.1E-16	mRNA processing	B	151	1.4E-144	cold	-0.51	9.8E-25	old	-2.32	3.5E-15
								Nuclear lumen	L	185	1.1E-75	cold	-0.39	1.9E-15	old	-2.15	1.1E-15
								WD40 repeat region	P	83	5.1E-60	cold	-0.30	5.3E-05	old	-1.95	9.3E-07
6	736	cold	-0.09	6.1E-04	young	0.75	1.3E-07	Sulfotransferase	P	26	1.6E-33	average	0.06	3.4E-01	average	0.23	3.6E-01
								Vision	B	32	1.2E-16	average	0.03	3.9E-01	average	0.84	1.1E-01
								C2 calcium-dependent membrane targeting	P	25	6.7E-11	average	-0.14	1.7E-01	average	0.14	4.5E-01
7	731	hot	0.20	9.7E-16	average	0.17	1.1E-01	KRAB	P	168	9.5E-208	hot	0.67	4.9E-36	average	0.66	1.1E-02
								Zinc finger	P	205	3.4E-245	hot	0.62	1.4E-32	average	0.84	1.3E-03
								Domain: SCAN box	P	22	1.4E-20	hot	0.54	3.2E-04	young	4.82	8.1E-10
8	320	average	0.05	1.2E-01	young	0.70	4.6E-04	ANK repeat	P	58	2.4E-72	average	0.07	2.4E-01	average	0.15	3.9E-01
								Kelch repeat	P	41	1.2E-65	cold	-0.43	1.0E-04	average	0.89	5.1E-02
								Domain: SOCS box	P	16	6.3E-21	average	-0.35	2.6E-02	average	2.68	2.2E-03
9	130	average	0.19	4.7E-03	young	2.30	4.8E-12	Olfaction	B	37	4.9E-38	average	0.71	1.4E-03	young	7.17	1.0E-31
								Cell membrane	C	54	1.2E-22	average	0.24	4.4E-02	young	5.09	1.4E-29
								MORN	P	7	1.4E-09	average	-0.03	4.2E-01	average	-2.50	2.6E-02

As a control for the enriched DAVID groups, ten new communities were built by randomly shuffling the genes between communities from the network, while maintaining the size of each community. The resulting random communities were then analyzed using DAVID. This randomization procedure was followed for both AML 2.3 and HumanNet, and in both cases, the enrichment was far less significant than for the real communities. The enriched DAVID groups in Tables 1 and 2 are thus biologically meaningful, not merely coincidental. See S2 Table for the comparison between the real and control DAVID groups.

The same analysis from Tables 1 and 2 was conducted for a normal hematopoietic stem cell network (see S1A Table and Methods). This normal network is of lower quality than AML 2.3 because it was constructed from a much smaller data set; however, it serves as a qualitative control and further validates the results of our analysis. S1B Table compares the tables from S1A Table between the AML and normal networks. Several of the same DAVID groups are enriched in both networks, and each has several enriched blood-specific DAVID groups (lymphocyte activation, and hemoglobin complex for AML; regulation of leukocyte activation, platelet alpha granule, and complement and coagulation cascades for normal). The lower quality of the normal network is evident in the p-values, as the findings for both AML 2.3 and HumanNet are more significant.

**Fig 1:**
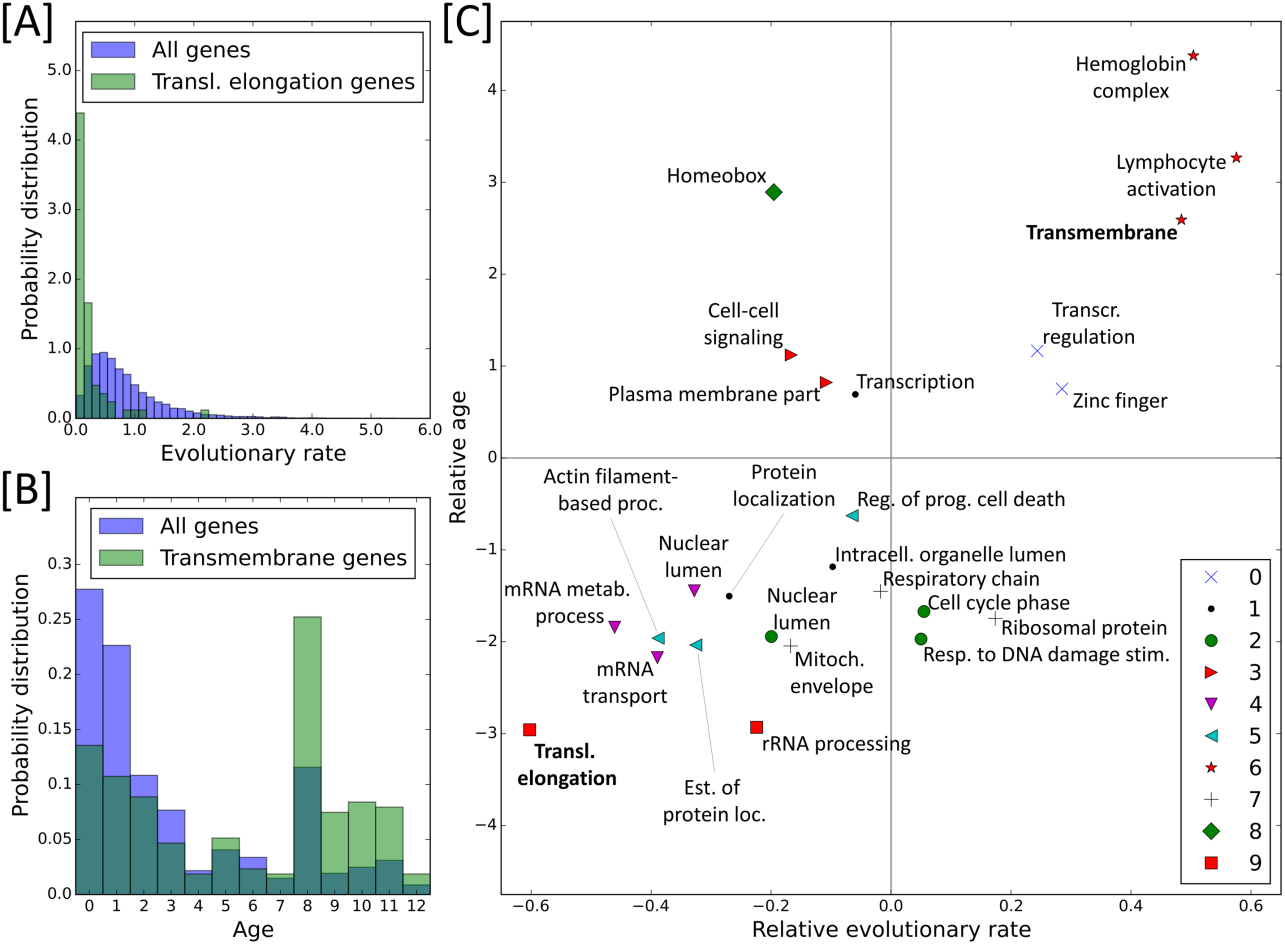
Ages and evolutionary rates for enriched DAVID groups in AML 2.3. (A) Distribution of evolutionary rates (ERs), measured in units of the number of nonsynonymous substitutions per amino acid site per billion years, for all genes (purple) and for genes in the translational elongation DAVID group (green). This DAVID group has a very low ER compared to the background distribution. (B) Distribution of ages for all genes (purple) and genes in the transmembrane DAVID group (green), where age = 0 is the oldest and age = 12 is the youngest. Transmembrane genes are much younger than average. (C) Summary of mean ER and mean age for DAVID groups in Table 1. The relative ERs on the x-axis are computed from *ER*_*relative*_ = *ER*_*func*. *group mean*_ − *ER*_*network mean*_, and likewise for relative age on the y-axis. The DAVID groups from (A) and (B) have bold labels in (C). Each marker type corresponds to one of communities 0 through 9. As expected, old DAVID groups tend to have a low average ER (i.e. are “cold”), and young DAVID groups tend to evolve frequently (i.e. are “hot”). Unabbreviated DAVID group names are listed in Table 1.

Fig 1 analyzes DAVID groups in AML 2.3 and their relation with network communities and evolutionary properties. Fig 1A shows that the ER distribution of translational elongation genes is noticeably left-shifted relative to the ERs of all genes, indicating that it hosts relatively slowly evolving genes. Transmembrane genes are much younger than average, as shown in Fig 1B. Fig 1C provides a comprehensive picture of the evolutionary properties of the ten largest network communities (symbols) with their main DAVID groups (as labeled).

**Fig 2:**
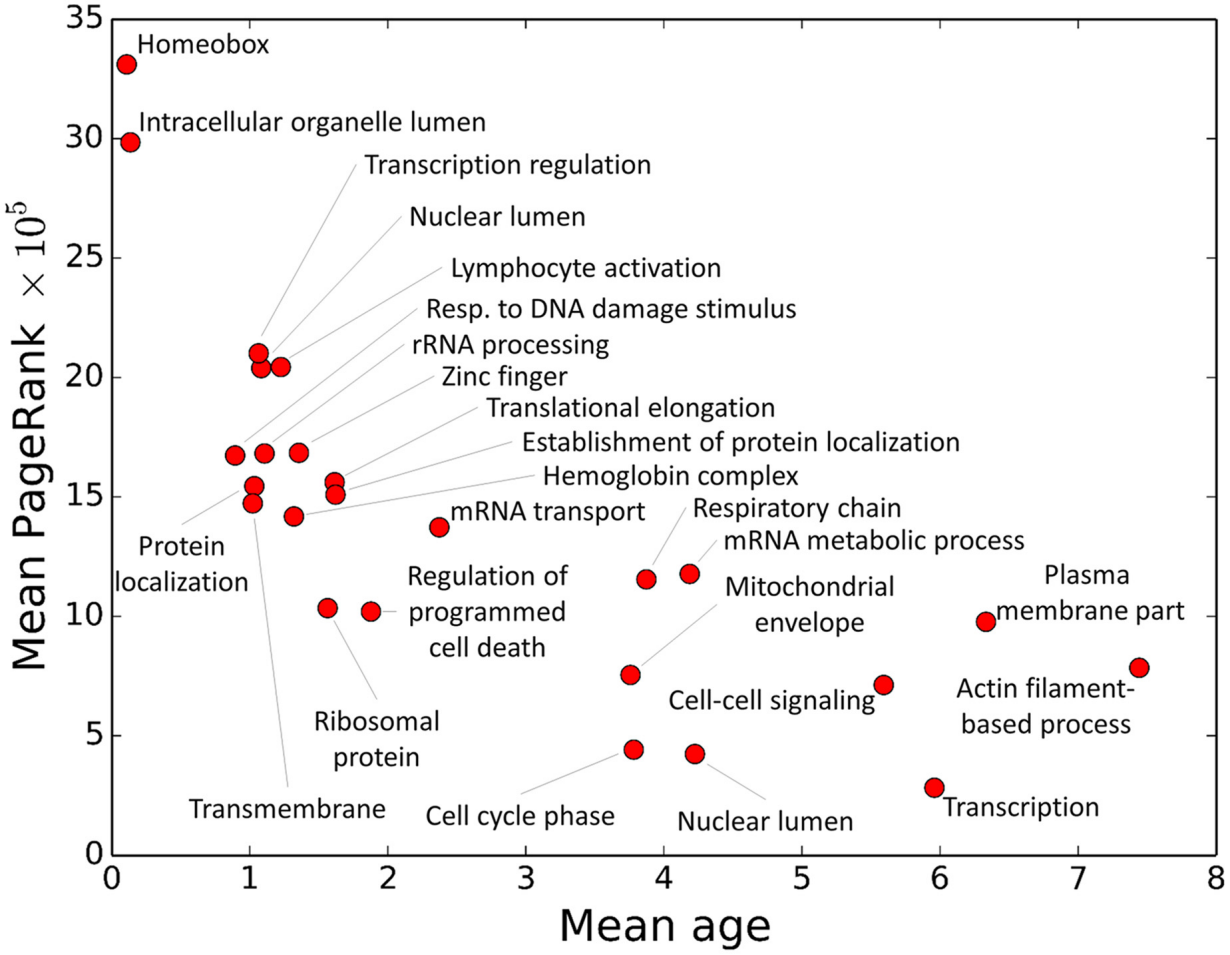
PageRank and DAVID groups for AML 2.3. Mean PageRank versus mean age of each DAVID group from Table 1 (age = 0 is the oldest and age = 12 is the youngest). Old DAVID groups tend to have high PageRank. Unabbreviated DAVID group names are listed in Table 1.

Dividing genes into DAVID groups causes stronger relationships between network topology and evolutionary properties to emerge. Traditional single-node centrality measures such as degree, betweenness centrality [29], and PageRank [17] show small but significant correlation with ERs and ages, with the oldest, coldest genes being the most central (see Table 3). Grouping genes by DAVID group leads to stronger correlations, the clearest of which is between the mean PageRank and mean age, shown in Fig 2 (Pearson’s *R* = −0.75, *p* = 1 × 10^−5^; Spearman’s *ρ* = −0.86, *p* = 5 × 10^−8^; see S1 File for all scatter plots). These three centrality measures are related, but differ in their global reach. Degree is completely local, only dependent on the number of neighbors of a gene; betweenness centrality is global, requiring information from the entire network; but PageRank is between these extremes, influenced by all genes but with more weight granted to those genes which are near-by. The strong correlation between PageRank and evolutionary measures thus may be explained by the presence of communities in the GRN, since community structure itself is strongly correlated with ER and age, as shown in Tables 1 and 2.

**Table 3:** Centrality and evolutionary measures in AML 2.3. Single-node centrality measures exhibit a small but significant correlation with evolutionary rate and age. The DAVID groups’ average centrality measures show stronger correlation with evolutionary properties, particularly between PageRank and age.

	*Degree centrality*				*PageRank*				*Betweenness centrality*			
	*Pearson R*	*p-value*	*Spearman ρ*	*p-value*	*Pearson R*	*p-value*	*Spearman ρ*	*p-value*	*Pearson R*	*p-value*	*Spearman ρ*	*p-value*
***Single gene evol*. *rate***	-0.06	8.9E-10	-0.15	7.4E-51	-0.14	1.5E-43	-0.25	5.6E-141	-0.07	1.2E-11	-0.21	3.0E-103
***Single gene age***	-0.06	7.3E-09	-0.18	2.5E-73	-0.12	4.1E-33	-0.22	1.7E-111	-0.04	5.5E-05	-0.14	2.0E-47
***DAVID group evol*. *rate***	-0.13	5.3E-01	-0.04	8.4E-01	-0.58	2.3E-03	-0.57	2.8E-03	-0.25	2.3E-01	-0.18	3.9E-01
***DAVID group age***	-0.1	6.4E-01	-0.21	3.1E-01	-0.75	1.4E-05	-0.86	5.1E-08	-0.26	2.1E-01	-0.23	2.7E-01

**Fig 3:**
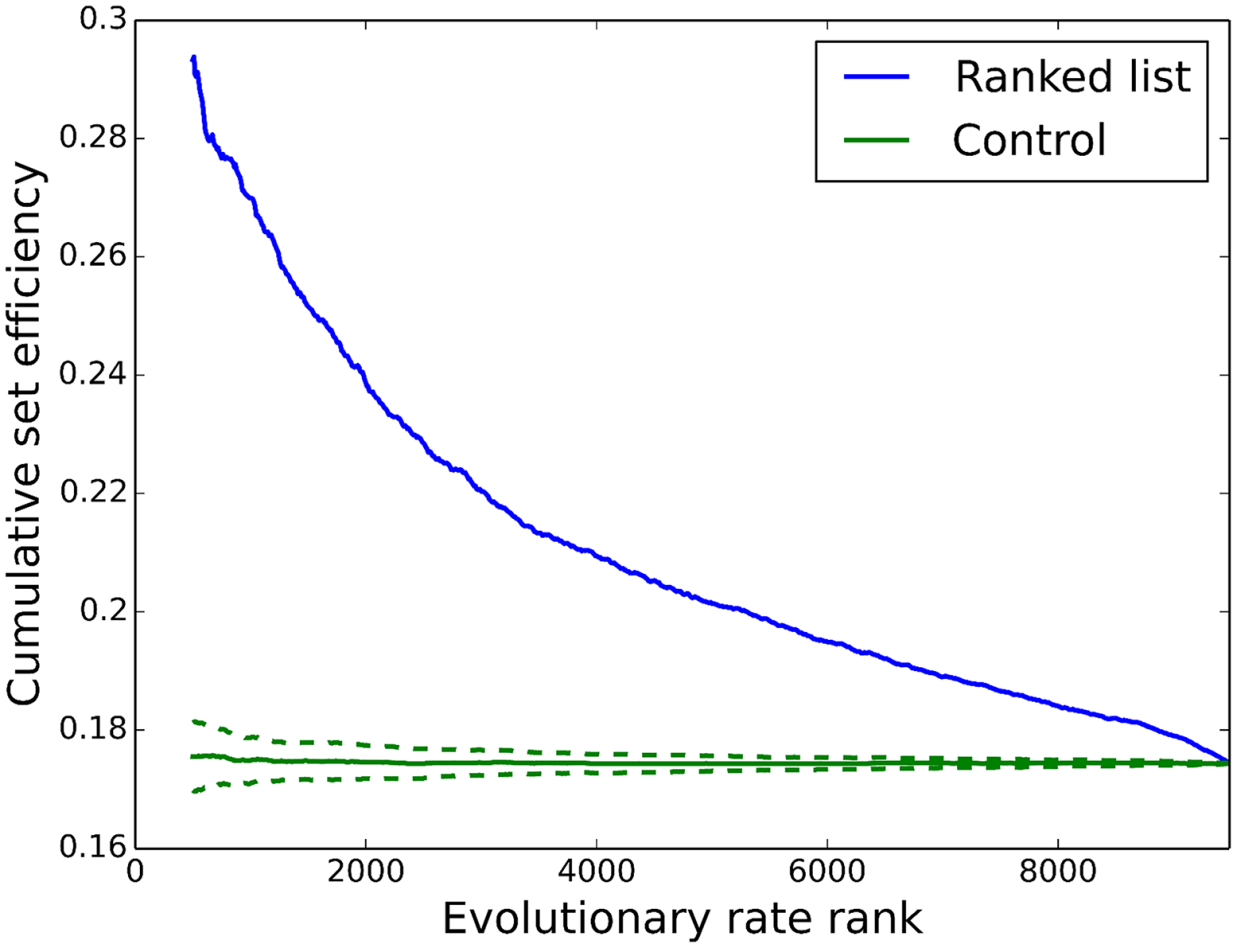
Set efficiency and evolutionary rate for AML 2.3. The cumulative set efficiency (SE) of all genes below a given evolutionary rate (ER) rank (lowest to highest ER, i.e. “coldest” to “hottest”). The SE of the 500 coldest genes is significantly higher than the control, and including hotter genes monotonically decreases the SE. This indicates that the coldest genes exchange information efficiently, while the hottest genes are more dispersed and thus communicate less efficiently.

Because of the strong correlation between a gene’s history and that of its neighbors, genes are expected to evolve in groups rather than as individuals, which should be evident in the structure of the network. The set efficiency, the mean of the inverse distance between all pairs of nodes in a set (see Methods), is shown in Fig 3 for genes in AML 2.3 ranked from coldest to hottest, and S6 Fig for genes ranked from oldest to youngest. This indicates that the oldest, coldest genes tend to be close, separated by approximately four directed edges, significantly smaller than the network average of approximately six. The set efficiency monotonically declines as hotter, younger genes are included.

**Fig 4:**
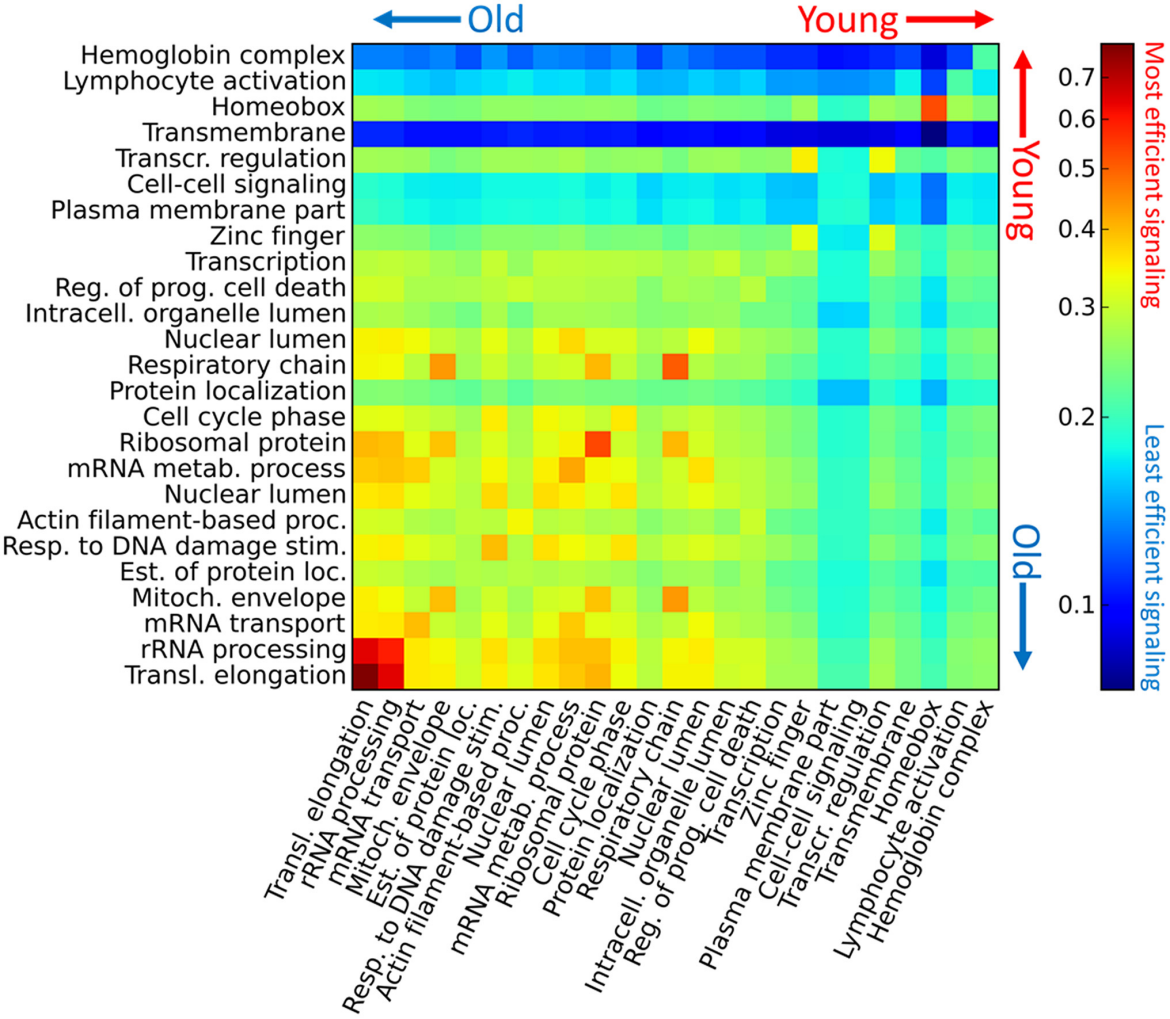
Interset efficiency and age for AML 2.3. Interset efficiency from DAVID group in column *j* to DAVID group in row *i*. The list of DAVID groups was sorted by average age from oldest (transcriptional elongation) to youngest (hemoglobin complex). Old DAVID groups exchange information efficiently, as indicated by the high interset efficiency values in the lower-left corner. Younger DAVID groups, particularly the blood cell-specific DAVID groups of lymphocyte activation and hemoglobin complex, are remote from most other DAVID groups. Note that the above matrix is asymmetric because the network is directed, and that the colors are log-scaled.

**Fig 5:**
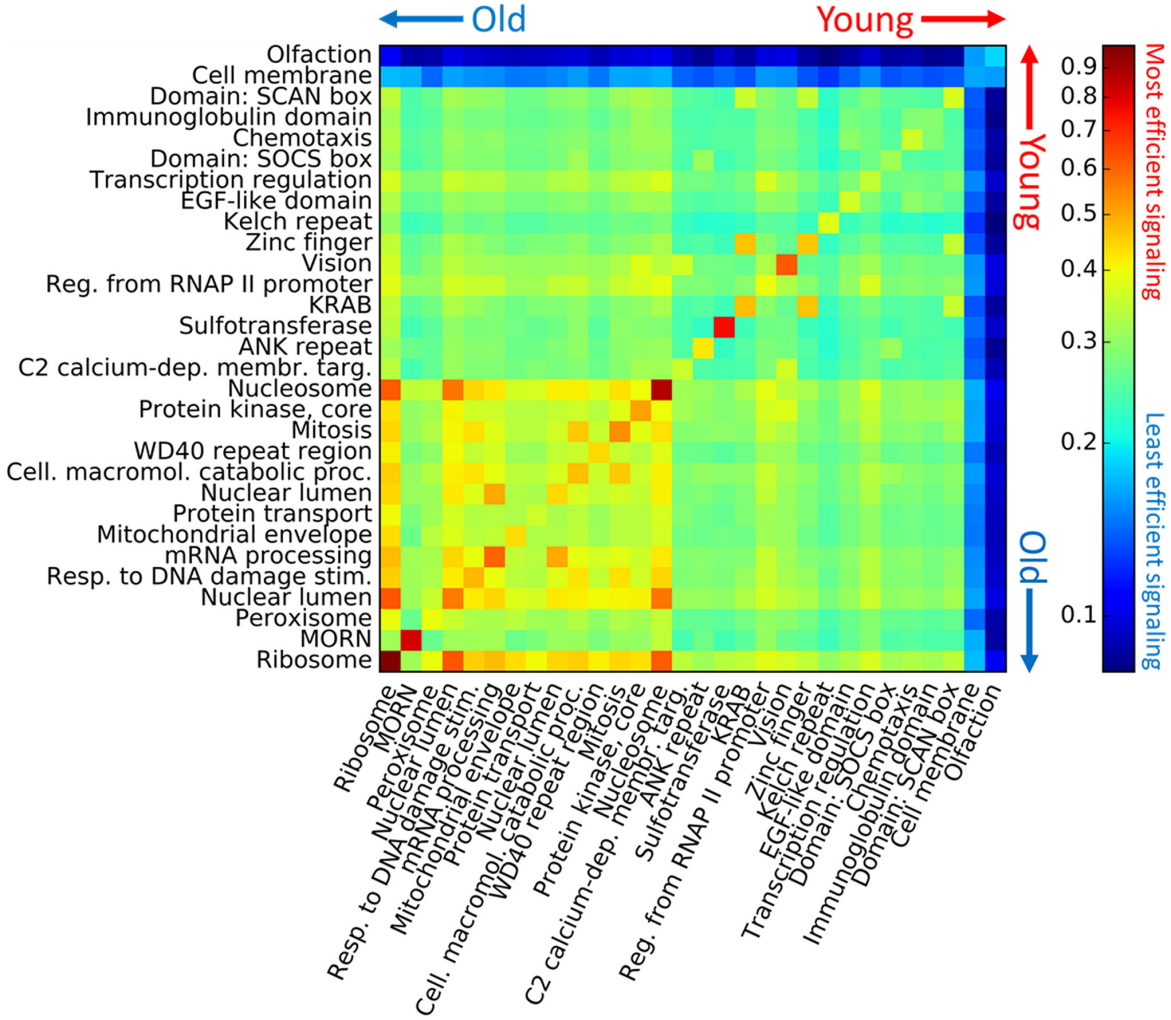
Interset efficiency and age for HumanNet. See Fig 4 for explanation.

Furthermore, the oldest DAVID groups efficiently exchange information with each other, and the youngest DAVID groups are distant from the oldest DAVID groups as well as from each other. Fig 4 shows the interset efficiency, the mean of the inverse distance from all nodes in one set to all nodes in another (see Methods), between all pairs of DAVID groups in AML 2.3, where the DAVID groups are sorted from oldest to youngest. Note that each diagonal term of the interset efficiency matrix is the set efficiency of that DAVID group. Similarly, Fig 5 shows the interset efficiency between DAVID groups in HumanNet.

Purely locally, AML 2.3 and HumanNet look quite different from one another. AML 2.3 is composed of roughly 10,000 genes and 338,000 edges, and HumanNet is composed of 14,000 genes and 876,000 edges. While they share roughly 9,000 genes, they share only 26,000 edges. However, modularity and interset efficiency, which are coarse-grained network measures that reveal the properties of sets of nodes rather than individual nodes or pairs of nodes, demonstrate that the same evolutionary signatures are present in both networks.

### Conclusion

We have shown that slowly evolving, old genes tend to interact with each other, and frequently evolving, young genes tend to interact with each other, whereas edges between those groups are less common. This naturally creates communities of genes with relatively homogeneous evolutionary attributes. Analyzing the networks in terms of communities and DAVID groups rather than single genes provided a new perspective which allowed us to establish clear relationships between network topology and evolution. The abundance of connections between old DAVID groups and the relative scarcity between old-and-young and young-and-young DAVID groups suggests that during the course of human evolution, the primitive gene regulatory network began as a core of fundamental genes and pathways. As genes duplicated and mutated, novel functions arose and eventually, through selective duplications, deletions, mutations, and rewirings, novel regulatory pathways emerged, growing outward from these ancient genes. This would place the oldest genes near the middle of the network and the youngest genes toward the periphery. These findings were mainly derived from an AML network and a general human network, and they were broadly confirmed in a normal hematopoietic network and are consistent with previous reports [13].

No gene is an island. A real understanding of the evolution of a genome only comes from studying its constituent genes in the context of the underlying complex network of interactions rather than as independent units. As network reconstruction methods continue to improve and more high quality networks become available, we expect to find more evidence of how evolution shapes the topology of gene regulatory networks.

## Methods

### Evolutionary rate and age

To compute the evolutionary rate (ER) of a gene, we first calculated the absolute ER for each amino acid position of the protein it encodes using the method from Kumar et al. [1]. Given the multiple alignment at an amino acid position in 46 species [30], its ER equals the number of different residues divided by the total evolutionary time span, based on a known phylogenetic tree [1]. The ER of a gene is the average of ERs over all amino acid positions, in units of the number of substitutions per amino acid site per billion years. The ER value ranges from ~0.011 (most conserved) for LSM2 to ~6.928 (least conserved) for CDRT15. Ages, taken from Chen et al. [2], were estimated from comparing the human genome to the genomes of 13 major clades with origins at different points along the human clade, indexed 0 (oldest) through 12 (youngest). A gene’s age was determined by searching for the earliest time at which an orthologous gene appears in an organism which branched from the human clade.

### Gene regulatory network

One gene regulatory network used in this analysis, “AML 2.3”, is a partially directed, weighted acute myeloid leukemia (AML) GRN [14]. This network was chosen primarily for its quality. It was constructed from more than 1,800 patients across 12 studies from both microarray and RNA-seq gene expression measurements in AML cells. Edges were inferred via gene expression correlation within each study, and each edge was assigned a weight based on the number of times it was detected across all studies. Edge directionality was taken from the TRANSFAC [31] and HIPPIE [32] databases. A second network was built using five studies for healthy hematopoietic stem cells (HSCs). The limited amount of data means that the HSC network is a lower confidence network than AML 2.3. Finally, the network “HumanNet” [15] was built from 21 different methods using diverse data types, including microarray co-expression, databases and mass spectrometry proteomics.

### Communities and DAVID groups

A weighted, directed, modularity-based community-finding algorithm was used to divide the genes into communities of various sizes [33]. A spy plot of the adjacency matrix after community sorting is shown in S5 Fig. The ten largest communities, indexed 0 through 9, were selected for further analysis (see Tables 1 and 2). The individual communities were then provided to the DAVID functional annotation tool to identify enriched DAVID groups in the communities [16]. The top three distinct enriched DAVID groups with Benjamini values less than 10^−4^ in each community are also included in Tables 1 and 2.

Communities and DAVID groups in Tables 1 and 2 labeled “cold” and “hot” have significantly lower and higher evolutionary rates (ERs) than the network average, respectively. Likewise, groups of genes labeled “old” and “young” are significantly older and younger than the network’s average age, respectively. A one-tailed significance level of *p* < 10^−3^ in the difference from the mean was chosen for both ER and age. The Kolmogorov-Smirnov (KS) statistic and p-value were also computed for each community and DAVID group to quantify the difference between the distribution of all genes and the distribution of each set of genes. KS statistics are reported in S1A Table. Some example DAVID group distributions are shown in Fig 1A and 1B, and all distributions are shown in S7 and S8 Figs for ERs and ages, respectively. A summary of the ERs and ages of the enriched DAVID groups in Table 1 is shown in Fig 1C. The same analysis was conducted for normal hematopoietic stem cell network built from five studies (GSE48846, 2666, 33223, 24759, and 30376) using the same method as for AML 2.3, with the data reported in S1A Table.

### ERs and ages between interacting genes

To determine the significance of the correlation between ERs and gene-gene interactions, the difference in evolutionary rates between all gene pairs connected by an edge was computed for AML 2.3 as well as for degree-preserving randomizations of AML 2.3. S1 Fig shows the distribution of (*ER*_*j*_ − *ER*_*i*_) for all gene pairs (*i*,*j*) which are connected by an edge *j*→*i* in AML 2.3 (green distribution), as well as for all pairs of genes in one degree-preserving randomization of the same network (purple distribution). Note that the distributions are asymmetric because AML 2.3 is a directed network. The real distribution of ER differences has a smaller standard deviation than for the randomized network, meaning that difference in evolutionary rates between interacting genes is small on average, in agreement with Fraser et al. [9]. To quantify the significance of this difference, AML 2.3 was randomized 20,000 times and the standard deviation of each set of ER differences was recorded, as shown in S2 Fig. This gave a z-score of –96.8 for the ER differences in the real network. Since none of the sampled randomized networks had an ER difference width less than that of the real network, an upper limit of 5.0 × 10^−5^ was placed on the p-value. The same procedure was used to find the significance in the age difference between connected genes, which resulted in a z-score of –72.0 and an upper limit of 5.0 × 10^−5^ for the p-value (see S3 and S4 Figs).

### Global, set, and interset efficiency

The global efficiency [34] of a network is defined as

$$E_{global}=\frac{1}{n\left(n-1\right)}\sum_{i\neq j}\frac{1}{d_{ij}}$$

where *n* is the number of nodes in the network, *d*_*ij*_ is the distance from node *j* to node *i*, and 0 ≤ *E*_*global*_ ≤ 1 for unweighted networks. We define the set efficiency (SE) of a set of nodes *M* as

$$E_{M}=\frac{1}{\left|M\right|\left(\left|M\right|-1\right)}\sum_{\begin{array}{c} i,j\in M,\\ i\neq j \end{array}}\frac{1}{d_{ij}}$$

where |*M*| is the number of nodes in *M*, and 0 ≤ *E*_*M*_ ≤ 1 for unweighted networks. *E*_*M*_ > *E*_*global*_ implies that nodes in *M* are closer to each other than average in the network, and *E*_*M*_ < *E*_*global*_ implies that the nodes are more dispersed than average. Note that *d*_*ij*_ is calculated using the full network, so shortest paths from *j* to *i* may pass through nodes which are not in *M*. The SE was used to examine the topological distribution of ERs and ages in AML 2.3. The ERs were sorted from coldest to hottest, and the SE of the first 500 genes was computed, increasing the window size in steps of 10 genes from the beginning to the end of the ER list (i.e. the 500 coldest, 510 coldest, etc.). The resulting curve is shown in blue in Fig 3. As a control, the order of the genes was randomized (but the underlying network, AML 2.3, remained unchanged) and the SE was computed for the first 500 genes in the randomized list, then the first 510 genes, etc. in steps of 10. 100 of these curves were generated. Fig 3 shows the mean of these 100 controls (solid green line) plus/minus one standard deviation (dashed green lines). See S6 Fig for the same plot using age rather than ER.

We define the interset efficiency (IE) from node set *J* to node set *I* as

$$E_{IJ}=\frac{1}{\left|I\right|\left|J\right|-\left|I\cap J\right|}\sum_{\begin{array}{c} i\in I,j\in J,\\ i\neq j \end{array}}\frac{1}{d_{ij}}$$

where |*I* ∩ *J*| is the number of nodes shared by sets *I* and *J*, and 0 ≤ *E*_*IJ*_ ≤ 1 for unweighted networks. As with the set efficiency, shortest paths may pass through nodes which are neither in *I* nor *J*. Note that this formulation is defined when sets *I* and *J* have a non-empty intersection, and that the diagonal terms of the interset efficiency reduce to the set efficiency, i.e. *E*_*II*_ = *E*_*I*_. A large *E*_*IJ*_ implies that the average distance from nodes in *J* to nodes in *I* is small, and a small *E*_*IJ*_ implies large distances. *E*_*IJ*_ is asymmetric for directed networks. This measure was used in Figs 4 and 5 to quantify the proximity of the DAVID groups from Tables 1 and 2, respectively. See S4 File for a more detailed explanation of the interset efficiency.

## Supporting Information

S1 Fig Genes linked by edges share similar evolutionary rates. Distribution of *ER*_*j*_ − *ER*_*i*_ for all pairs of genes linked by an edge in AML 2.3 (green) and for a degree-preserving randomization of AML 2.3 (purple). The integral of each distribution was normalized to 1. The width of the difference in ER for the real network is much small than that of the randomized network, indicating that hot genes tend to connect with hot genes, and cold with cold.
(PNG)

S2 Fig Significance of difference in evolutionary rates. Histogram showing the width (i.e. the standard deviation) of the purple distribution in S1 Fig for 20,000 degree-preserving randomizations of AML 2.3. The width of the difference in ER distribution for the real AML 2.3 network (the green distribution in S1 Fig) is 0.76, located 96.8 standard deviations to the left of the above distribution. This demonstrates that the ERs of genes in AML 2.3 are strongly correlated with those of their neighbors.
(PNG)

S3 Fig Genes linked by edges share similar ages. Distribution of *age*_*j*_ − *age*_*i*_ for all pairs of genes linked by an edge in AML 2.3 (green) and for a degree-preserving randomization of AML 2.3 (purple). The integral of each distribution was normalized to 1. The width of the difference in age for the real network is much small than that of the randomized network, indicating that young genes tend to connect with young genes, and old with old.
(PNG)

S4 Fig Significance of difference in ages. Histogram showing the width (i.e. the standard deviation) of the purple distribution in S3 Fig for 20,000 degree-preserving randomizations of AML 2.3. The width of the difference in age distribution for the real AML 2.3 network (the green distribution in S2 Fig) is 4.02, located 72.0 standard deviations to the left of the above distribution. This demonstrates that the ages of genes in AML 2.3 are strongly correlated with those of their neighbors.
(PNG)

S5 Fig Spy plot of community-sorted adjacency matrix. Spy plot of the adjacency matrix *W*_*ij*_ for AML 2.3 after sorting nodes by community size (from smallest to largest), and after sorting the nodes in each community by the genes’ outdegrees (from largest to smallest). A black dot in row *i*, column *j* means *W*_*ij*_ ≠ 0. Communities 0–9 are boxed in red, forming the diagonal blocks of the matrix. Intracommunal edges are grouped along the block diagonal, and intercommunal edges are off-block diagonal.
(PNG)

S6 Fig Set efficiency as a function of age. Cumulative set efficiency (solid blue) for nodes ranked from oldest to youngest in AML 2.3. As in Fig 3, the set efficiency was computed for the 500 youngest genes, and then the first 510 genes, etc. in steps of 10 until all genes were included. The control (solid green) plus/minus one standard deviation (dashed green) was computed by randomizing the order of genes 100 times and computing the cumulative set efficiency of the randomized lists.
(PNG)

S7 Fig Evolutionary rate distributions. These figures show the distribution of evolutionary rates for all communities and DAVID groups reported in Table 1.
(PDF)

S8 Fig Age distributions. These figures show the distribution of ages for all communities and DAVID groups reported in Table 1.
(PDF)

S1 File Centrality measures vs. evolutionary rates and ages across enriched DAVID groups. (XLSX)

S2 File Gene ages. See Methods for details.
(TXT)

S3 File Gene evolutionary rates. See Methods for details.
(TXT)

S4 File Interset efficiency derivation/explanation. (PDF)

S1 Table Detailed analysis of DAVID groups in AML 2.3, HumanNet, and the normal hematopoietic stem cell network. (XLSX)

S2 Table Comparison between DAVID group enrichment in real and random communities. (XLSX)
