# Supplementary figures and images for: Evolutionary and Topological Properties of Genes and Community Structures in Human Gene Regulatory Networks
Source: PLoS Comput Biol. 2016 Jun 30;12(6):e1005009. doi: 10.1371/journal.pcbi.1005009 (PMC4928929; doi:10.1371/journal.pcbi.1005009)

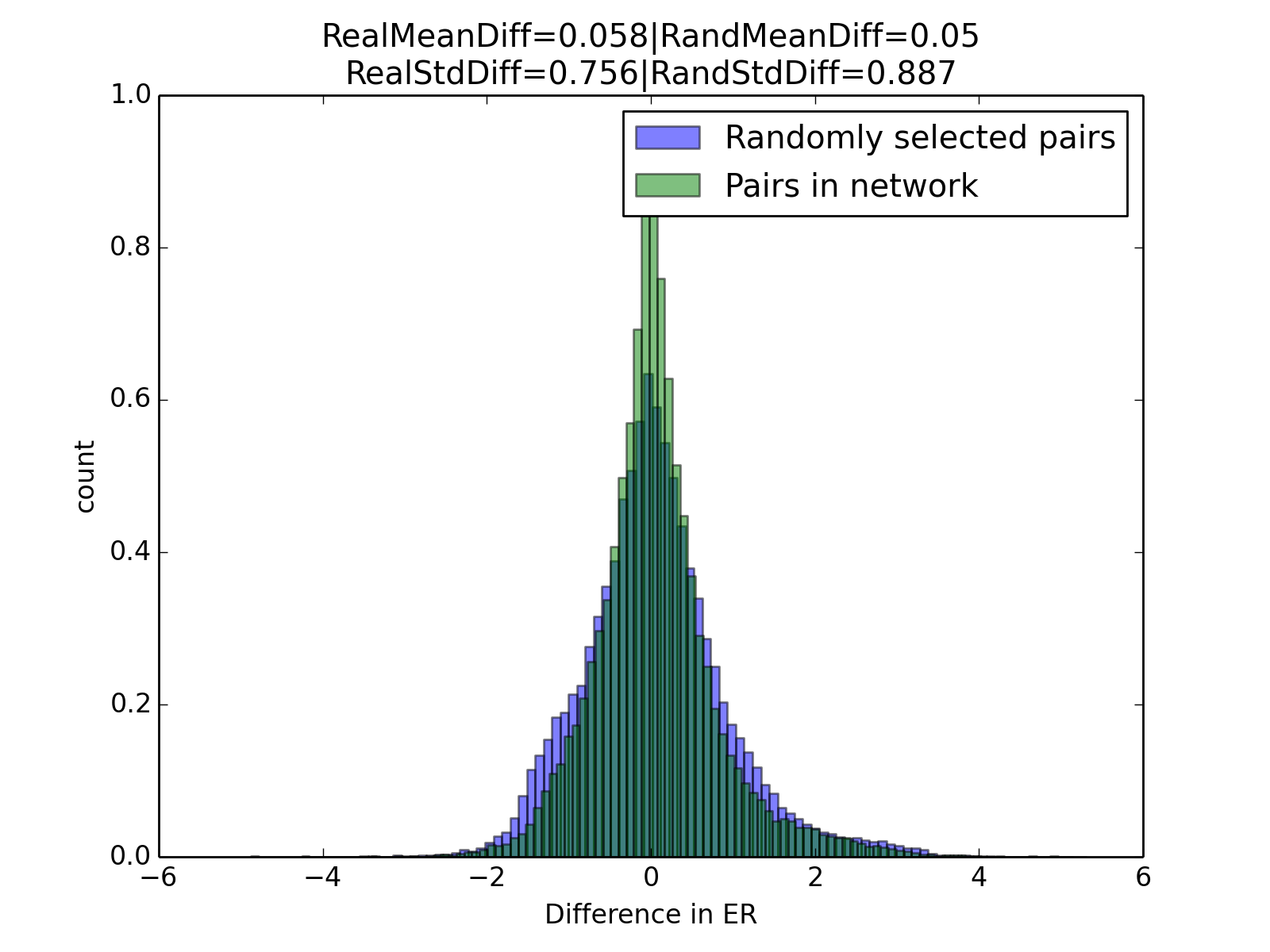

Supplement: S1 Fig — Distribution of ERj − ERi for all pairs of genes linked by an edge in AML 2.3 (green) and for a degree-preserving randomization of AML 2.3 (purple). The integral of each distribution was normalized to 1. The width of the difference in ER for the real network is much small than that of the randomized network, indicating that hot genes tend to connect with hot genes, and cold with cold. (PNG) [file pcbi.1005009.s001.png]

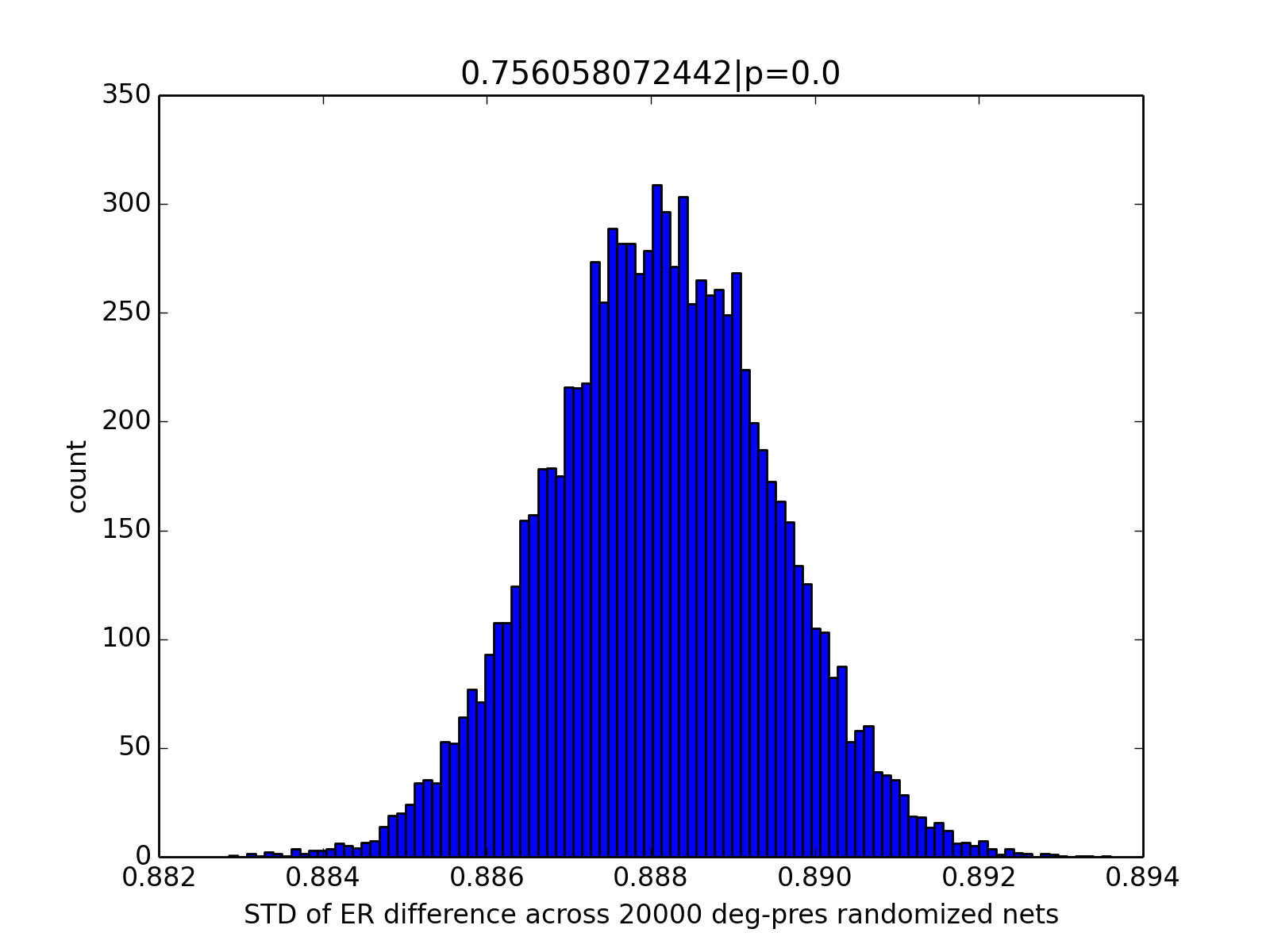

Supplement: S2 Fig — Histogram showing the width (i.e. the standard deviation) of the purple distribution in S1 Fig for 20,000 degree-preserving randomizations of AML 2.3. The width of the difference in ER distribution for the real AML 2.3 network (the green distribution in S1 Fig) is 0.76, located 96.8 standard deviations to the left of the above distribution. This demonstrates that the ERs of genes in AML 2.3 are strongly correlated with those of their neighbors. (PNG) [file pcbi.1005009.s002.png]

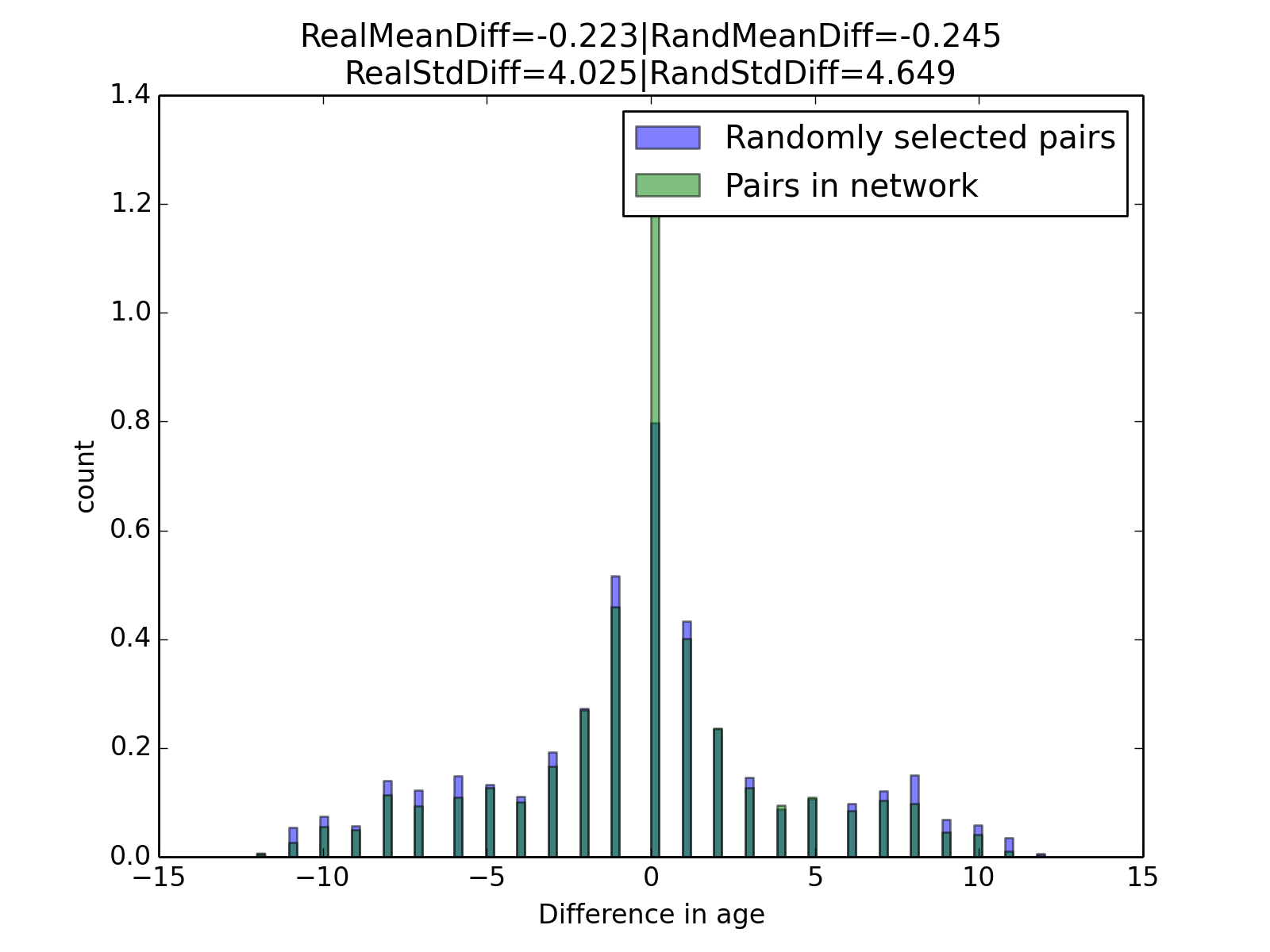

Supplement: S3 Fig — Distribution of agej − agei for all pairs of genes linked by an edge in AML 2.3 (green) and for a degree-preserving randomization of AML 2.3 (purple). The integral of each distribution was normalized to 1. The width of the difference in age for the real network is much small than that of the randomized network, indicating that young genes tend to connect with young genes, and old with old. (PNG) [file pcbi.1005009.s003.png]

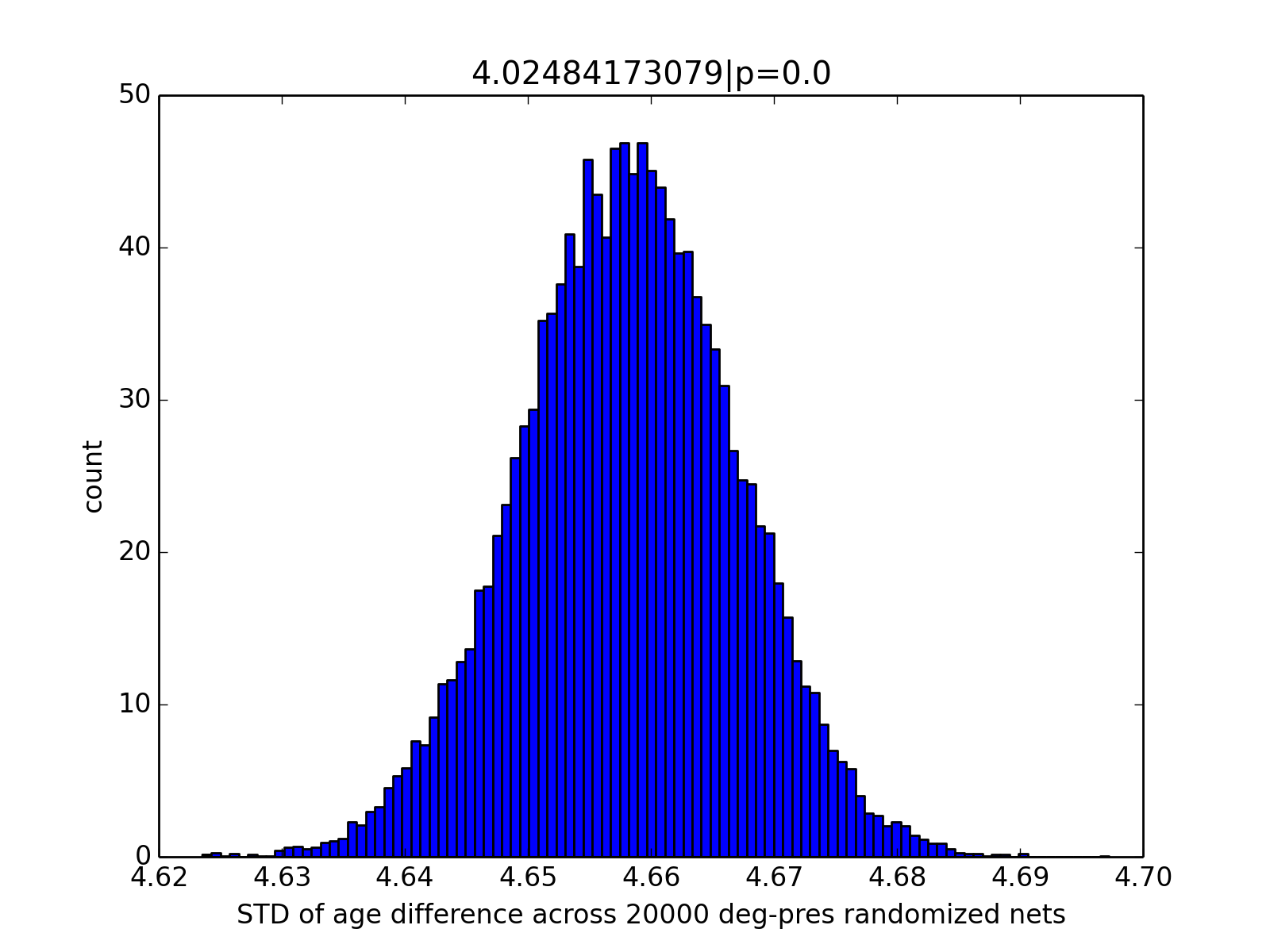

Supplement: S4 Fig — Histogram showing the width (i.e. the standard deviation) of the purple distribution in S3 Fig for 20,000 degree-preserving randomizations of AML 2.3. The width of the difference in age distribution for the real AML 2.3 network (the green distribution in S2 Fig) is 4.02, located 72.0 standard deviations to the left of the above distribution. This demonstrates that the ages of genes in AML 2.3 are strongly correlated with those of their neighbors. (PNG) [file pcbi.1005009.s004.png]

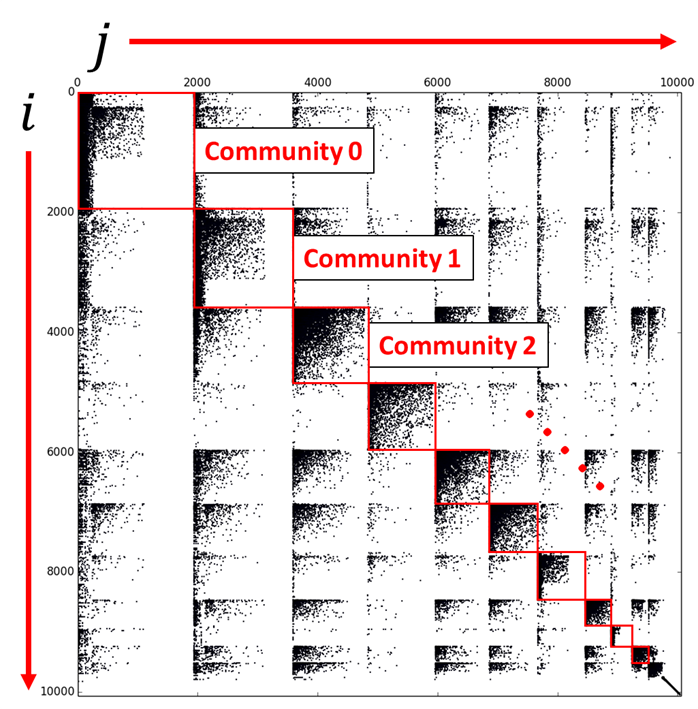

Supplement: S5 Fig — Spy plot of the adjacency matrix Wij for AML 2.3 after sorting nodes by community size (from smallest to largest), and after sorting the nodes in each community by the genes’ outdegrees (from largest to smallest). A black dot in row i, column j means Wij ≠ 0. Communities 0–9 are boxed in red, forming the diagonal blocks of the matrix. Intracommunal edges are grouped along the block diagonal, and intercommunal edges are off-block diagonal. (PNG) [file pcbi.1005009.s005.png]

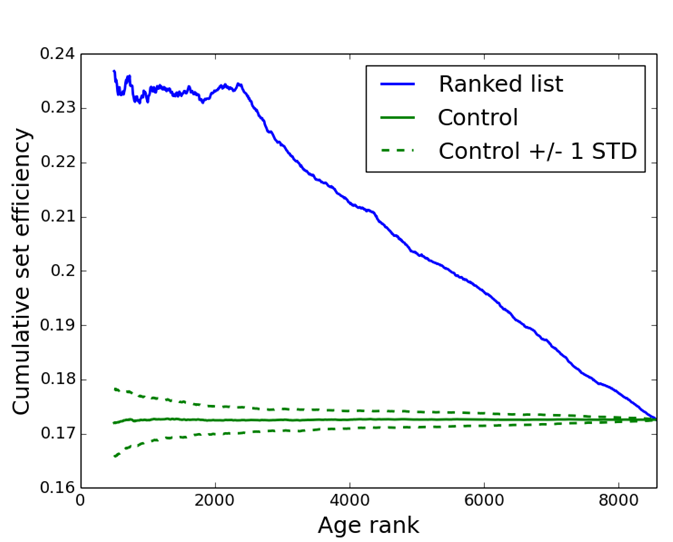

Supplement: S6 Fig — Cumulative set efficiency (solid blue) for nodes ranked from oldest to youngest in AML 2.3. As in Fig 3, the set efficiency was computed for the 500 youngest genes, and then the first 510 genes, etc. in steps of 10 until all genes were included. The control (solid green) plus/minus one standard deviation (dashed green) was computed by randomizing the order of genes 100 times and computing the cumulative set efficiency of the randomized lists. (PNG) [file pcbi.1005009.s006.png]

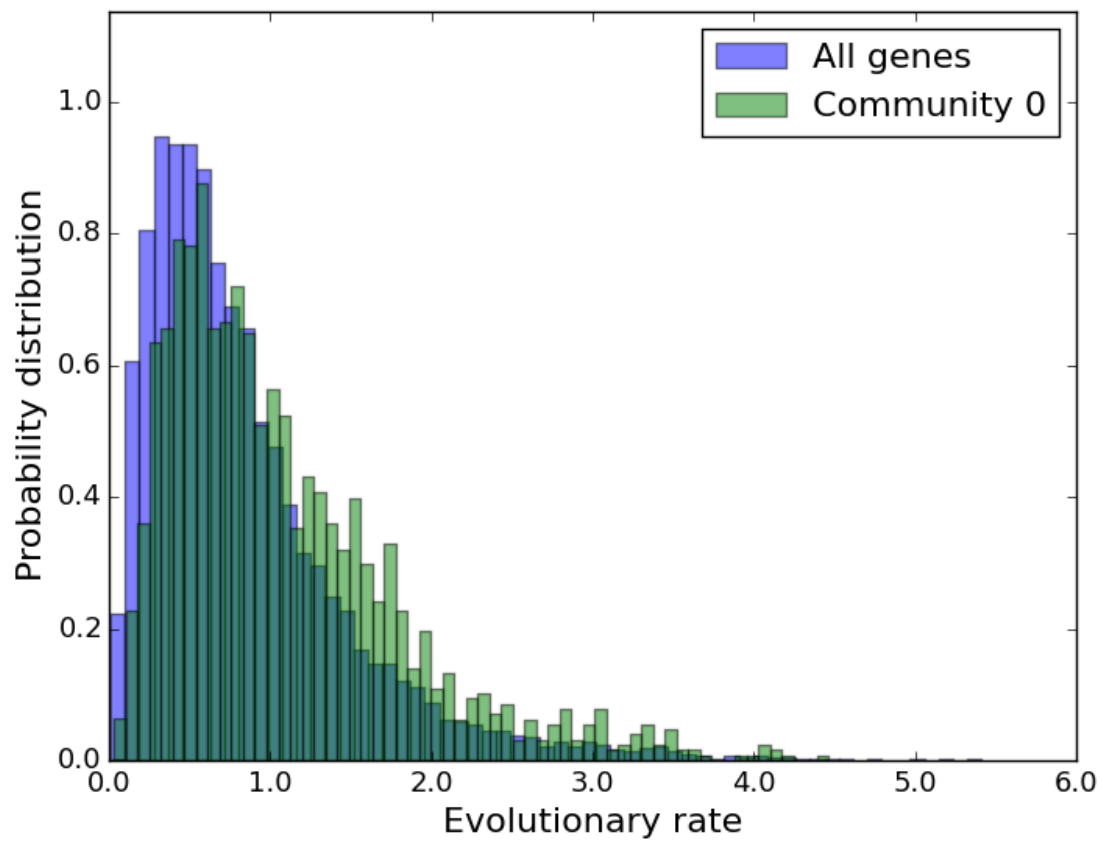

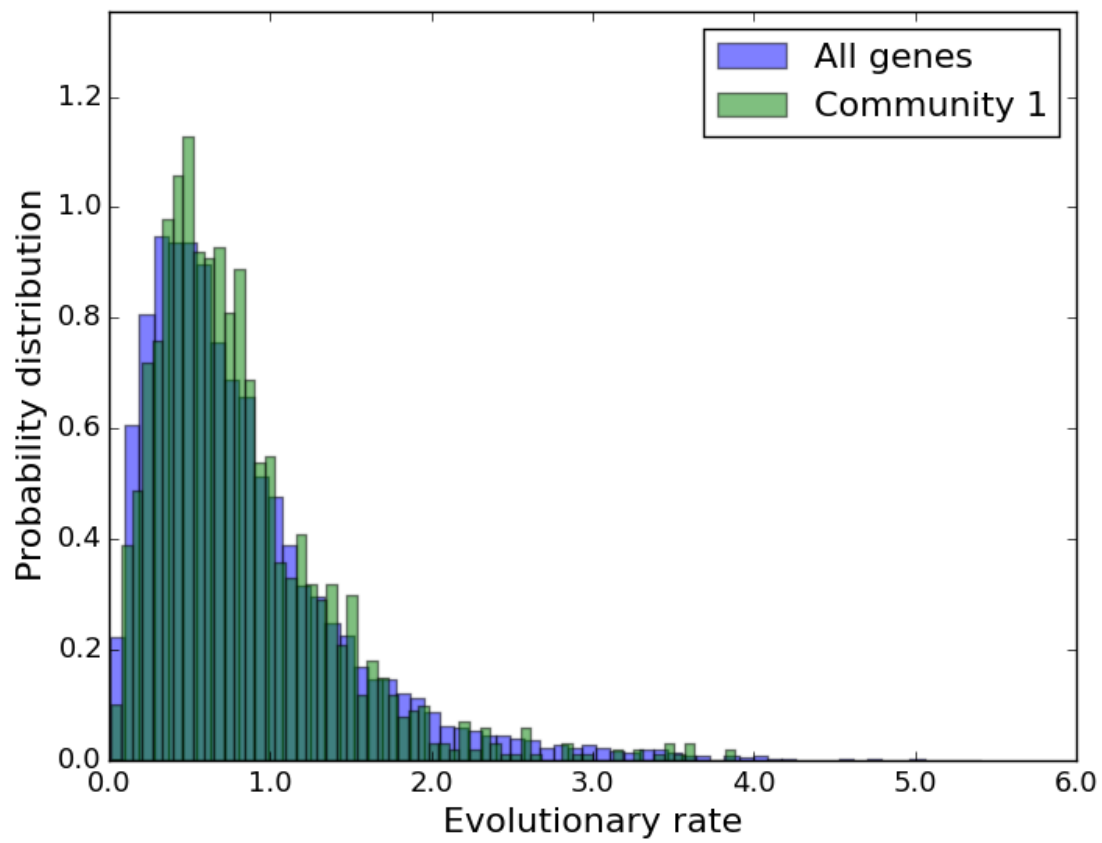

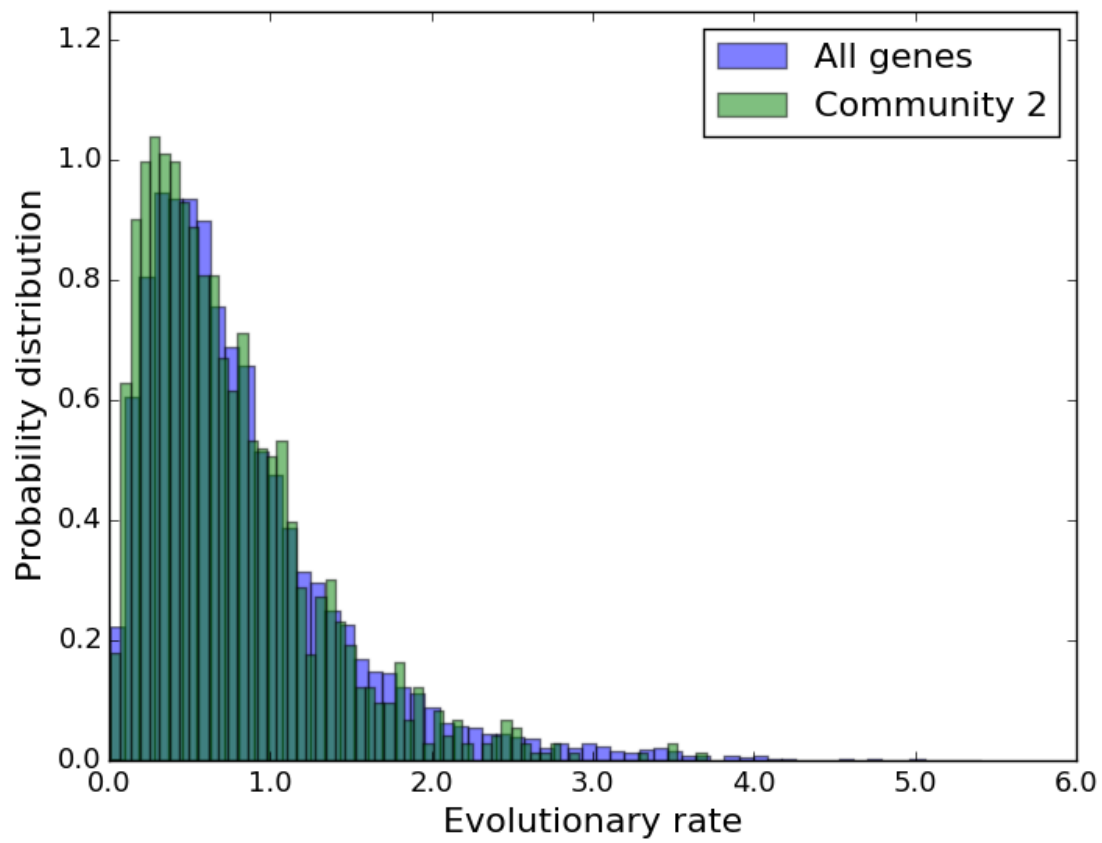

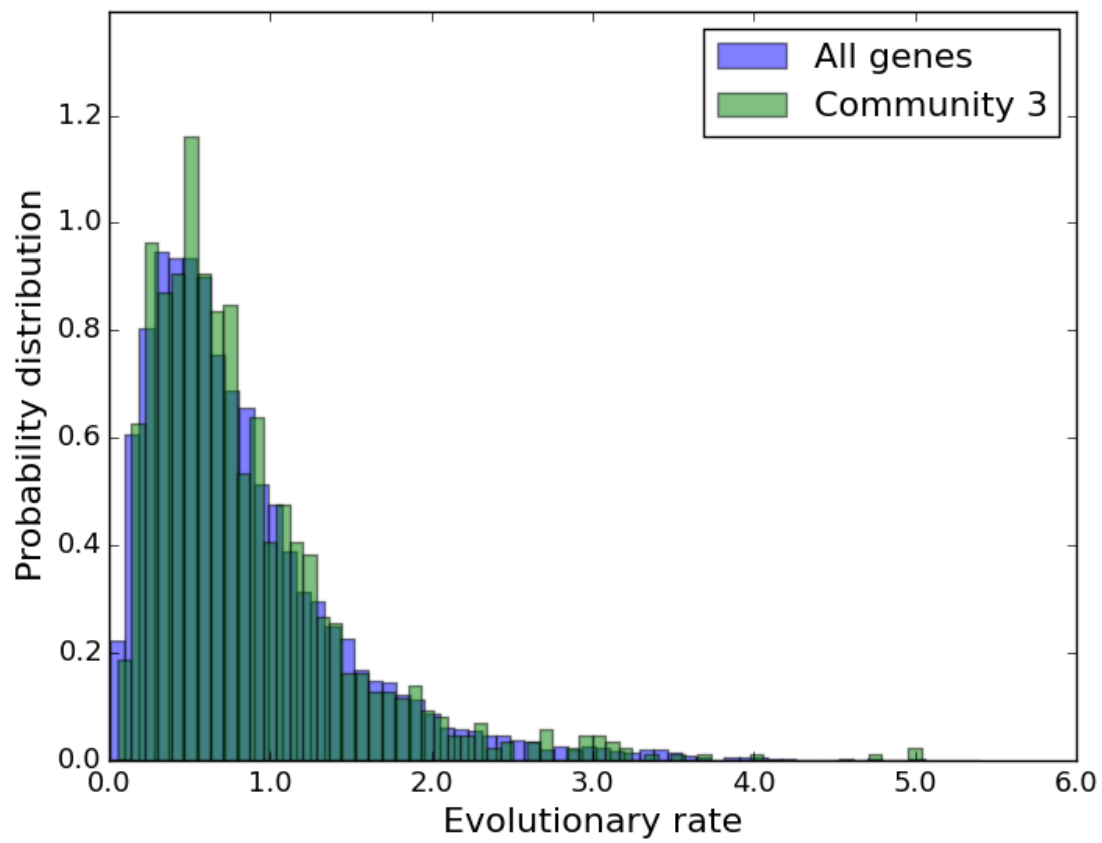

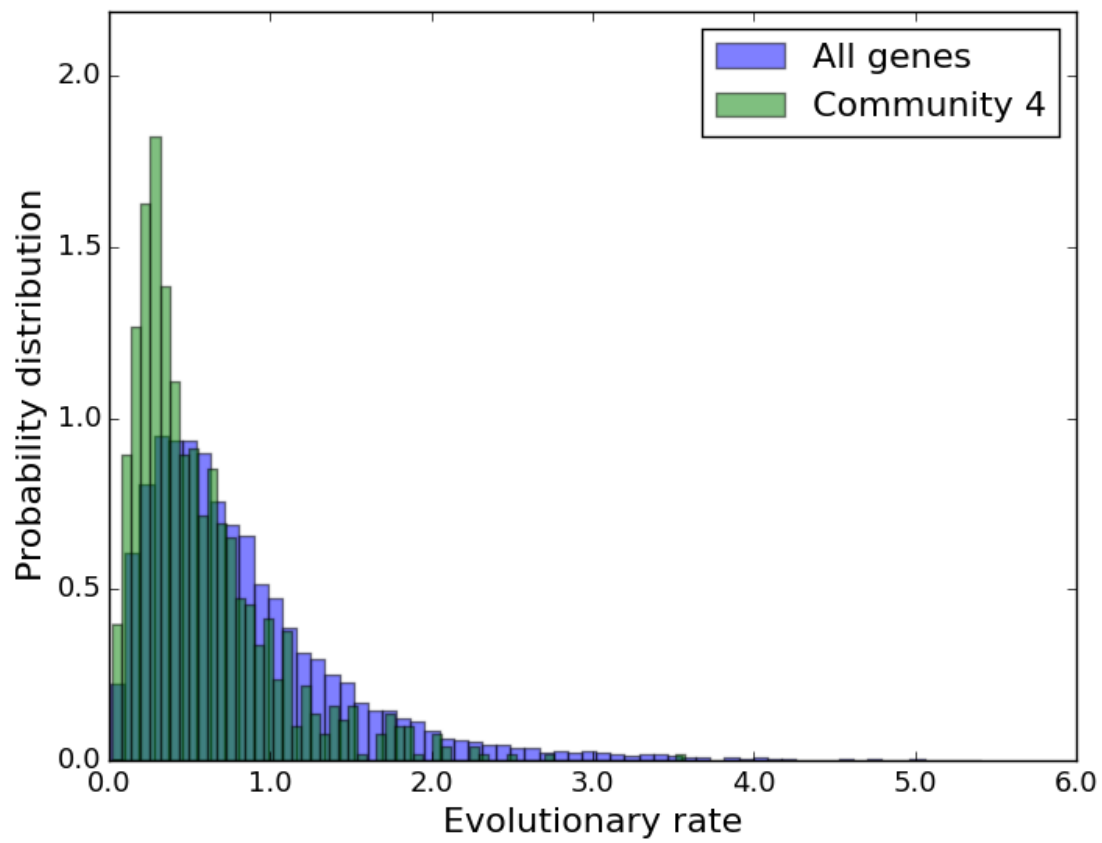

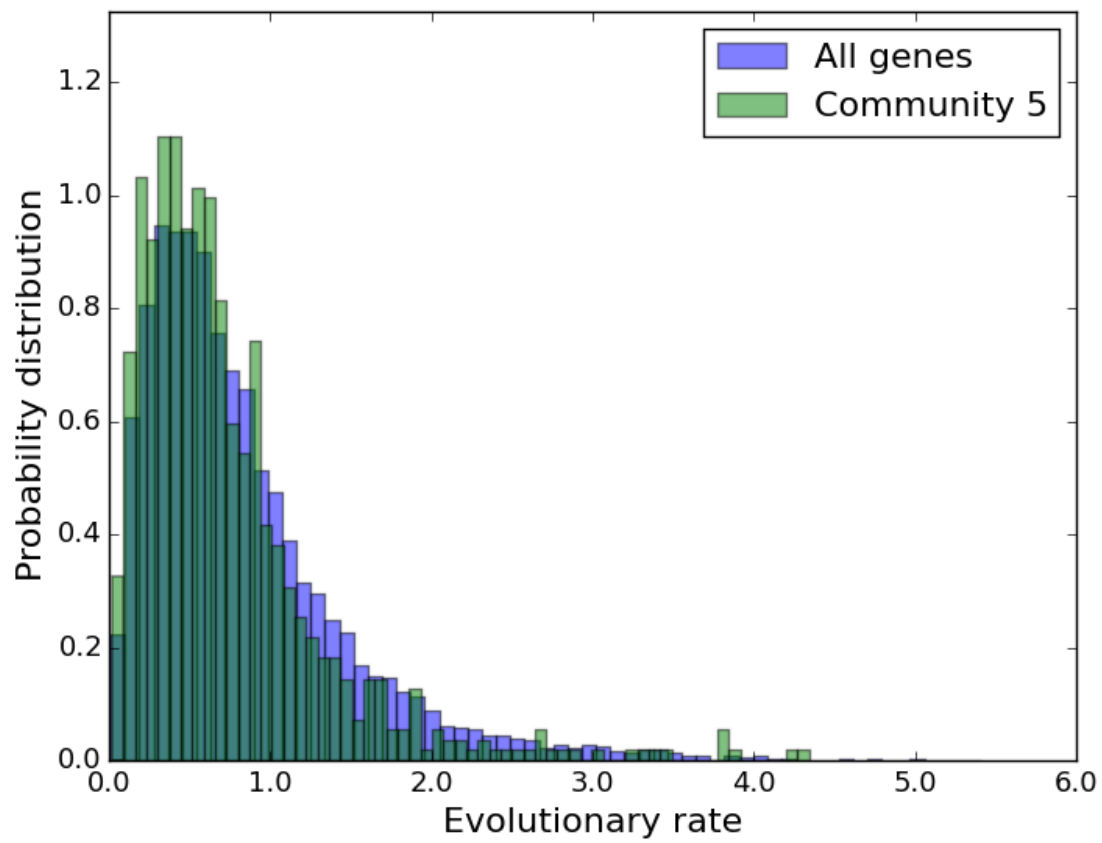

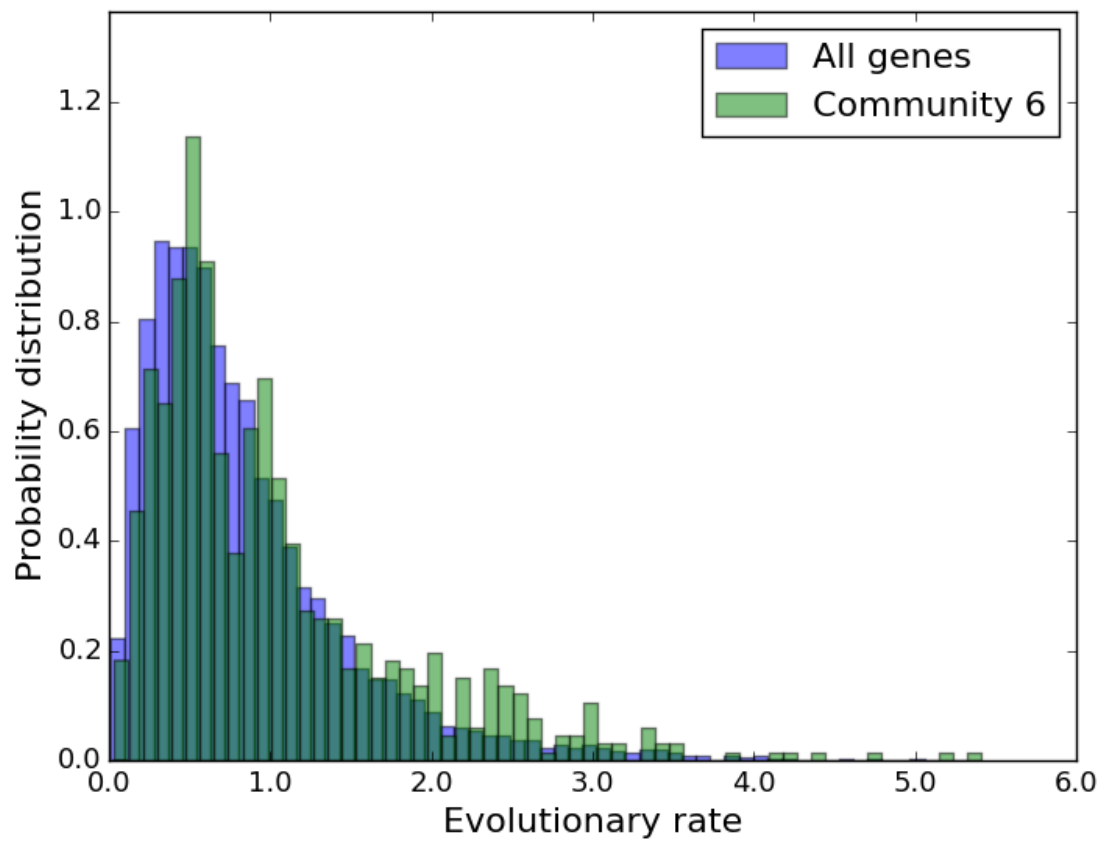

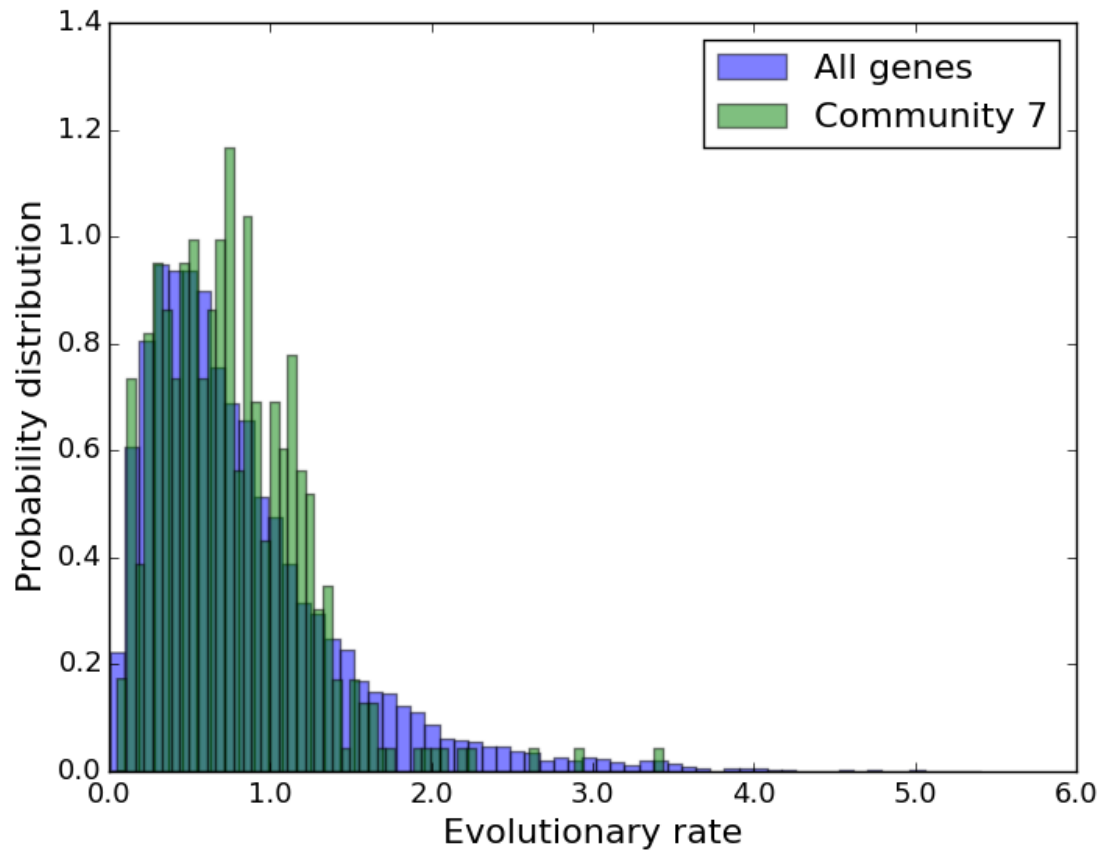

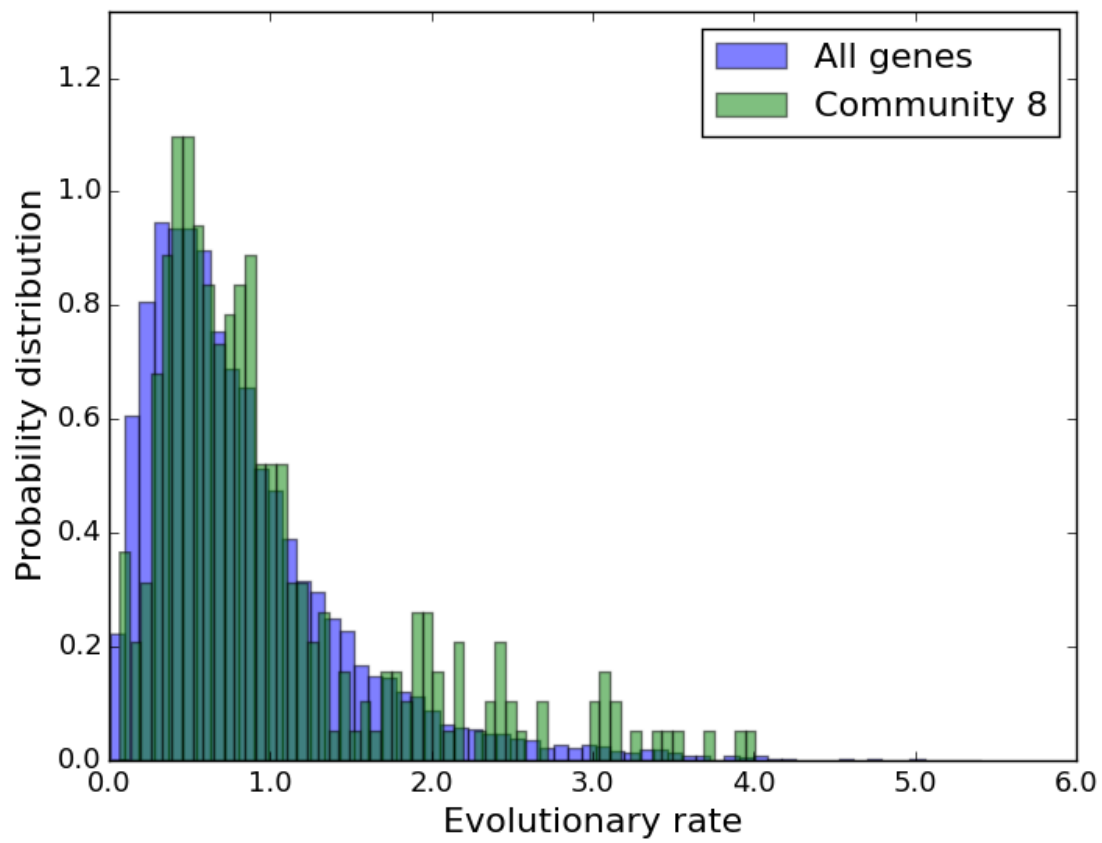

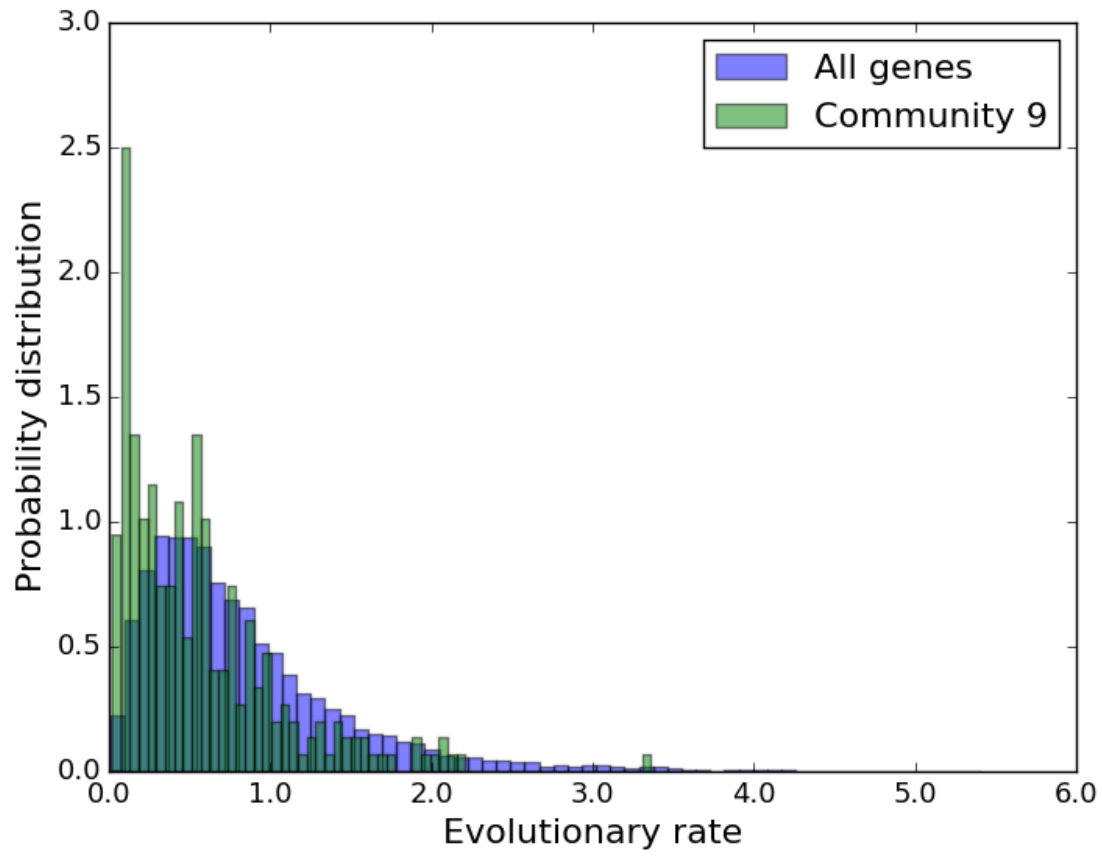

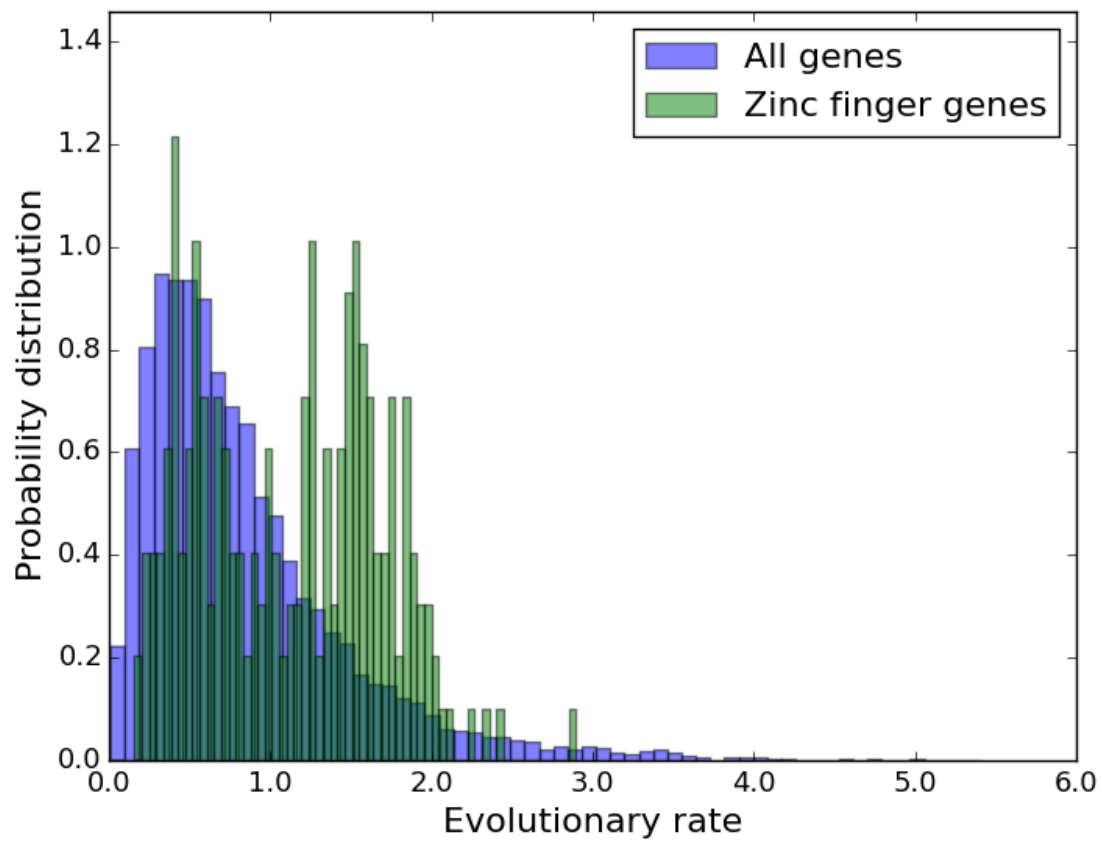

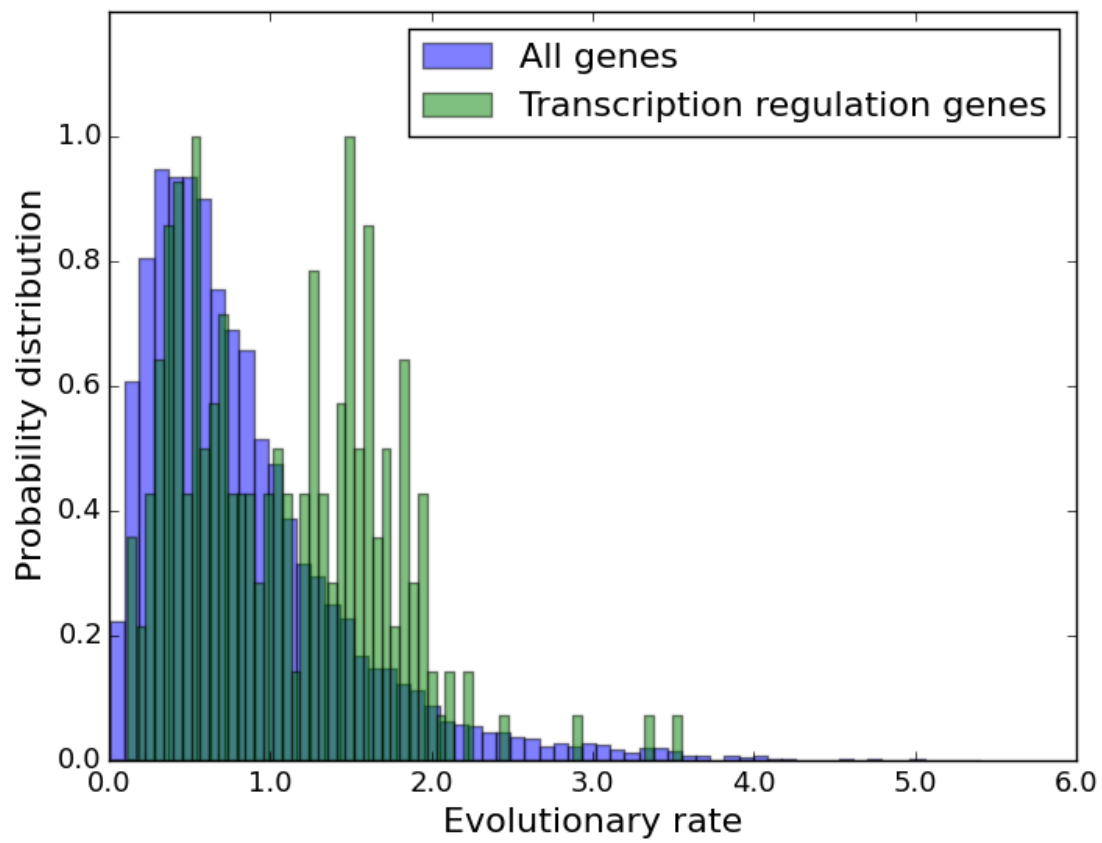

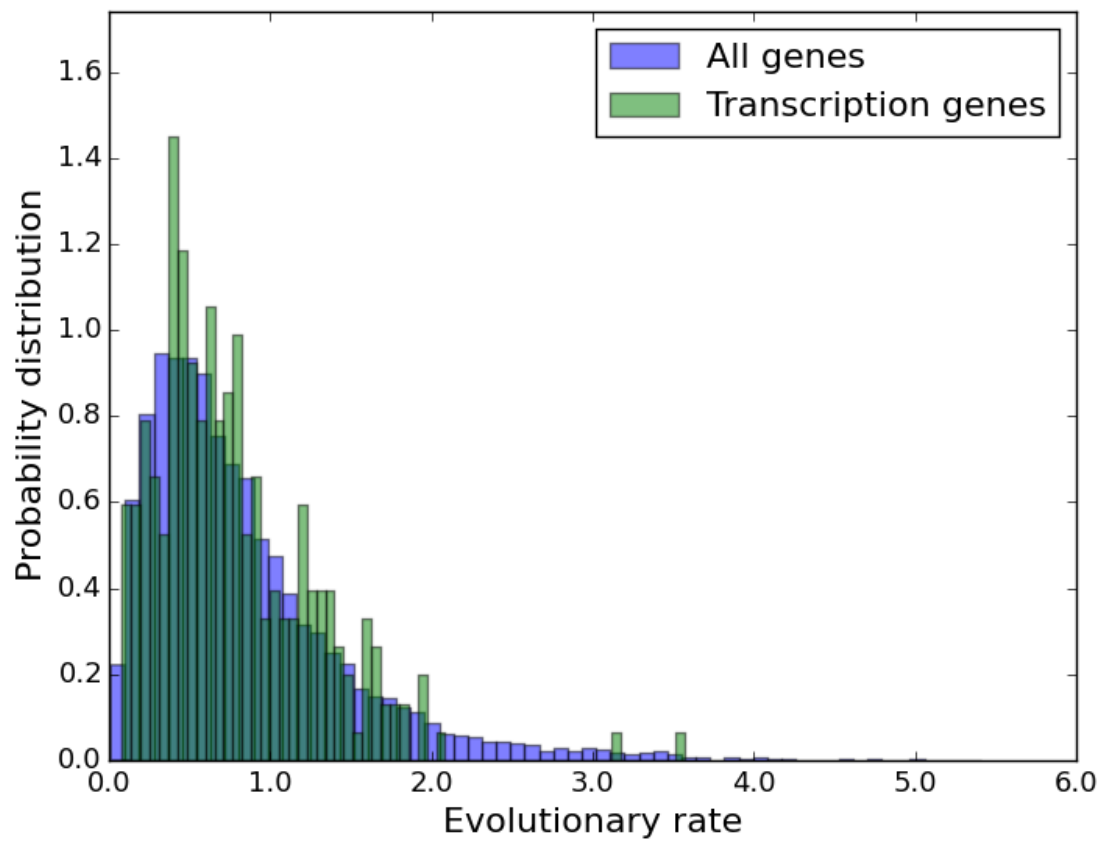

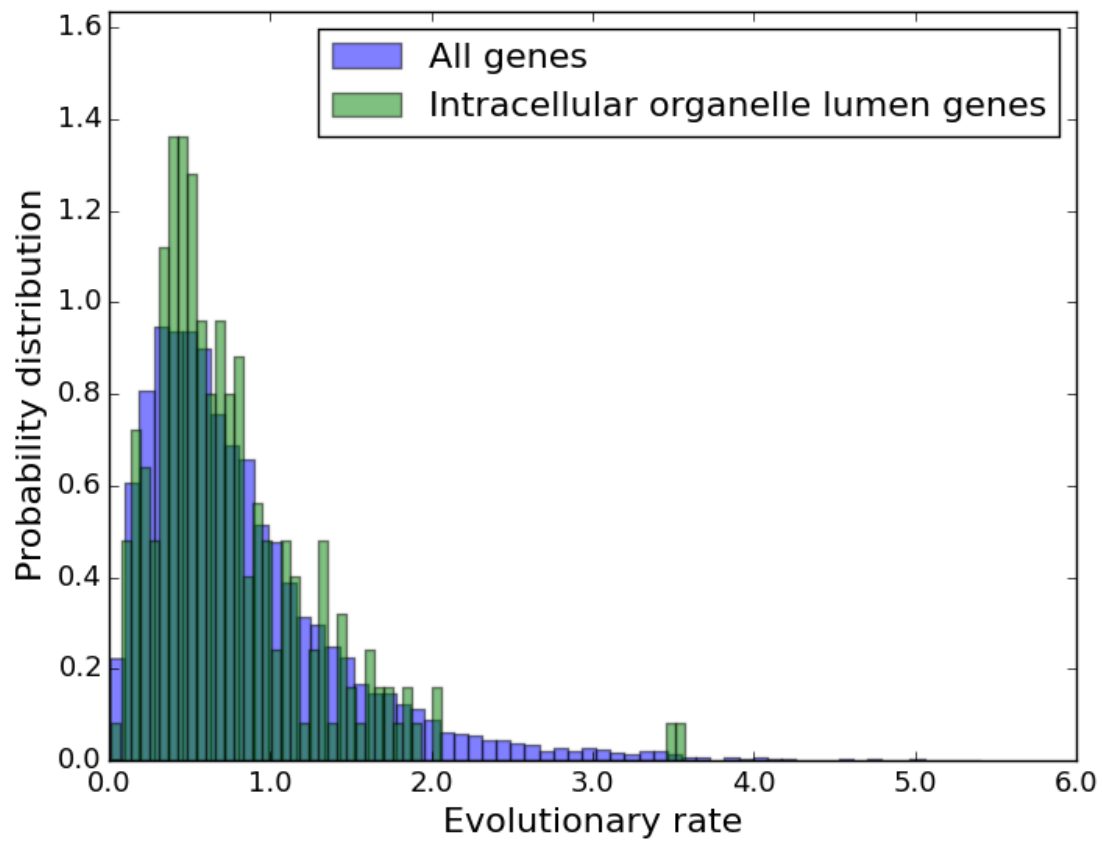

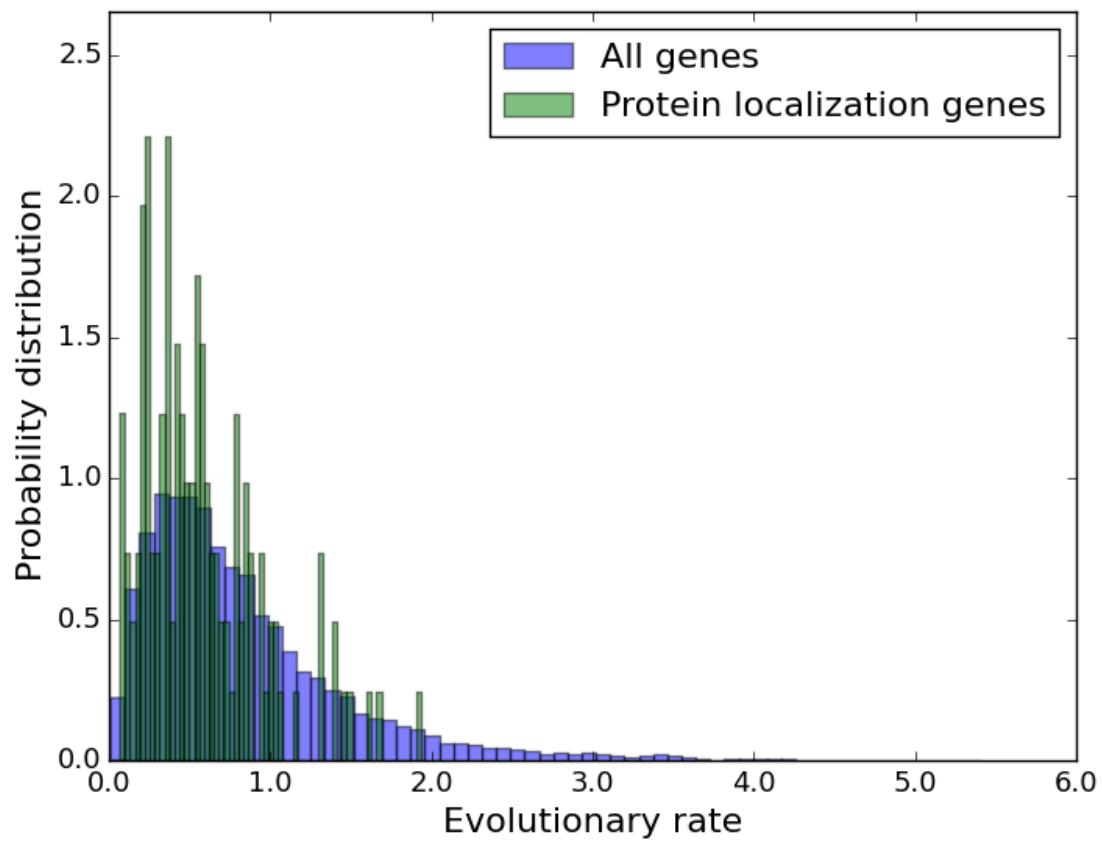

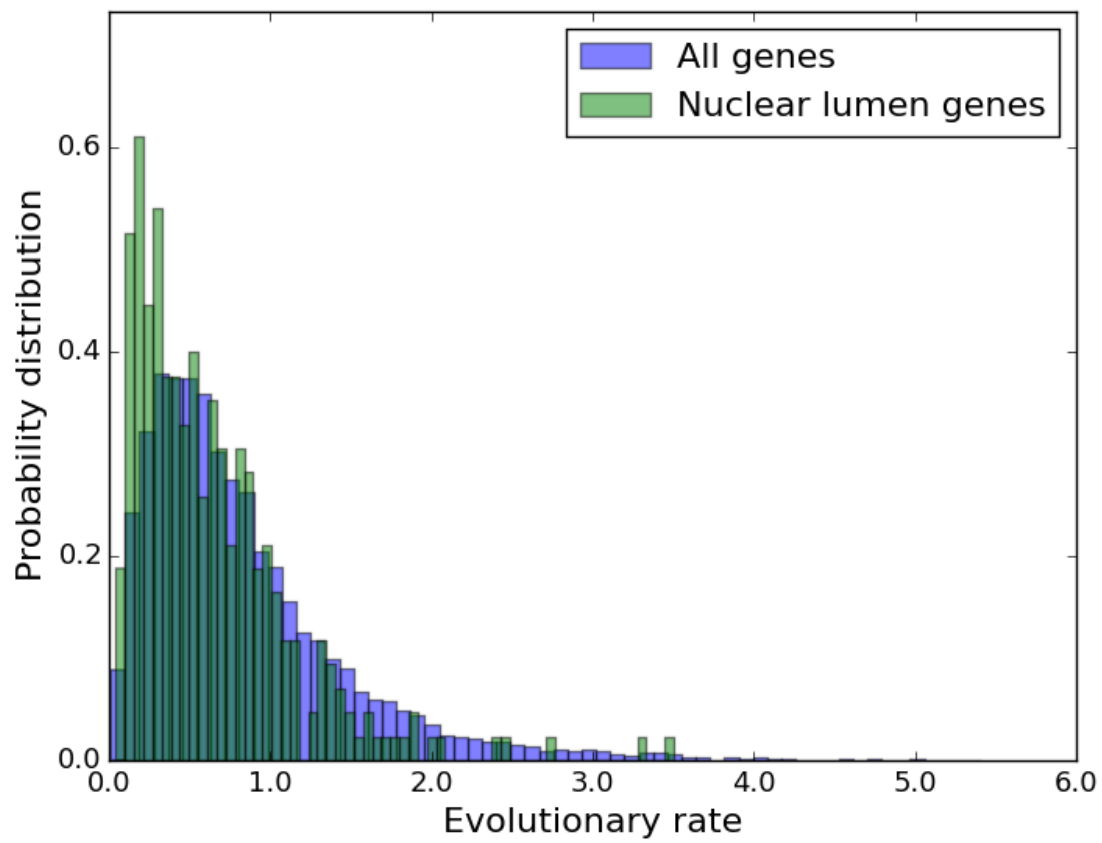

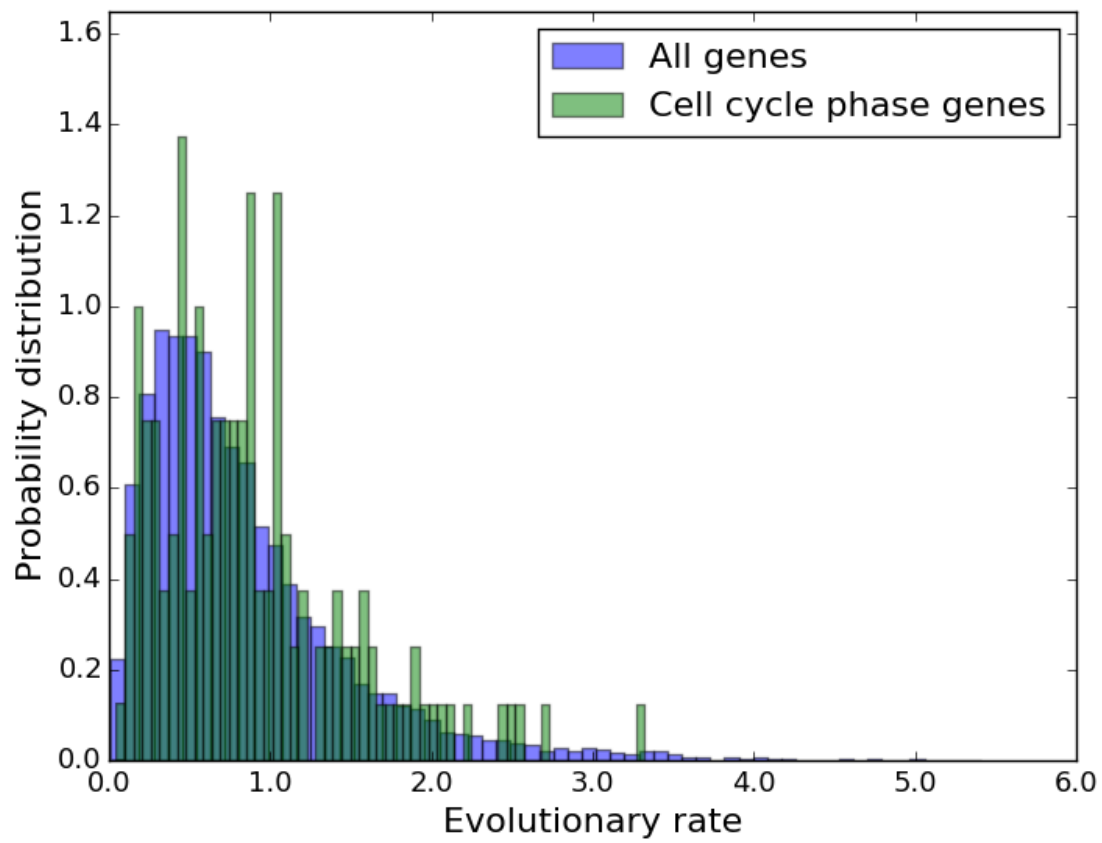

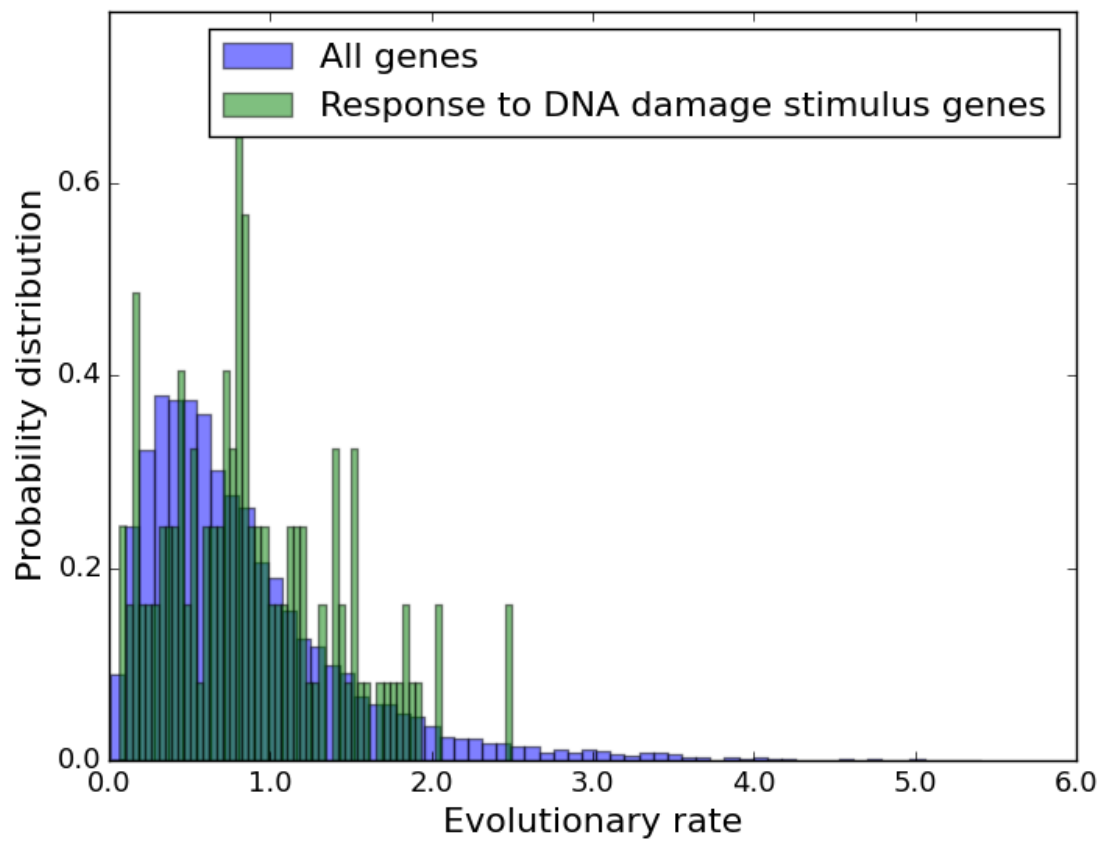

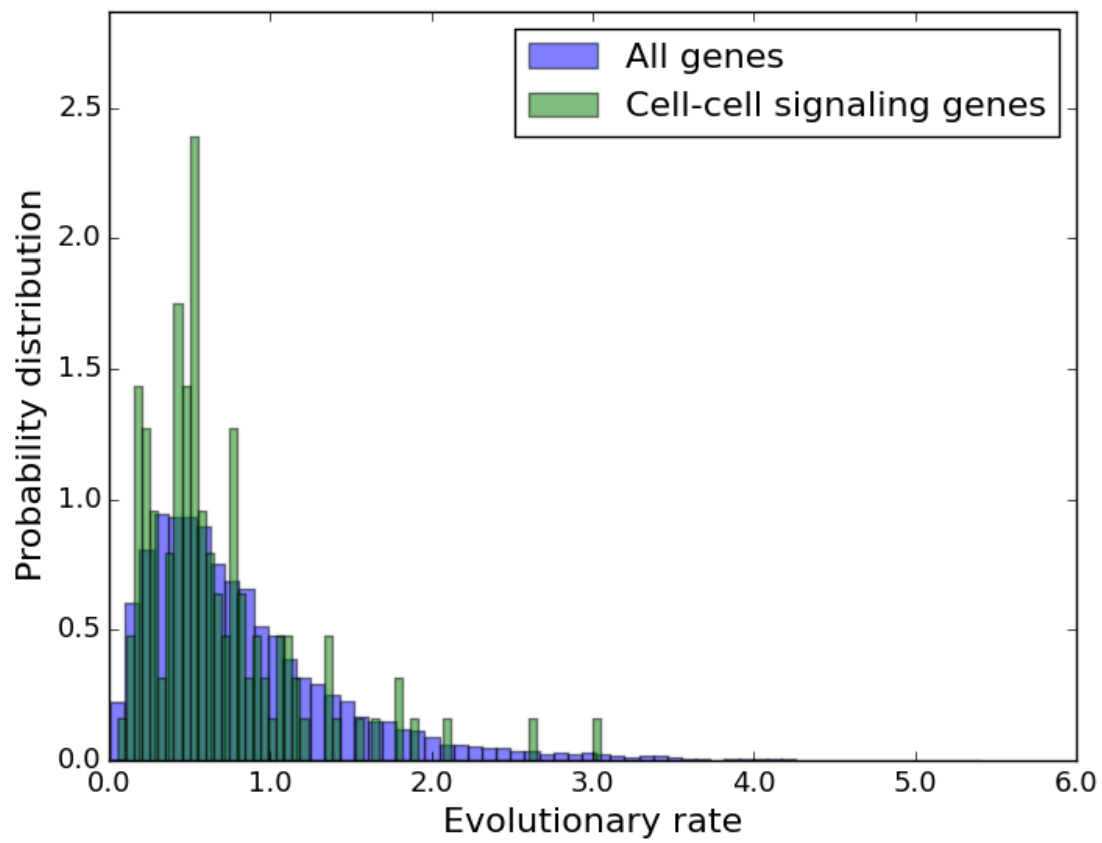

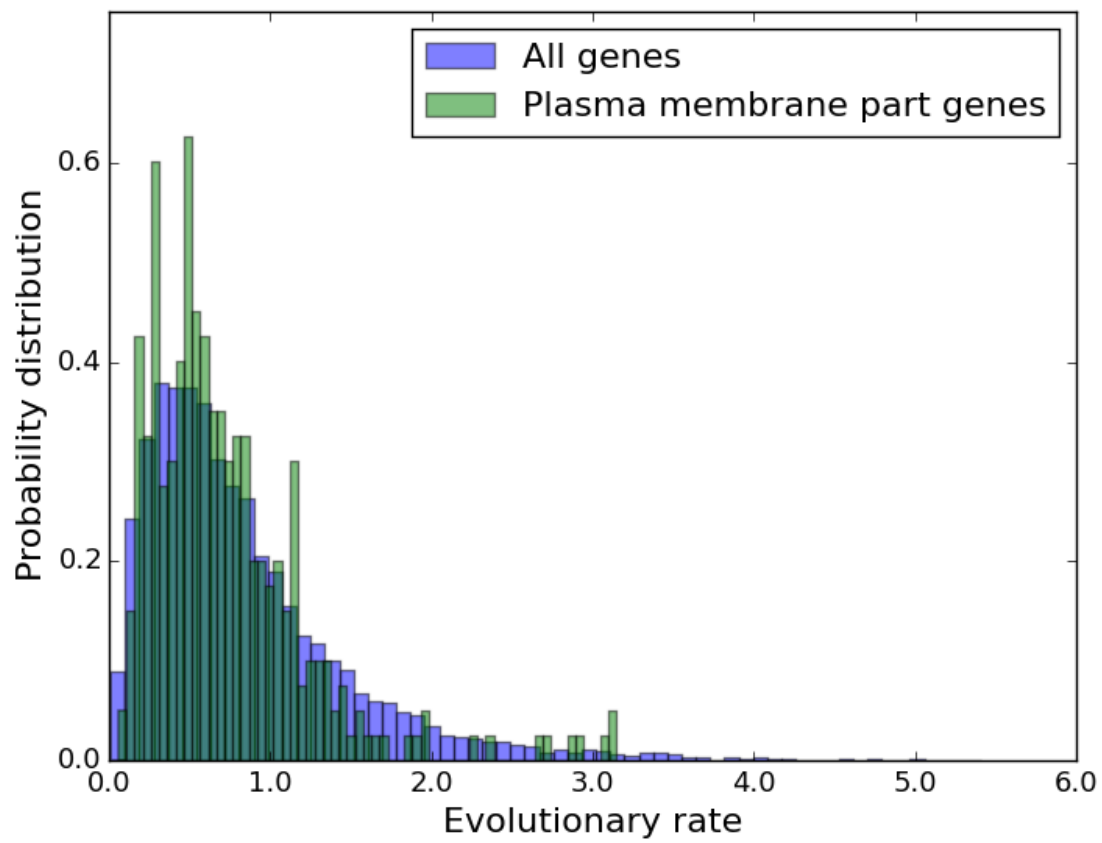

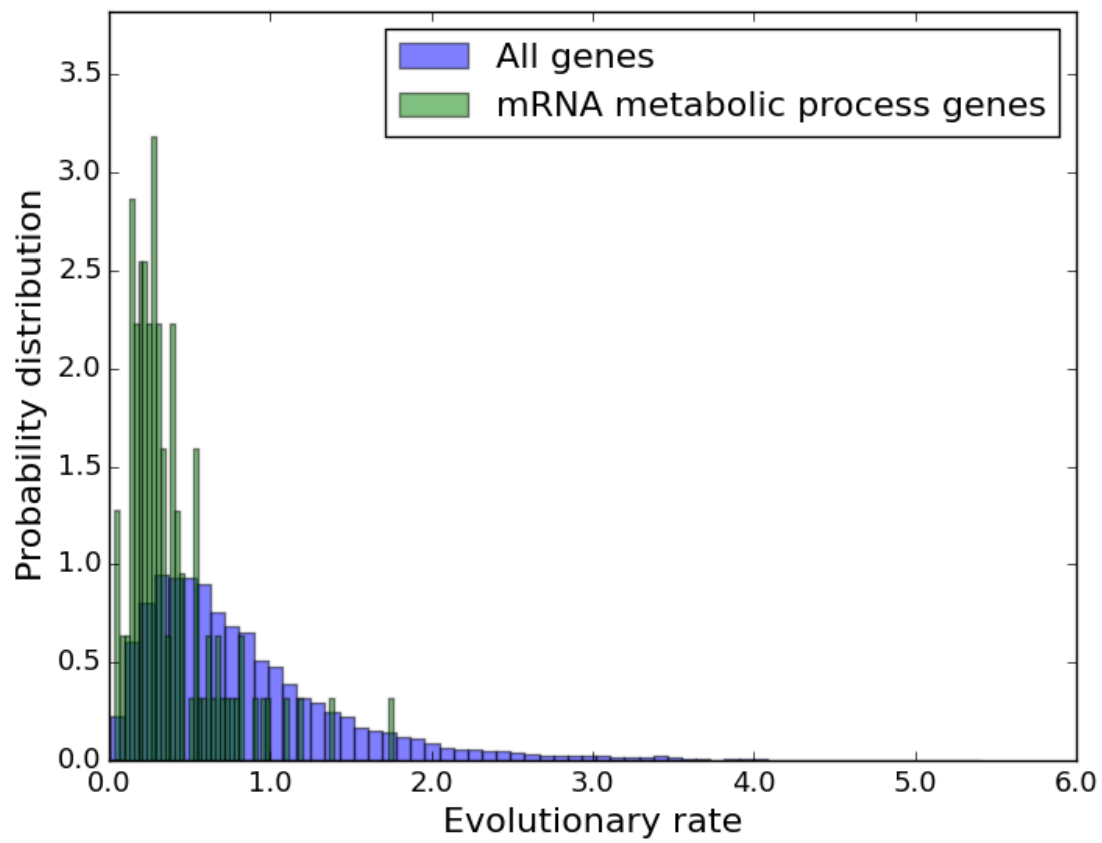

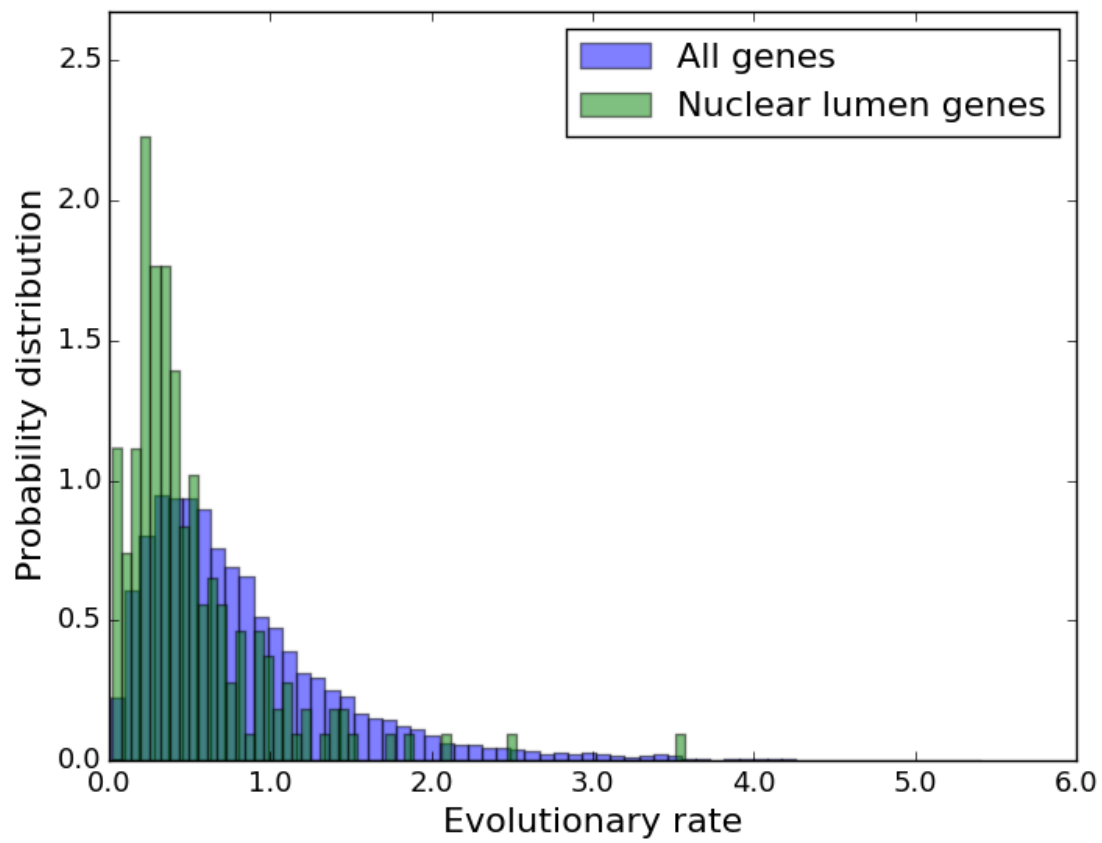

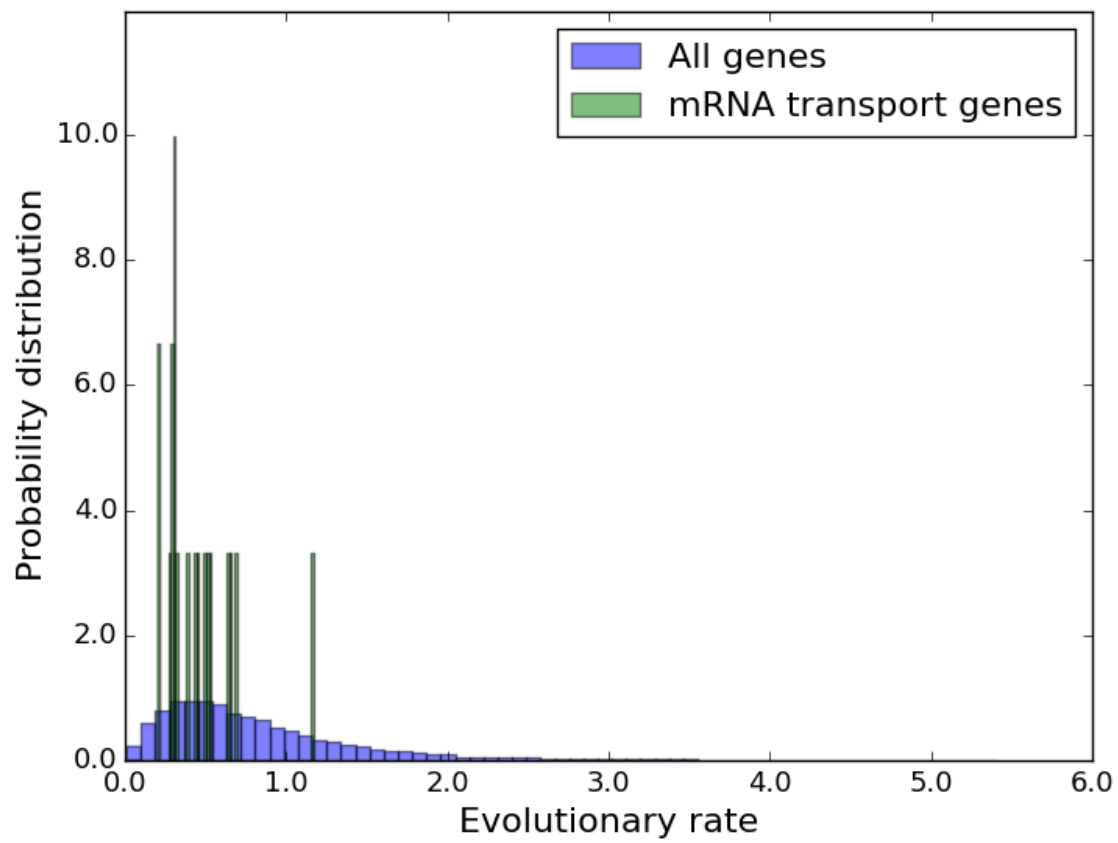

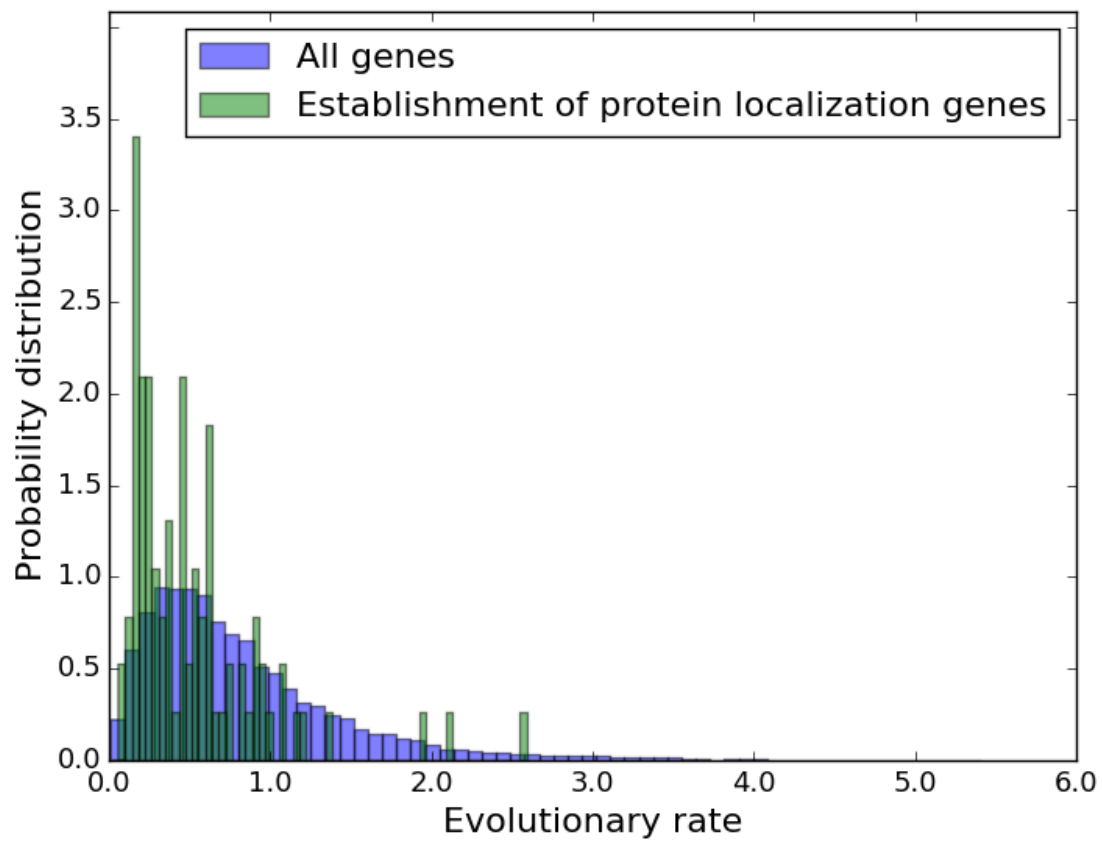

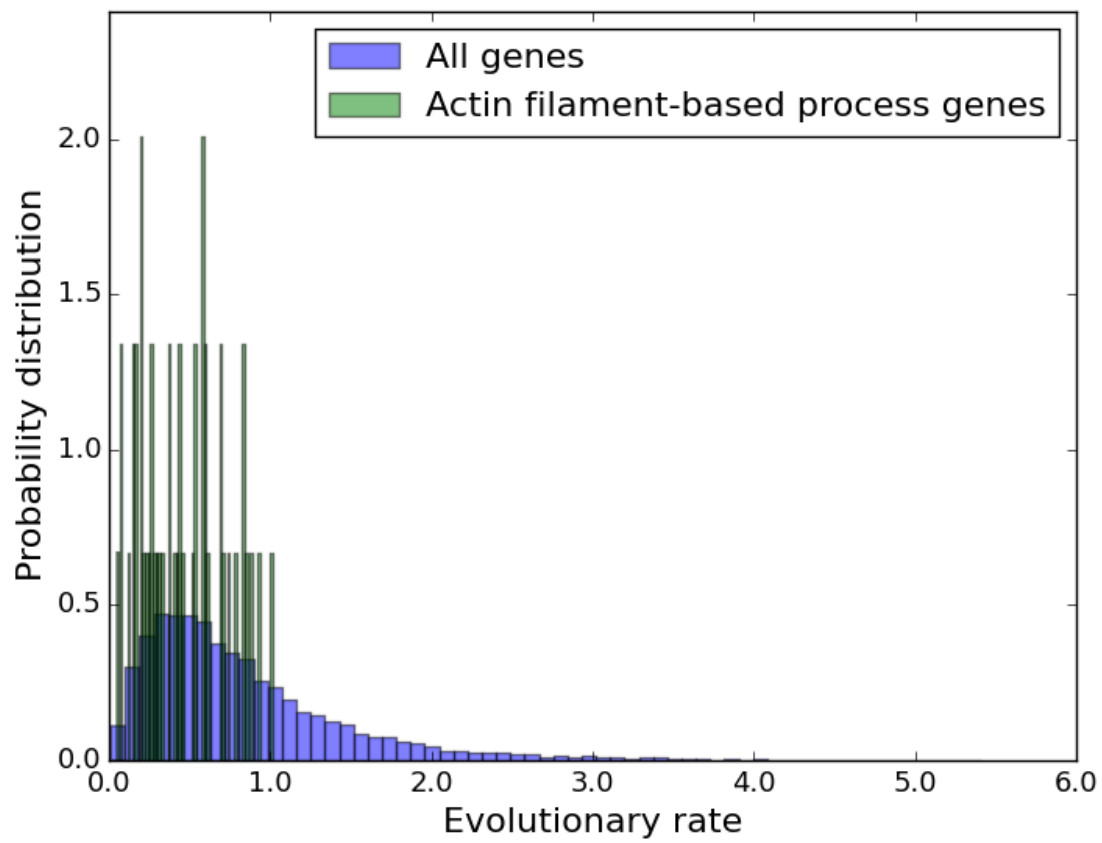

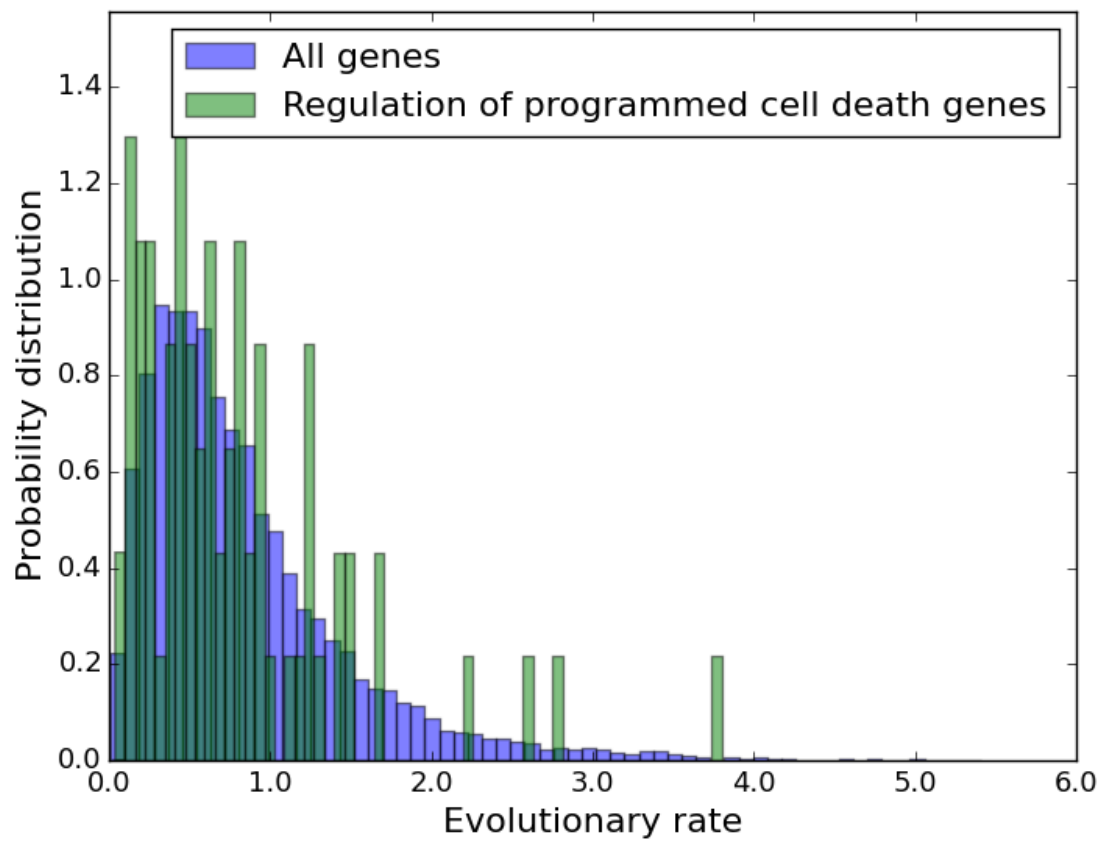

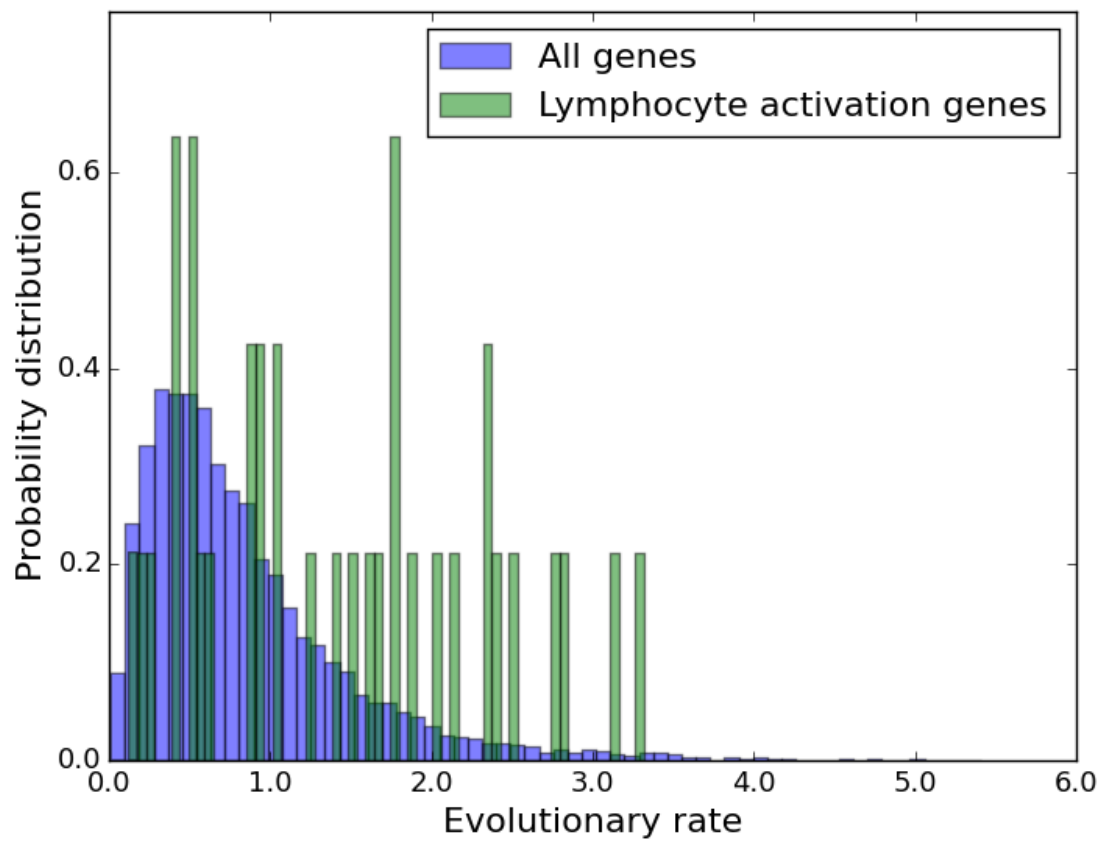

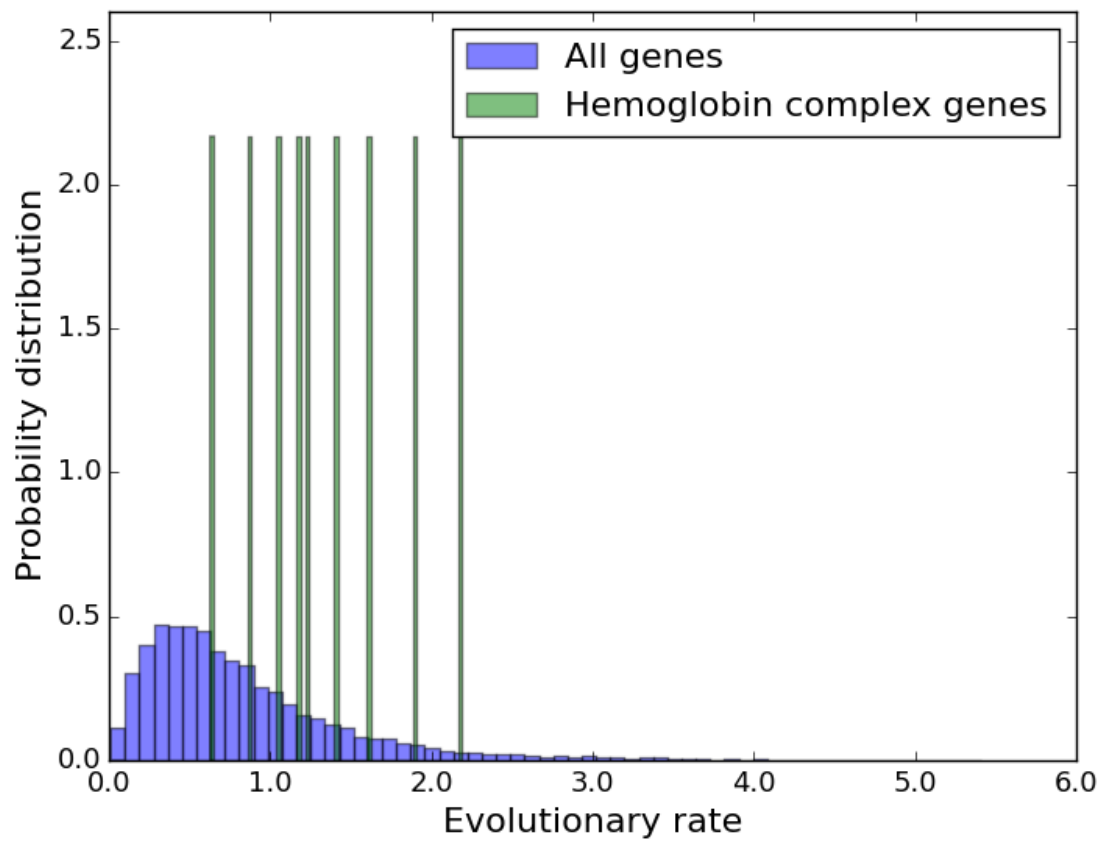

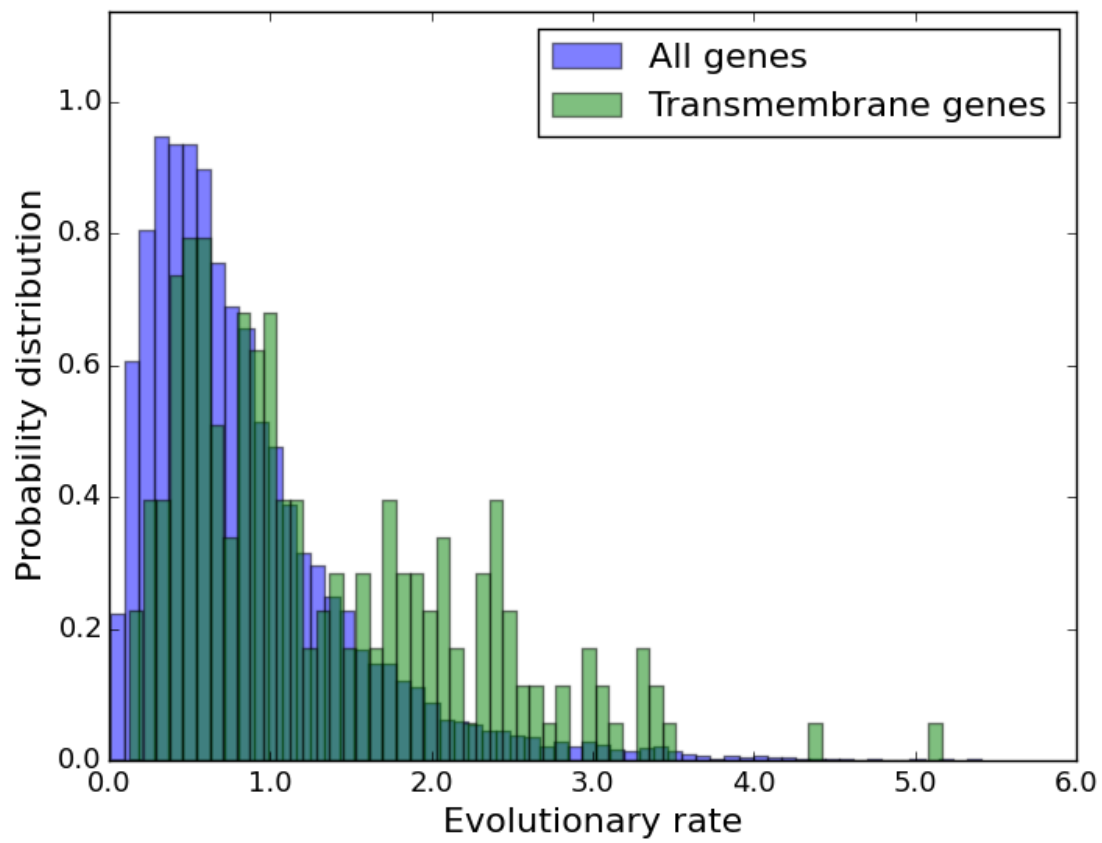

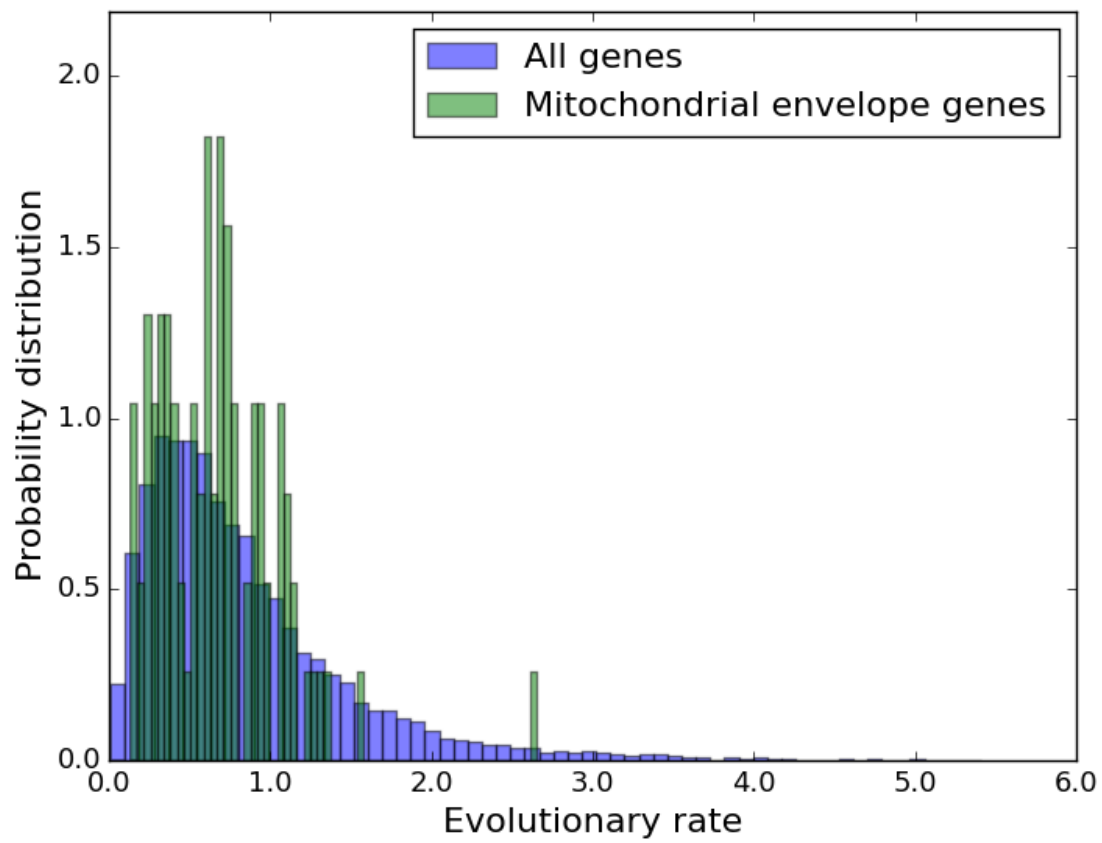

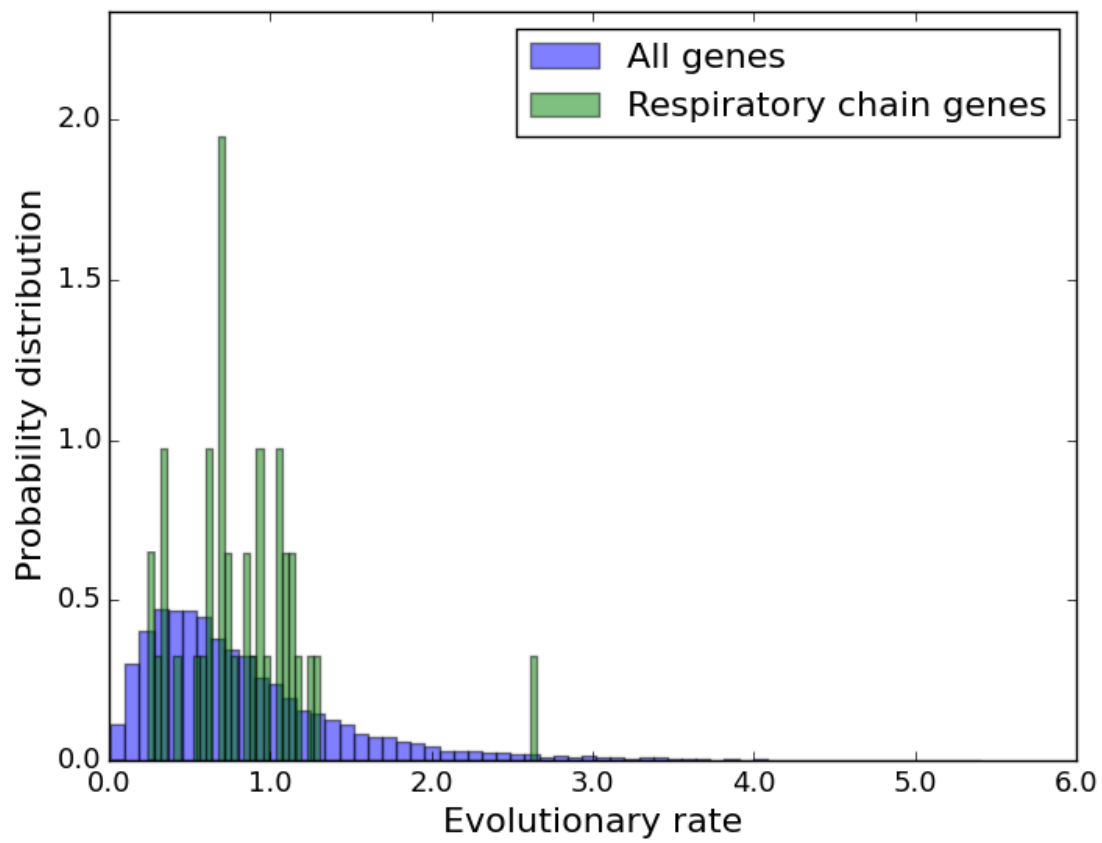

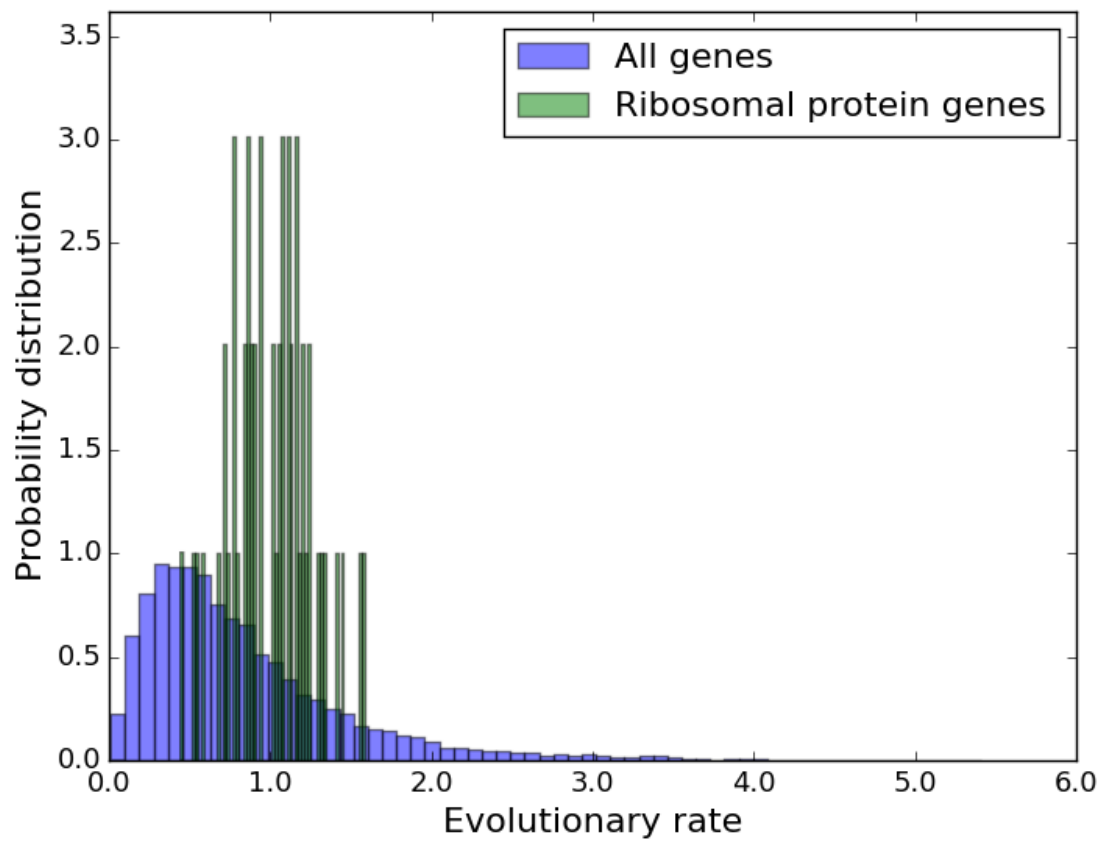

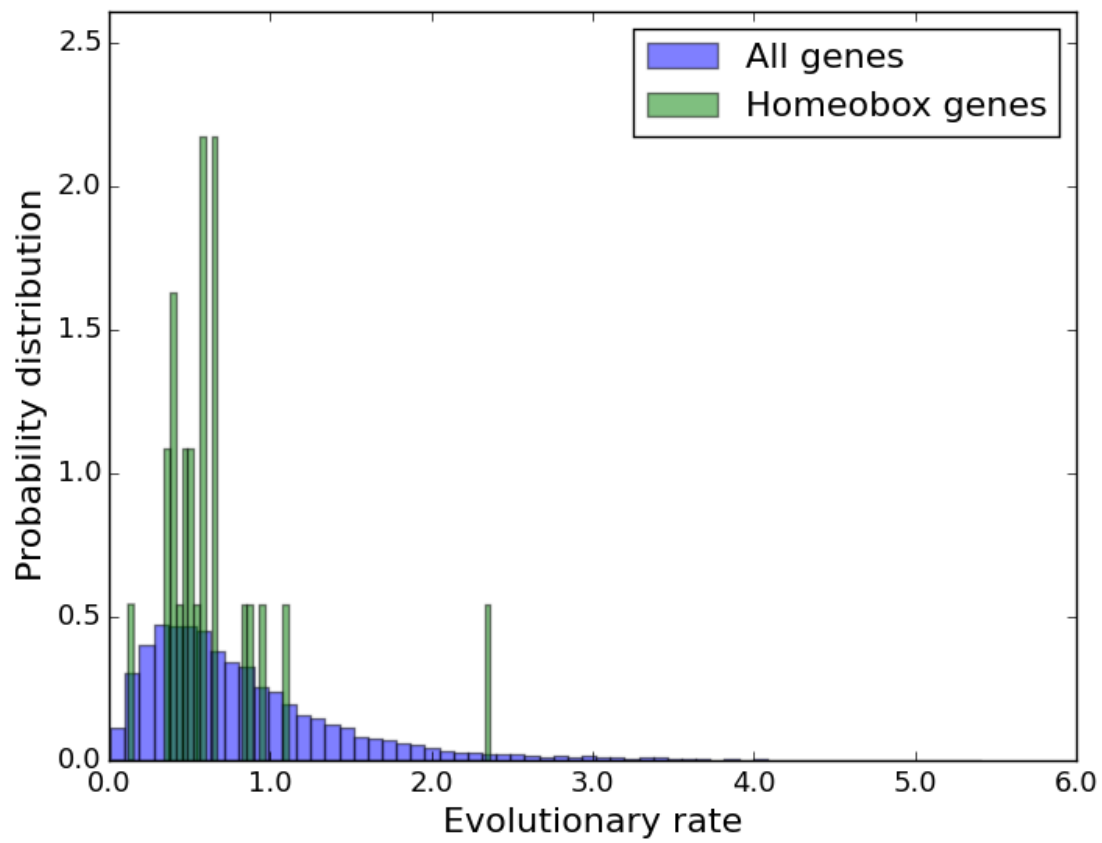

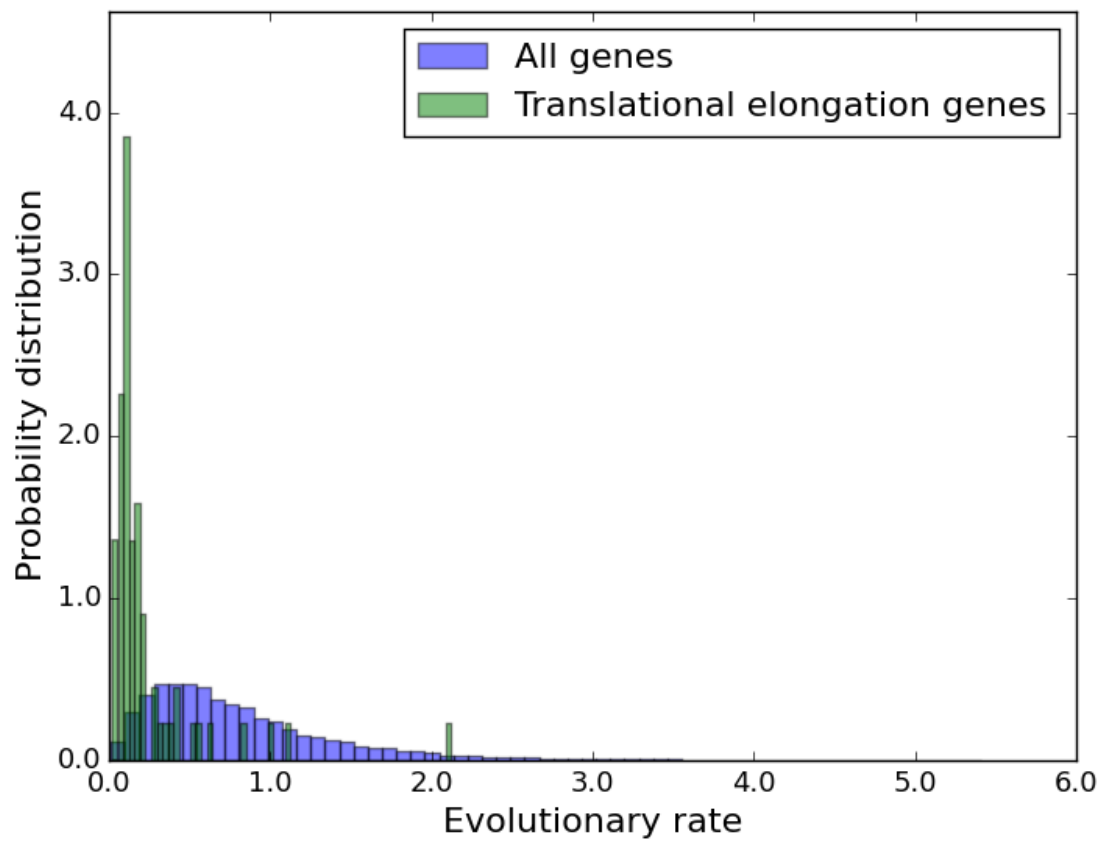

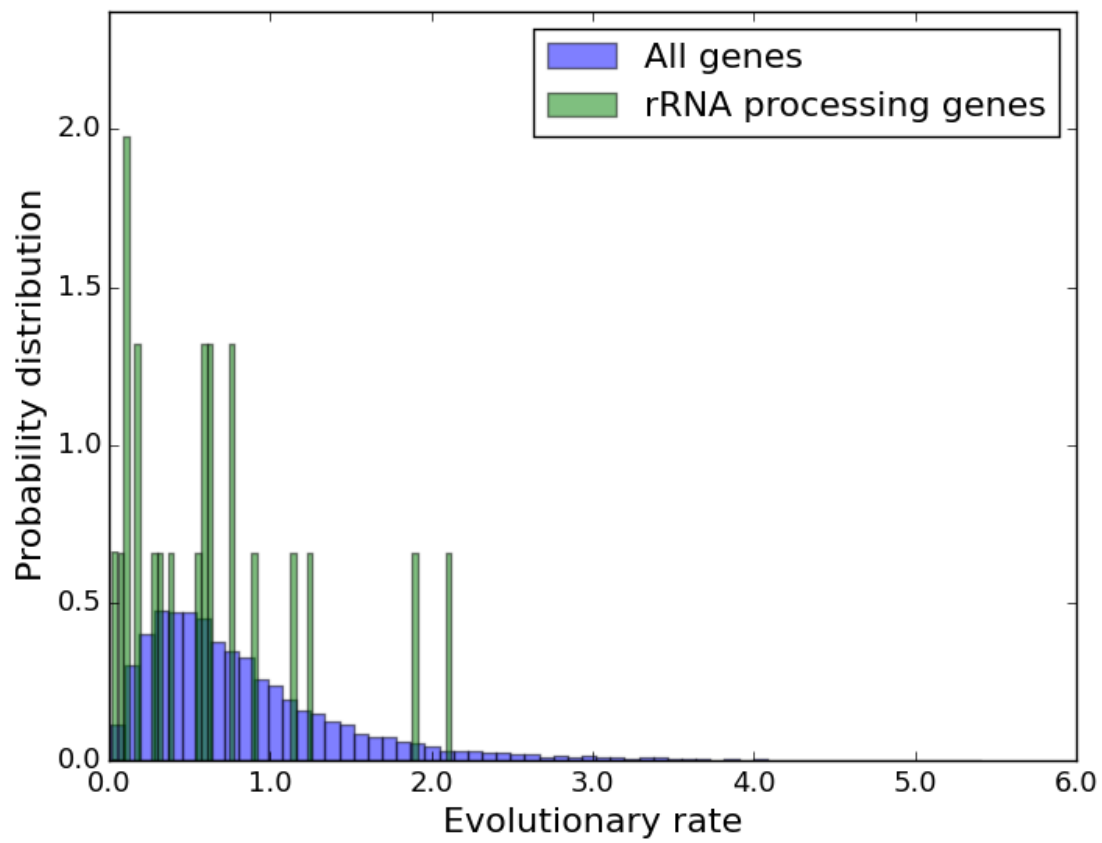

Supplement: S7 Fig — These figures show the distribution of evolutionary rates for all communities and DAVID groups reported in Table 1. (PDF) [file pcbi.1005009.s007.pdf]

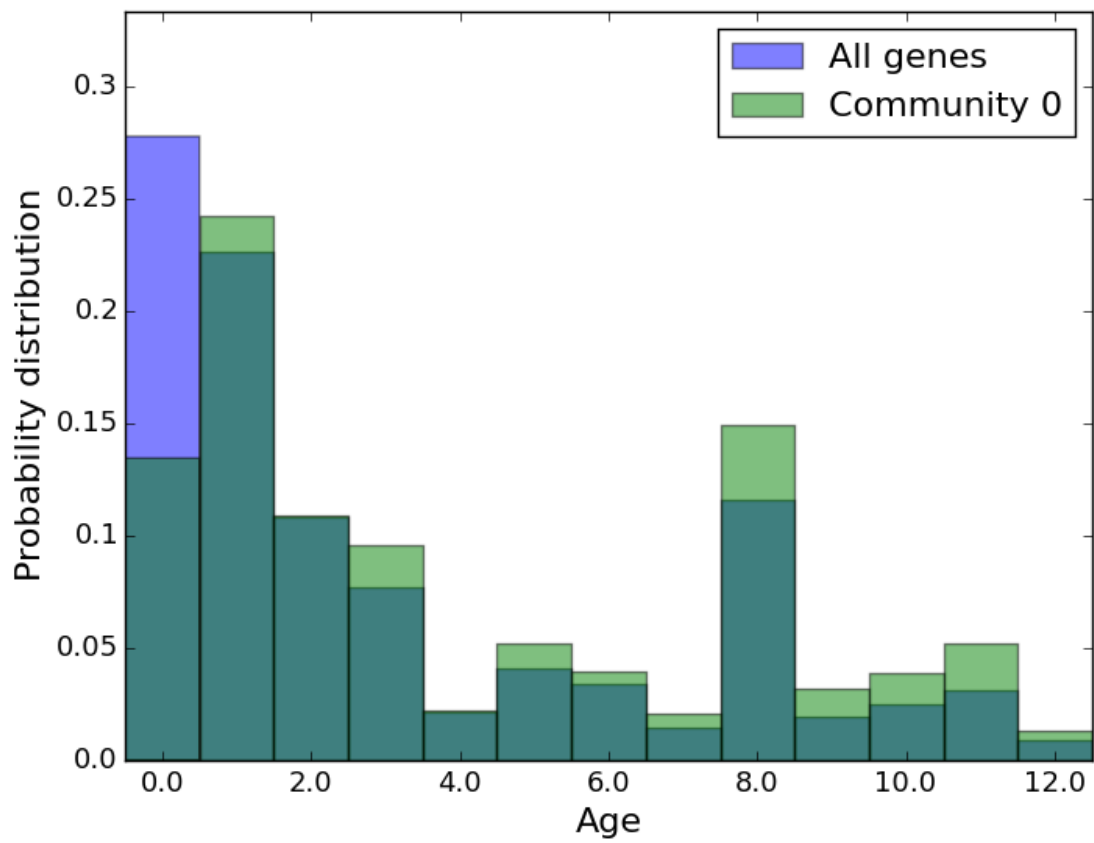

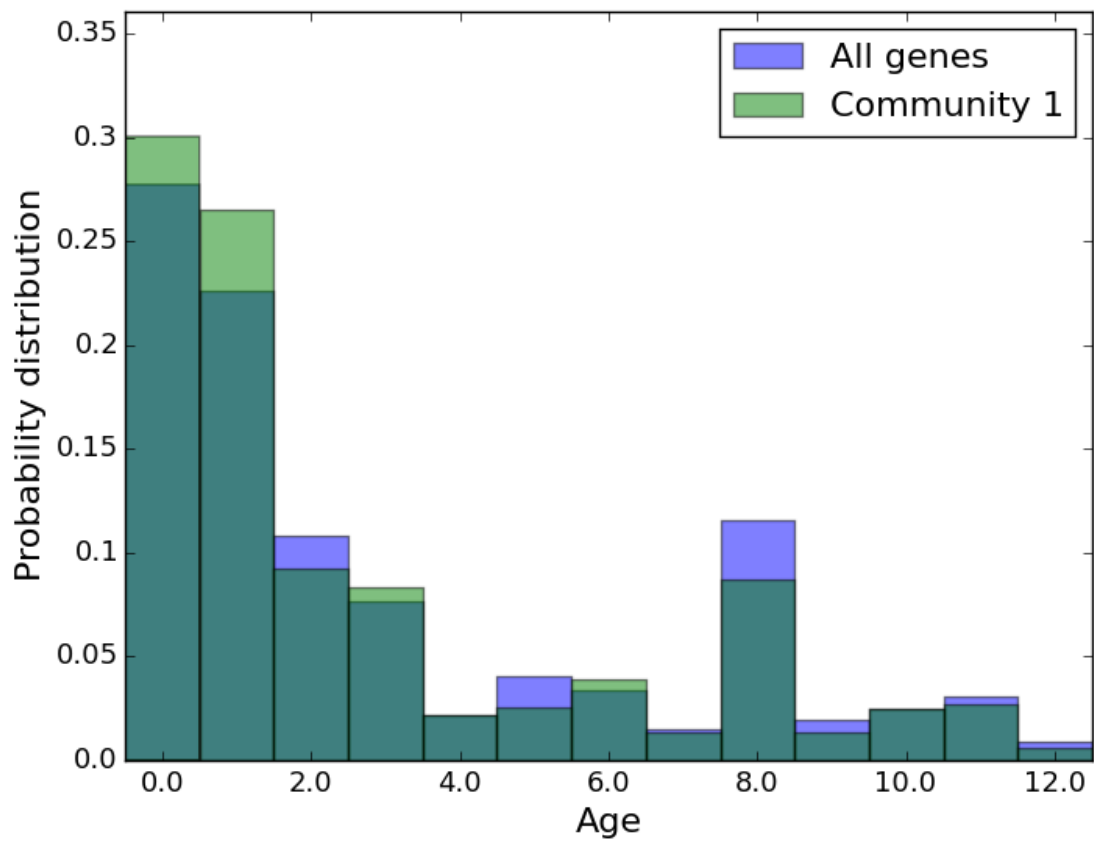

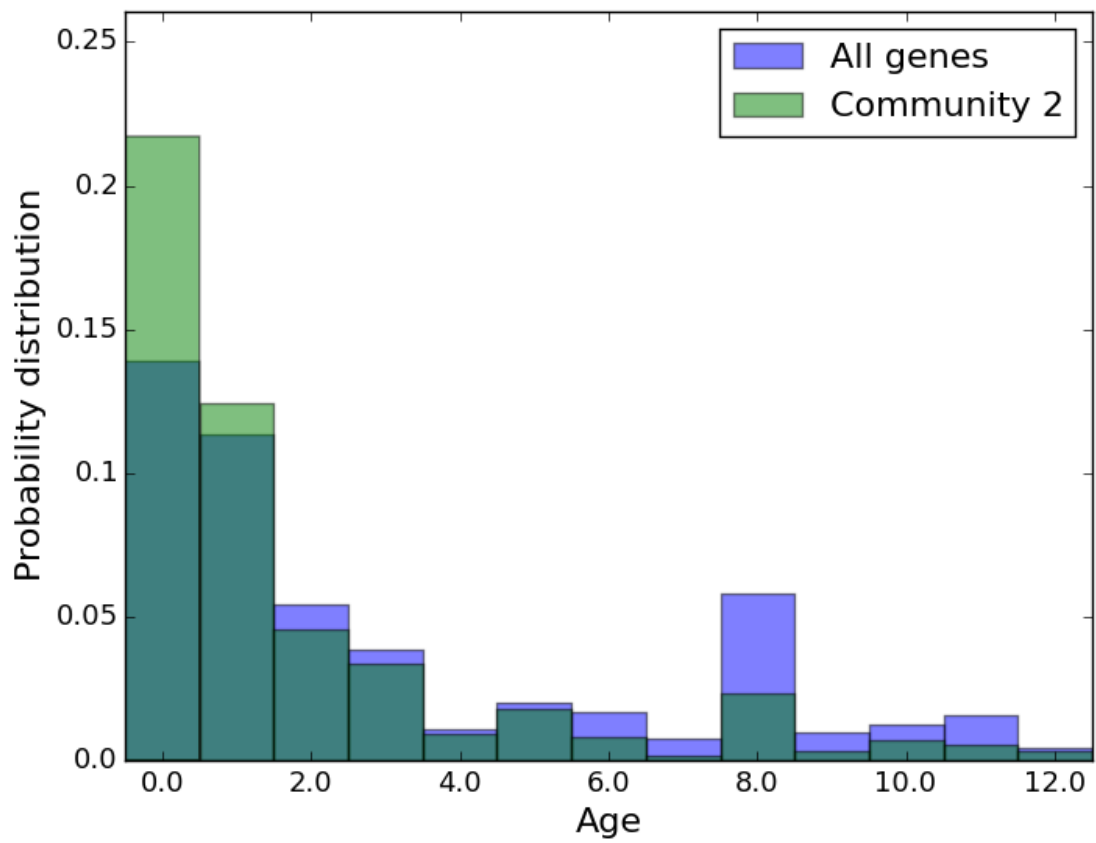

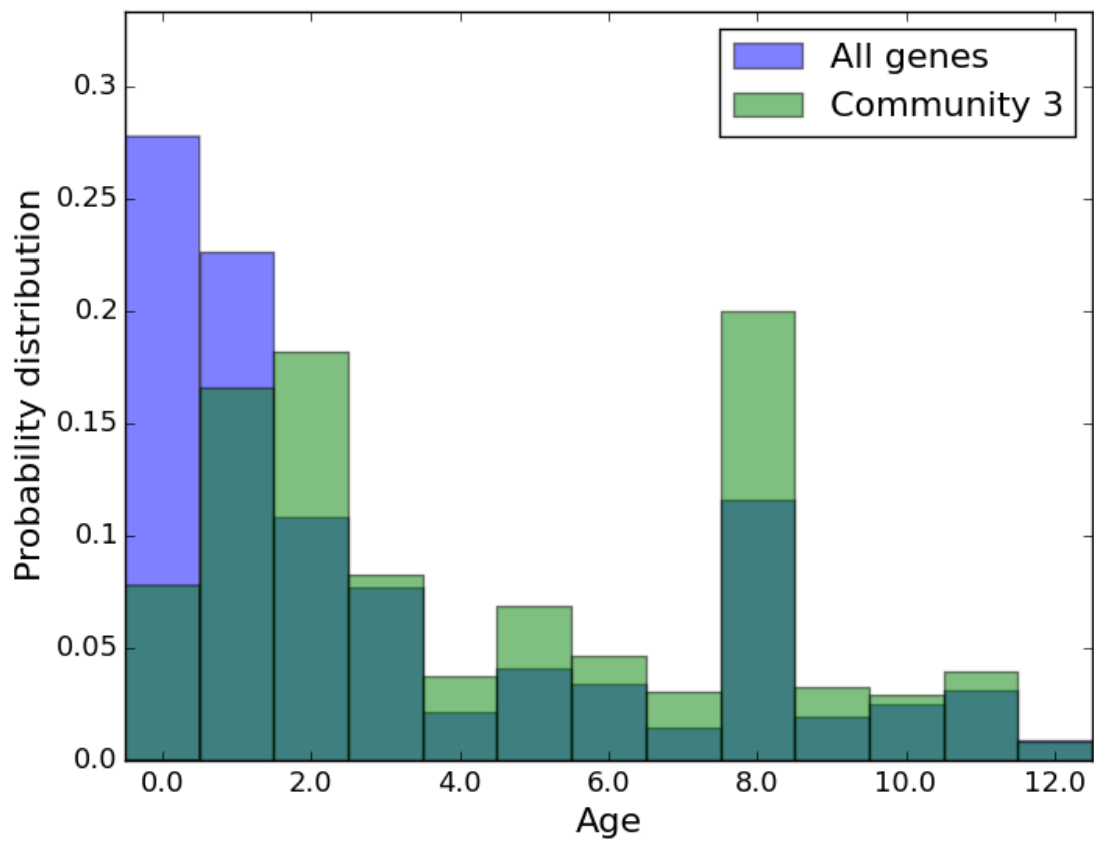

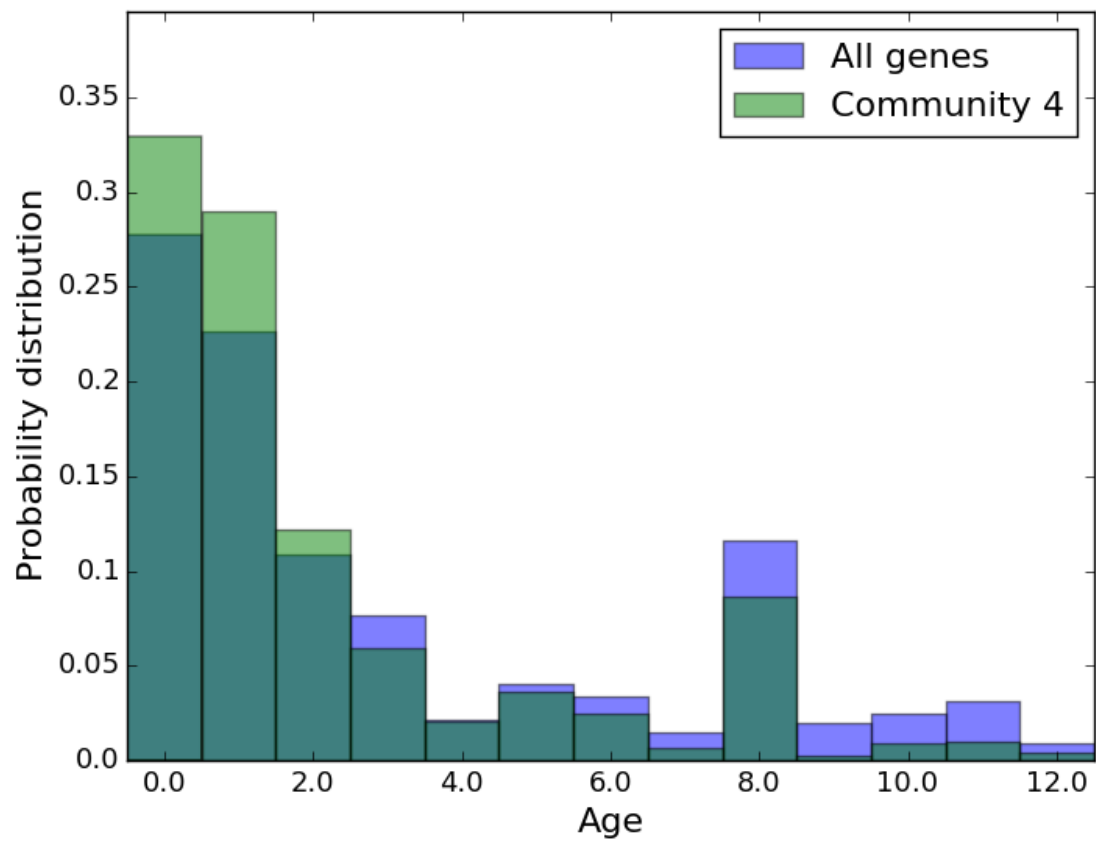

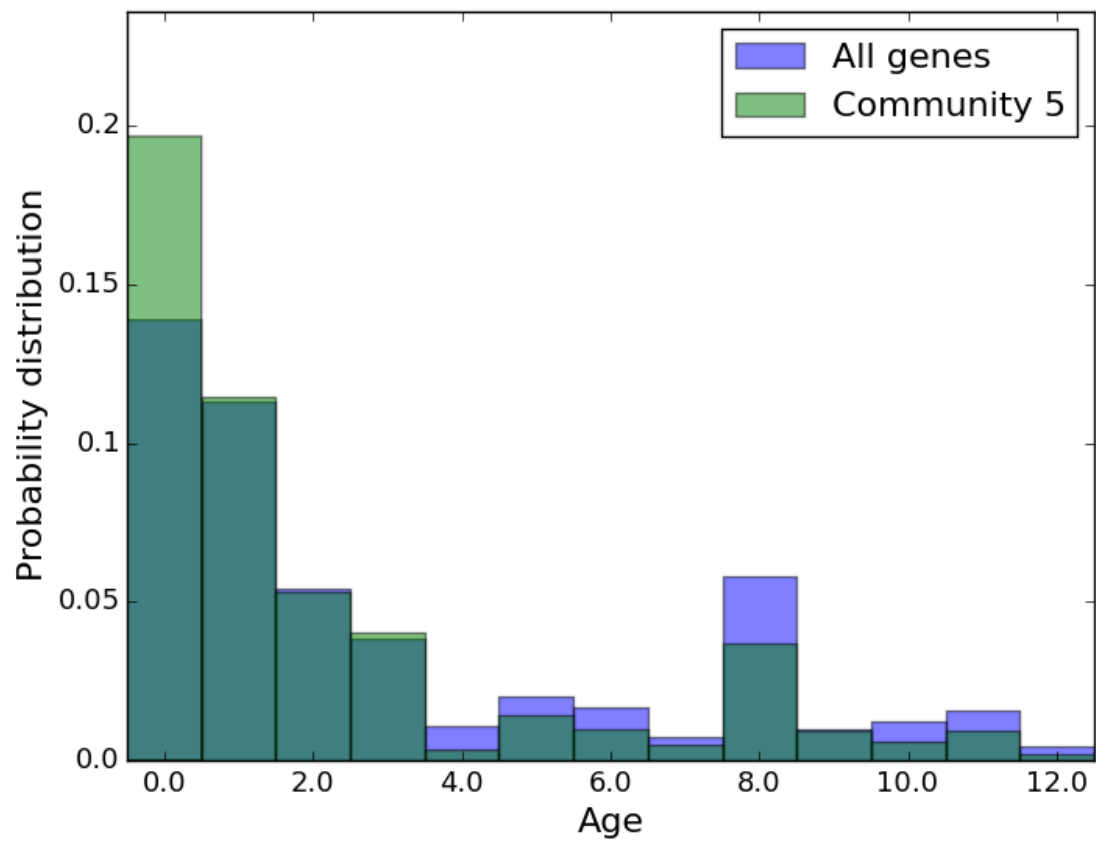

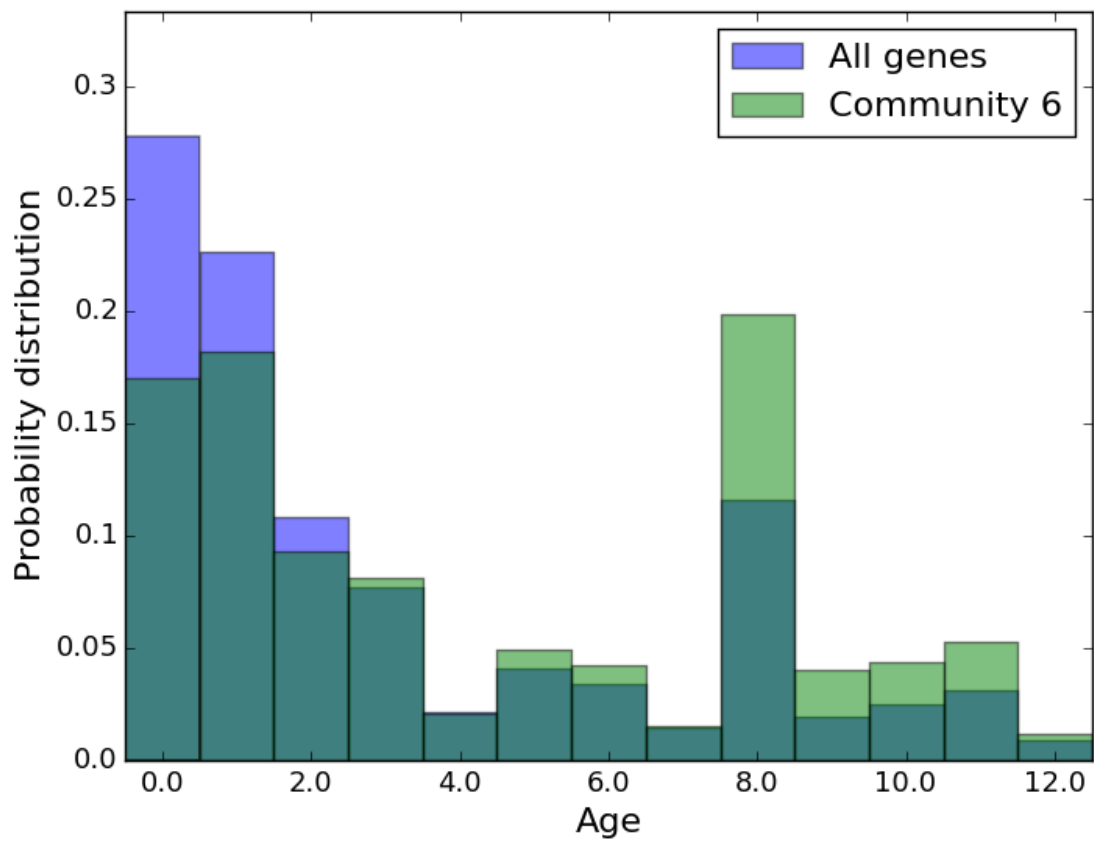

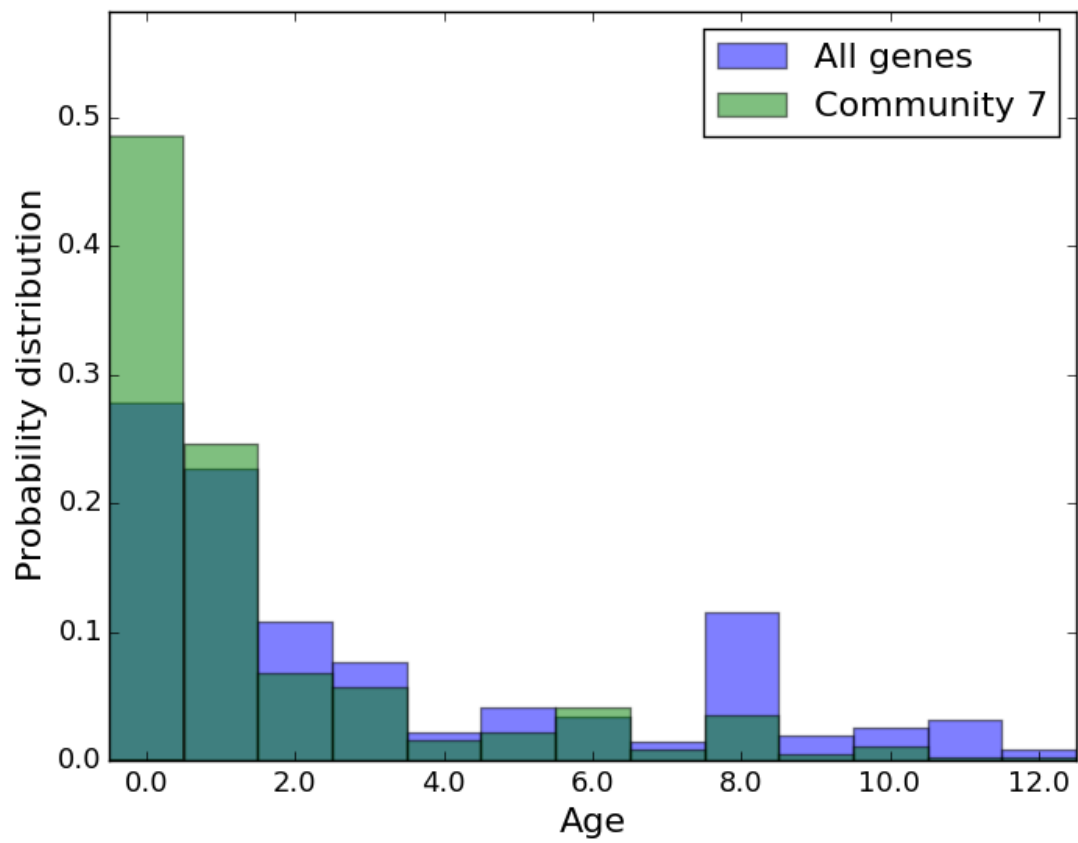

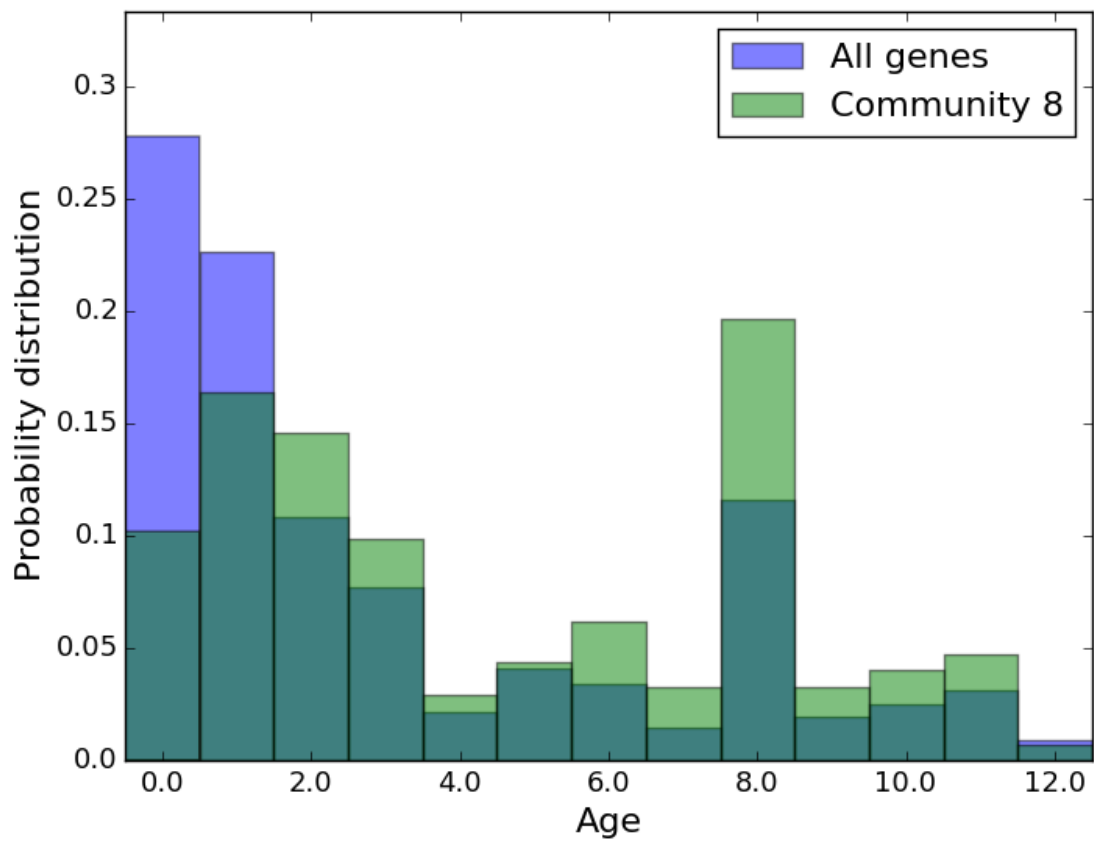

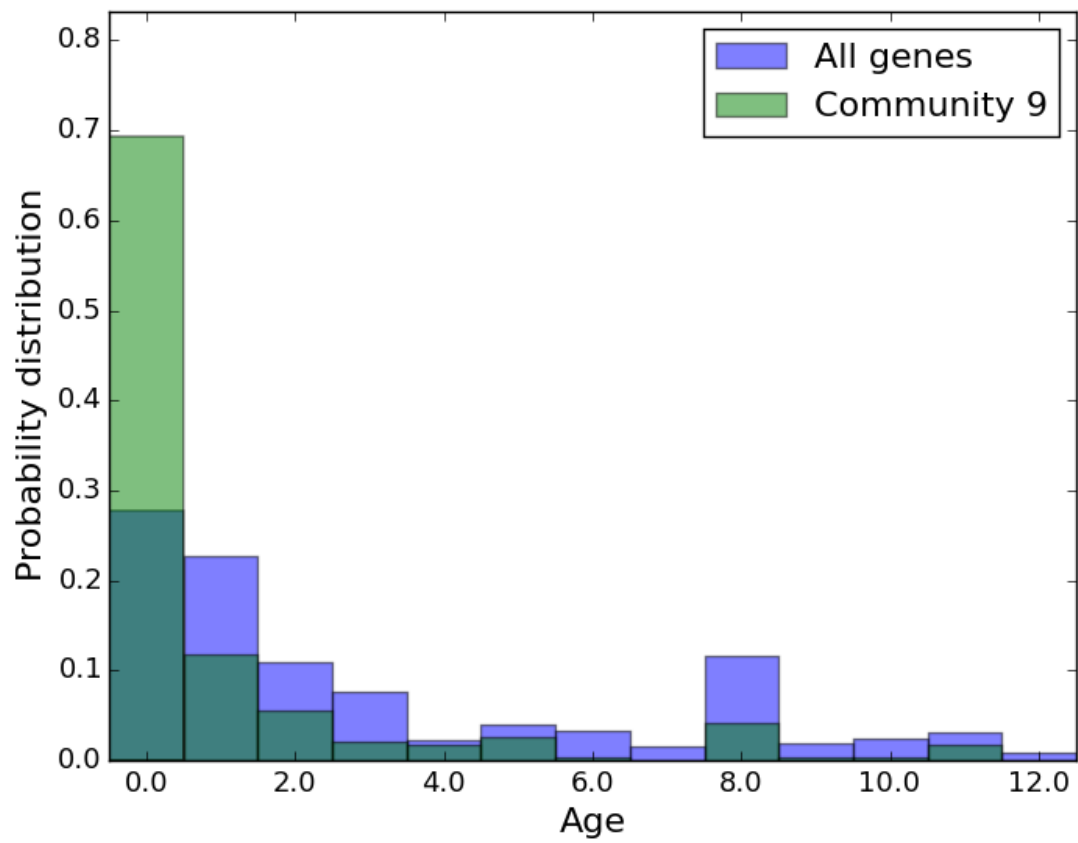

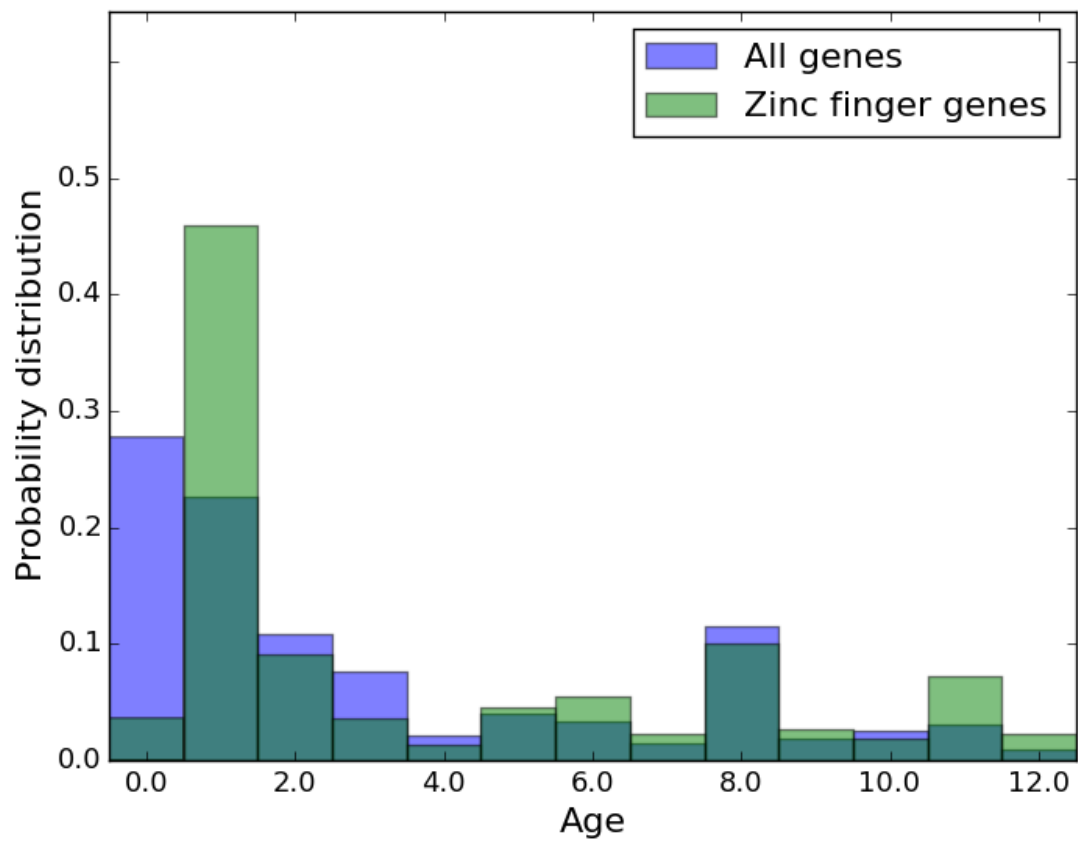

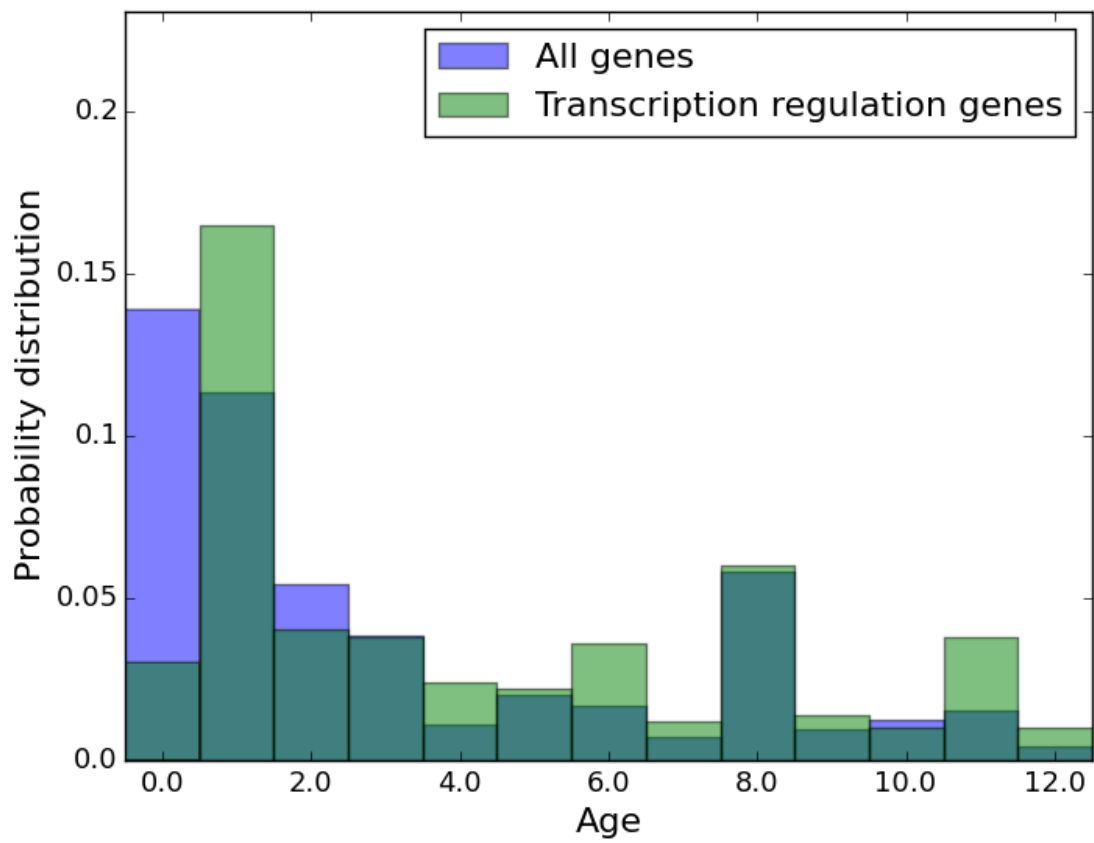

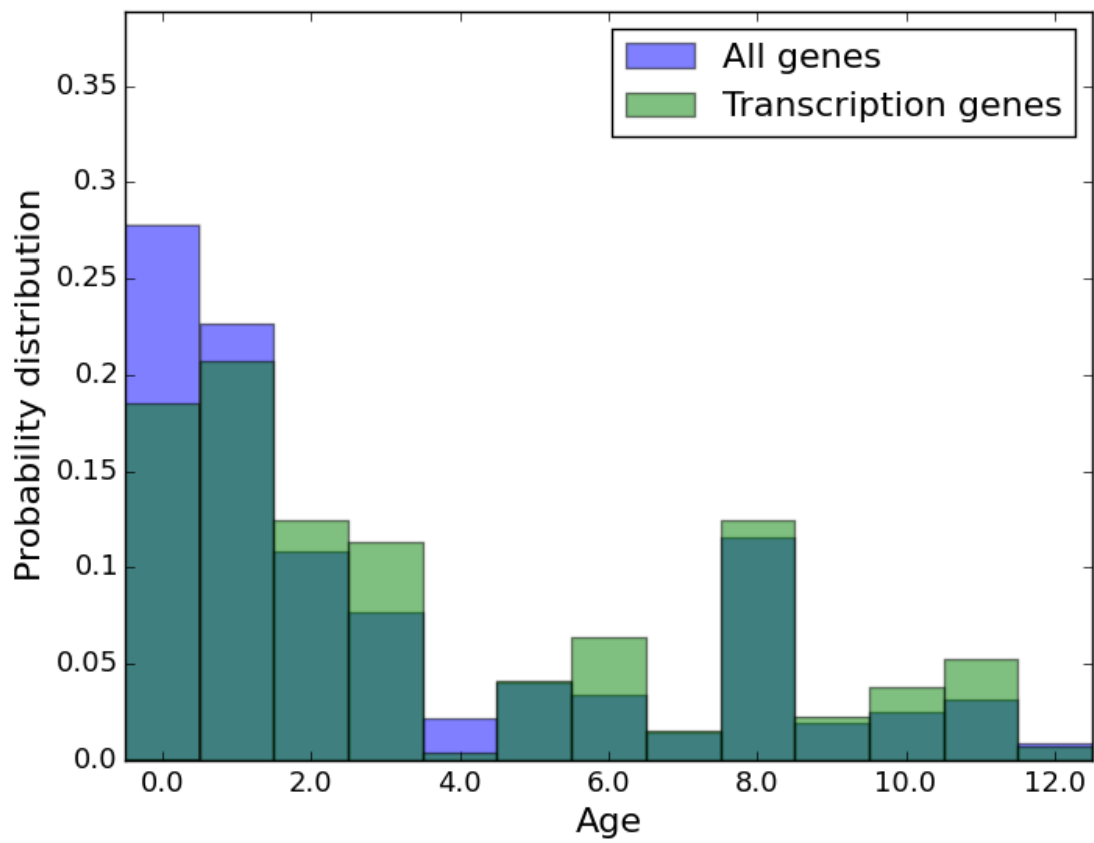

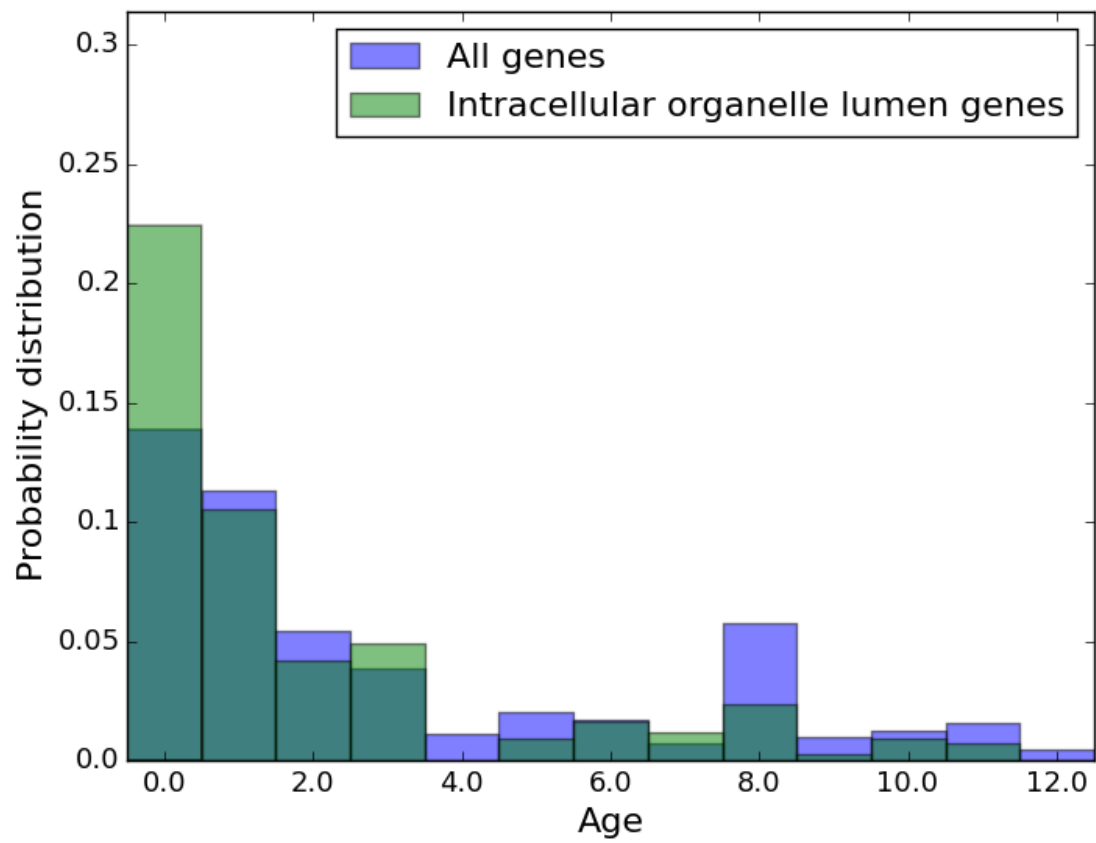

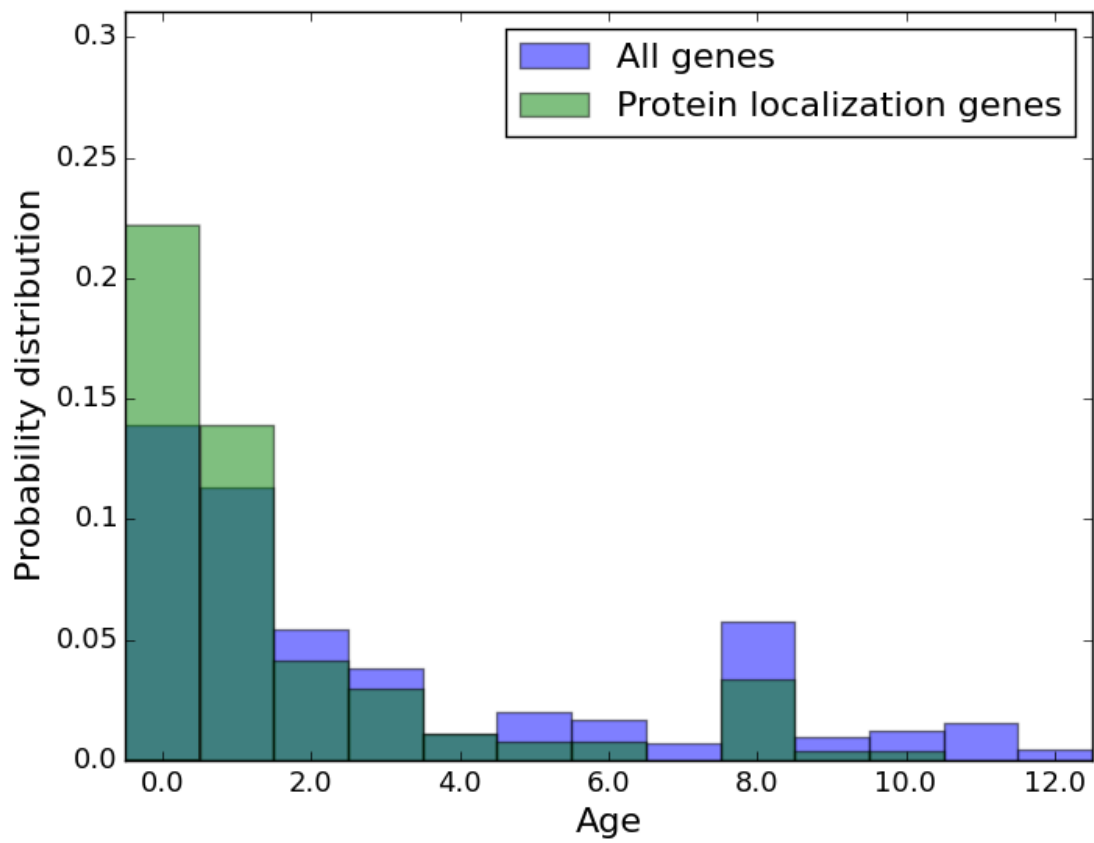

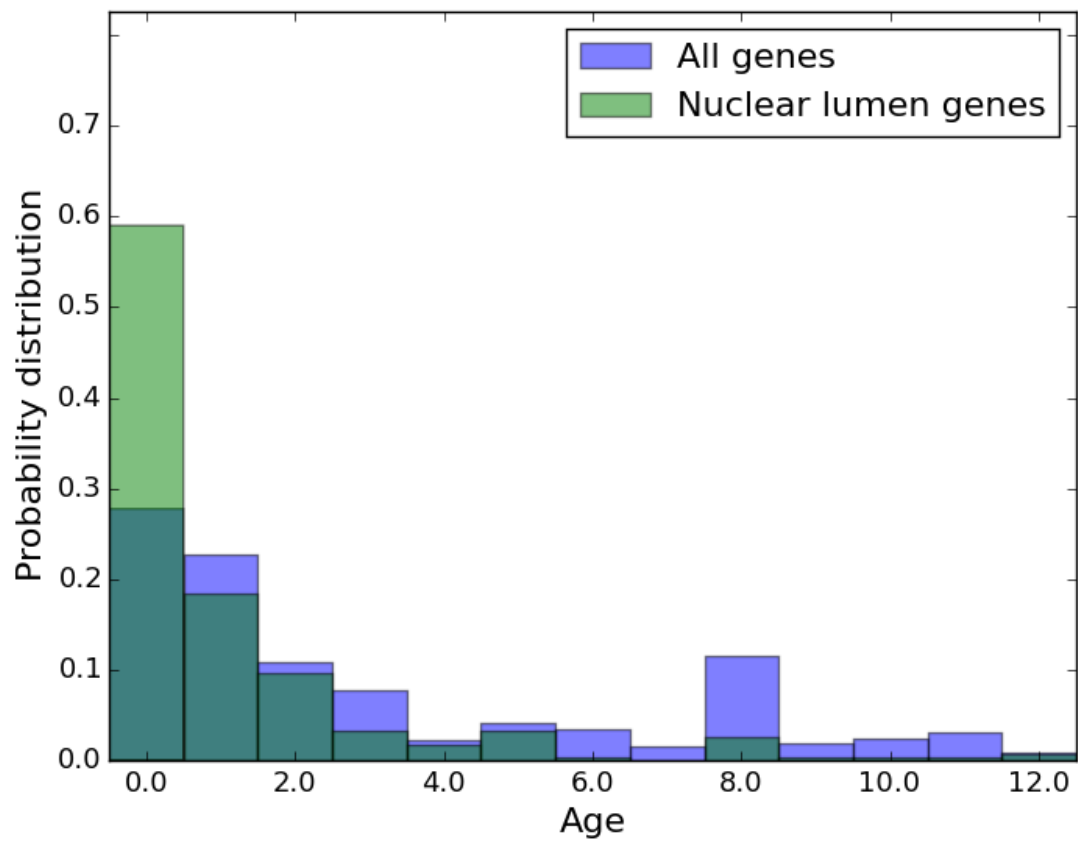

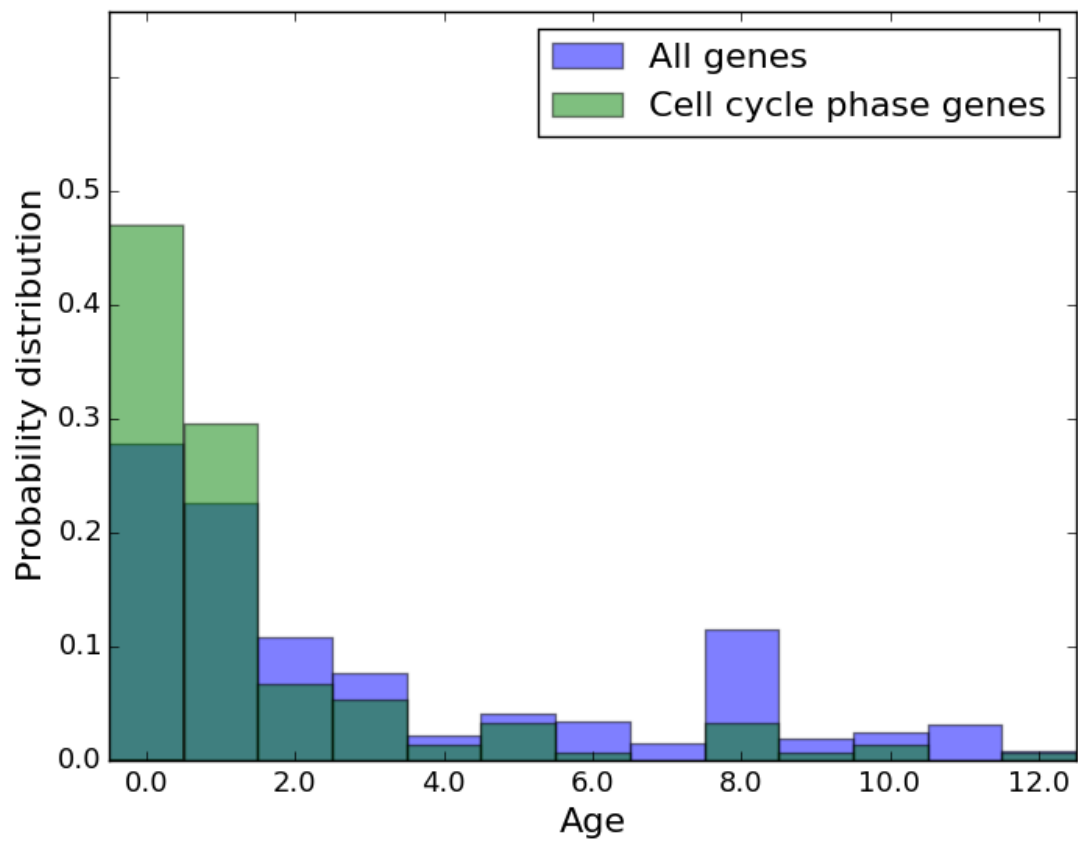

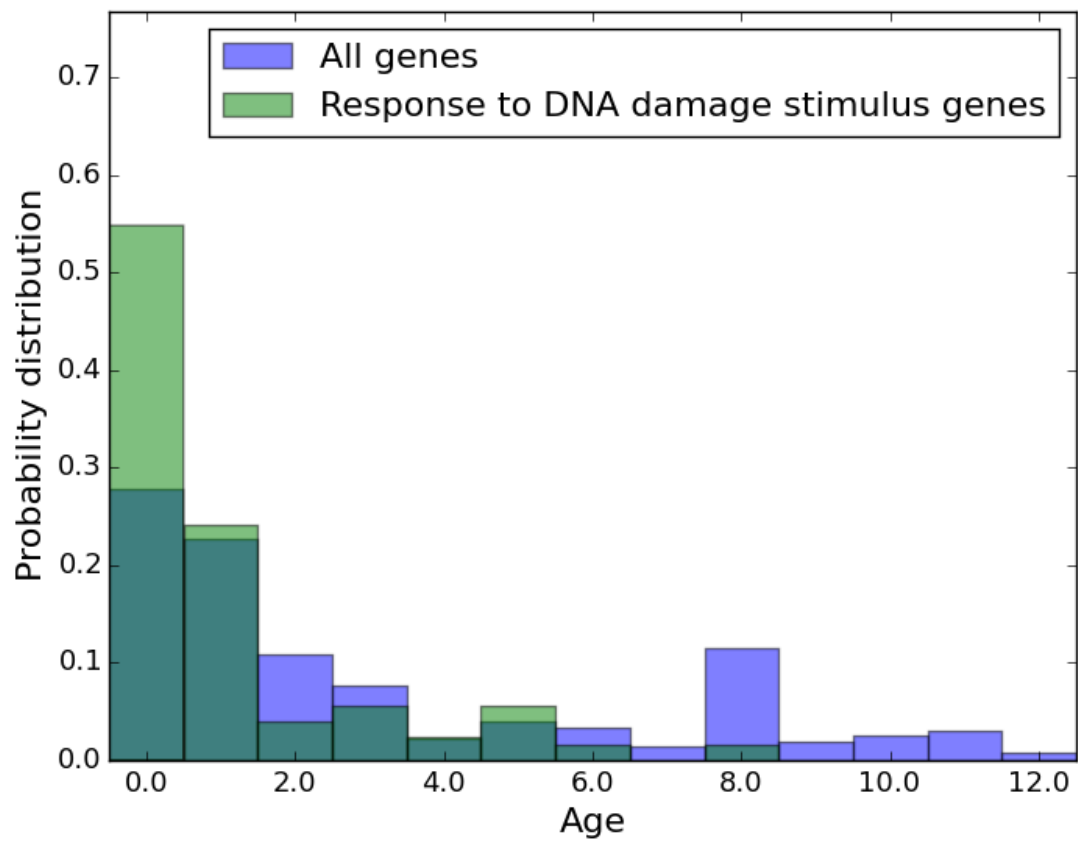

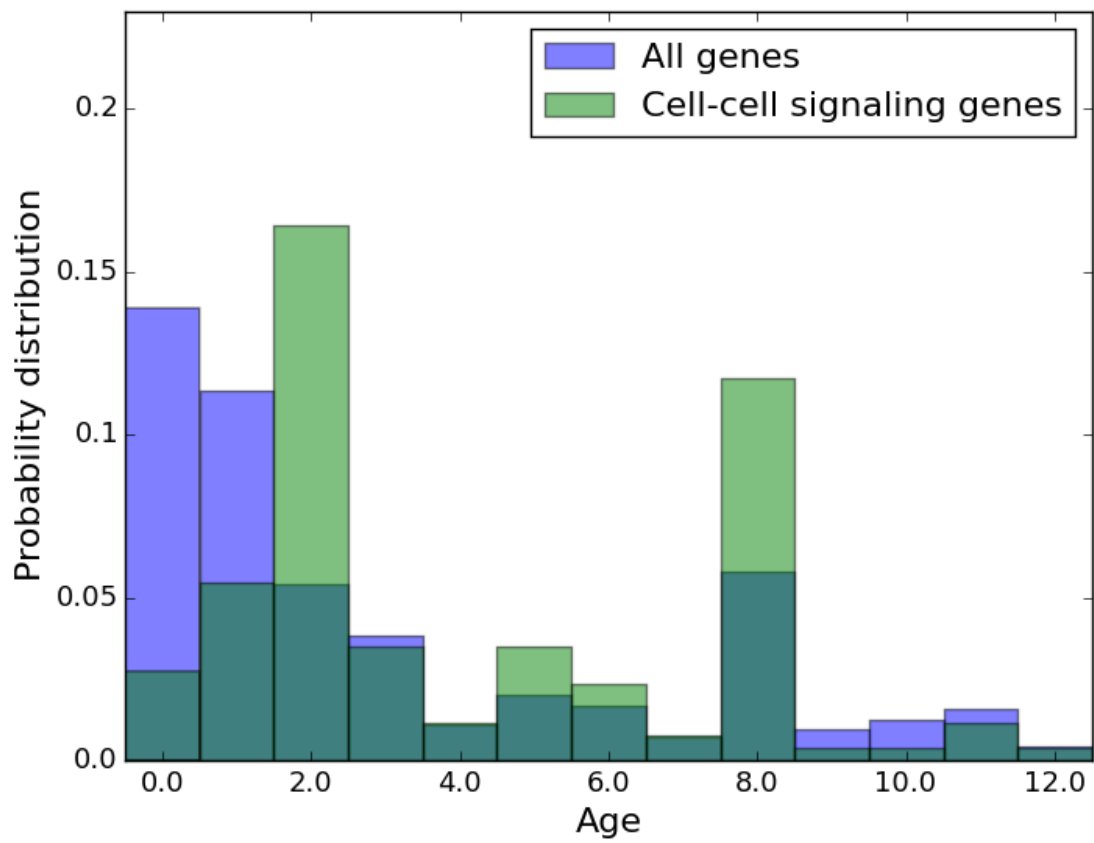

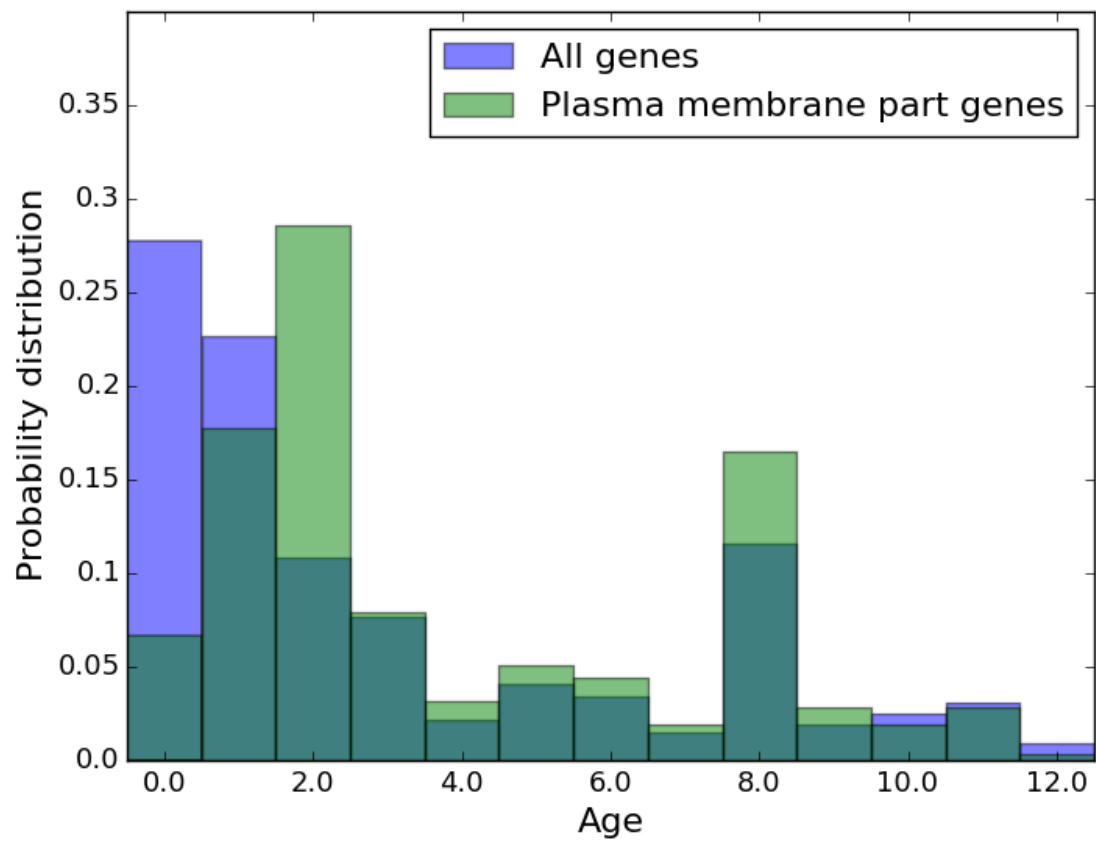

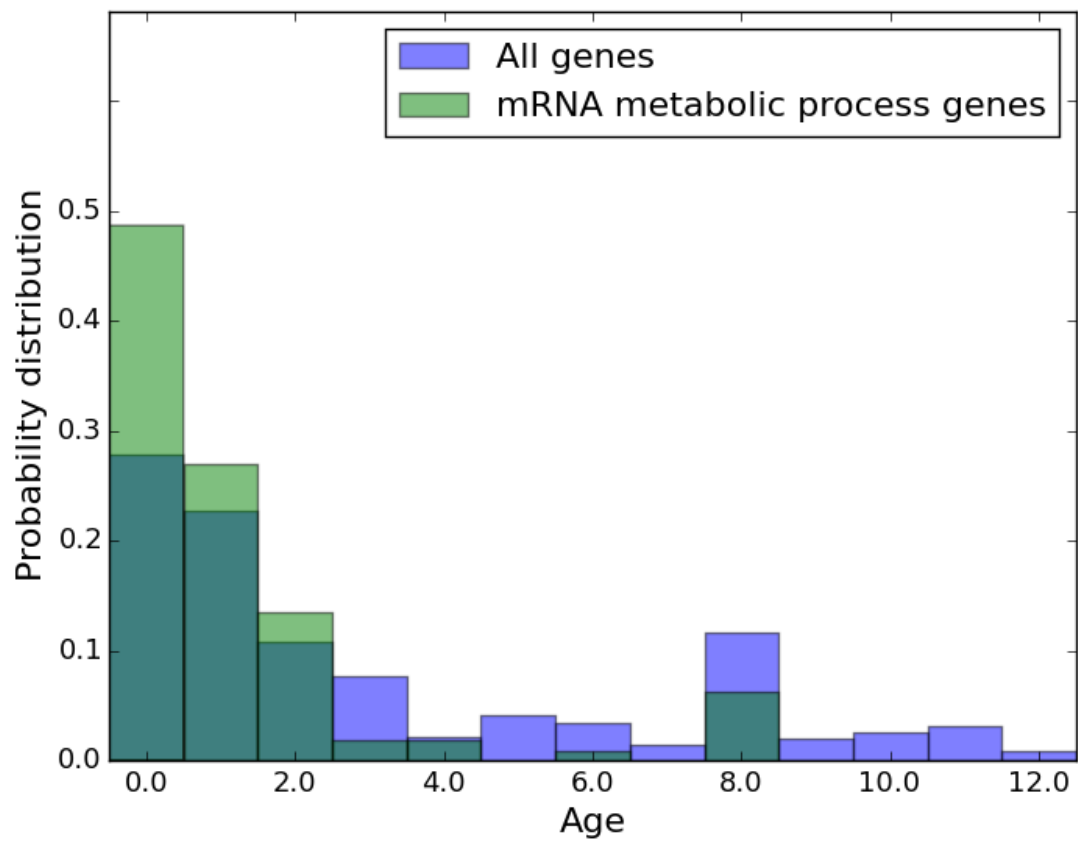

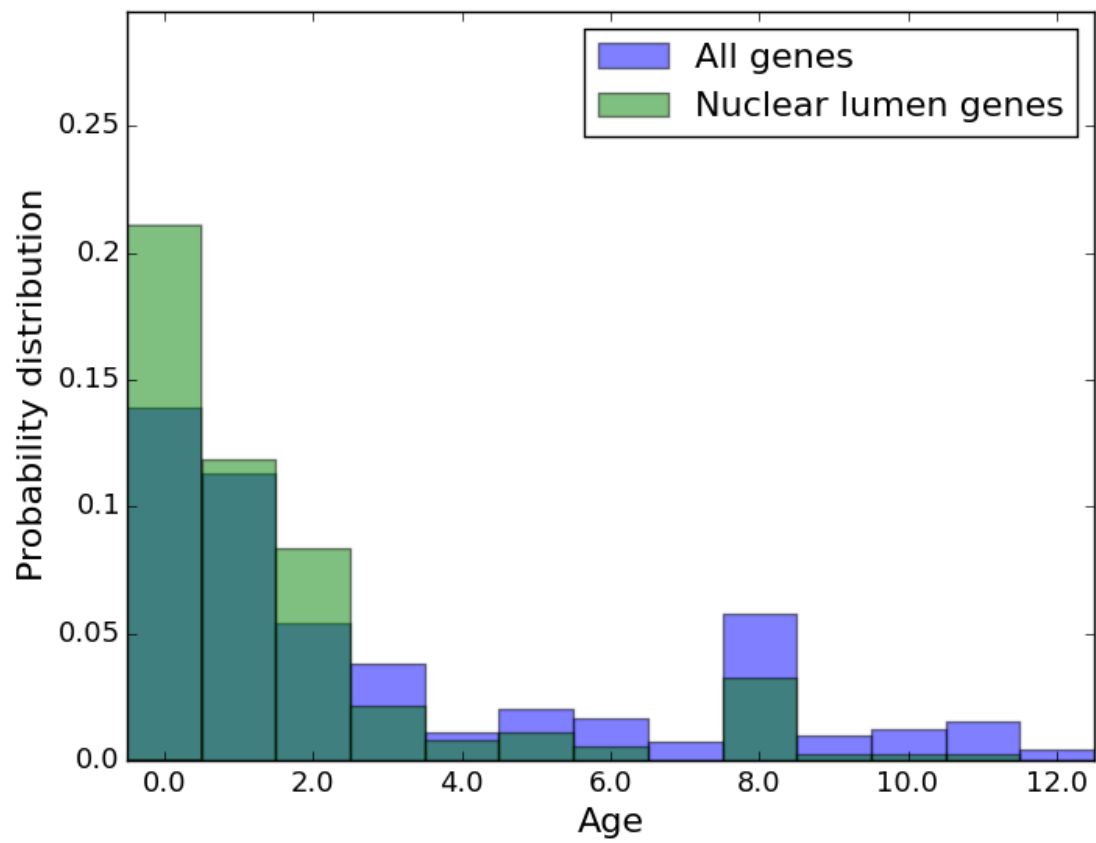

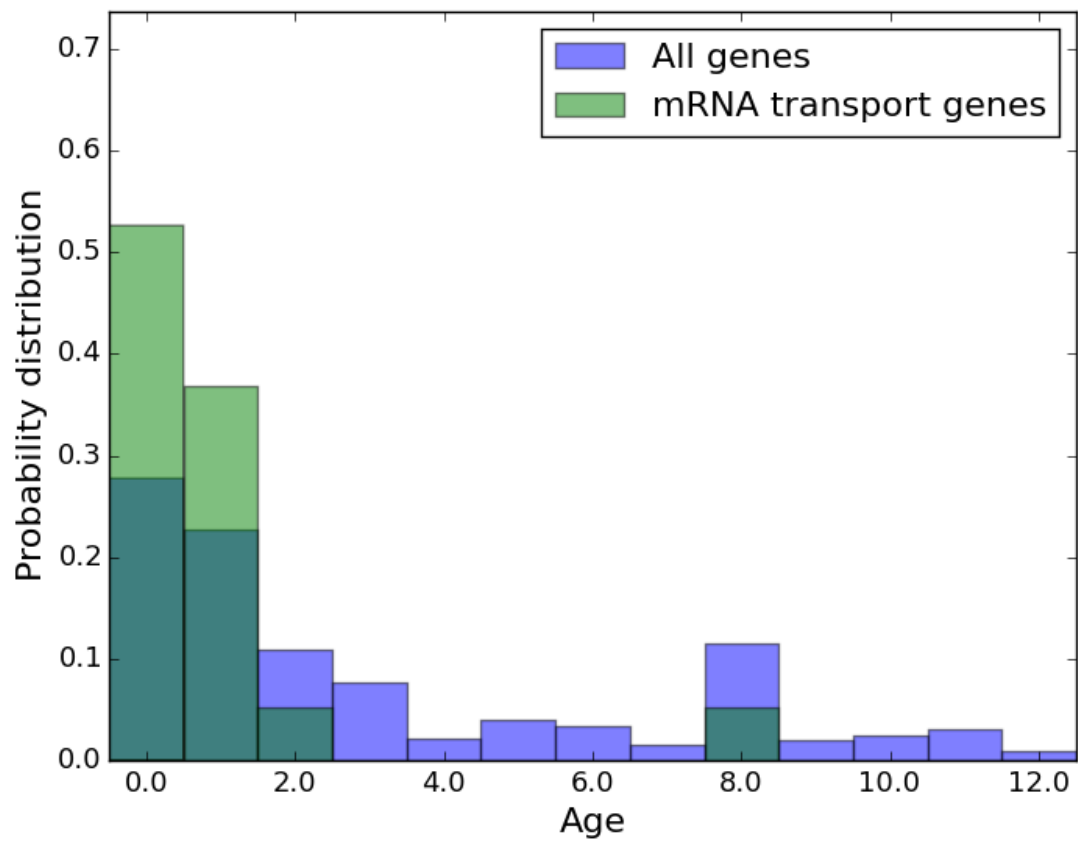

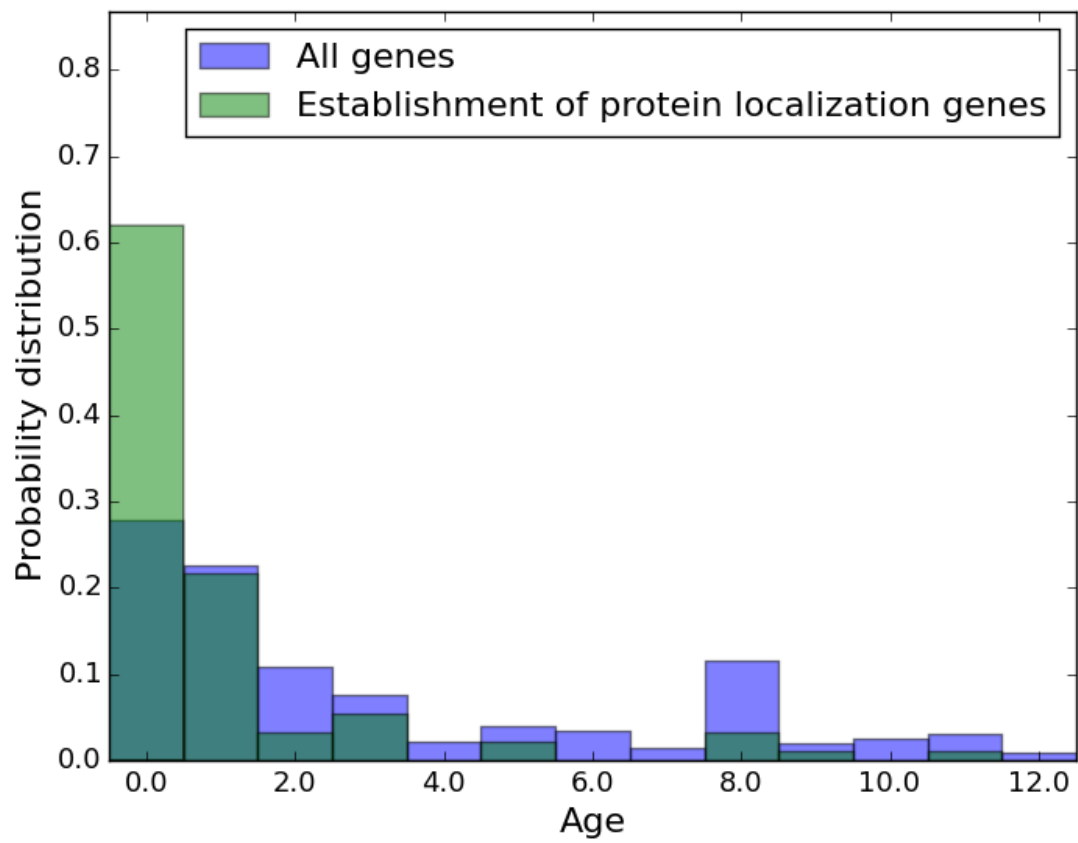

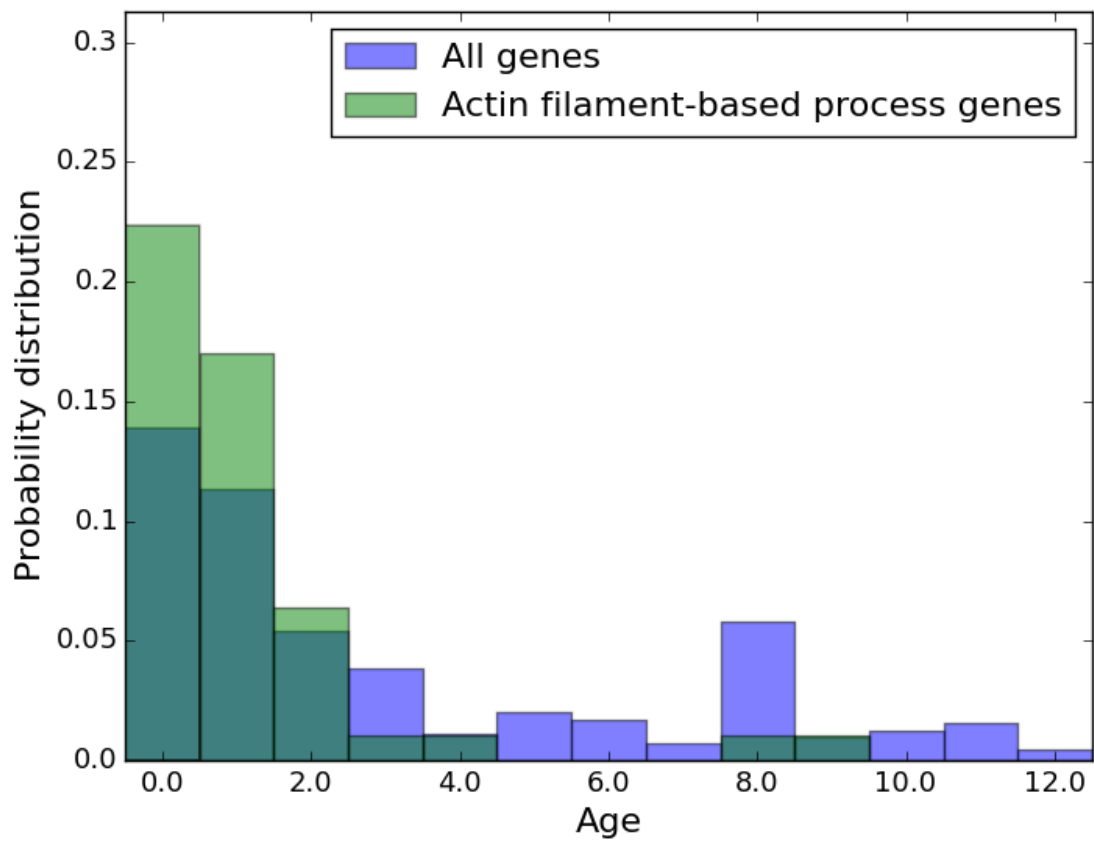

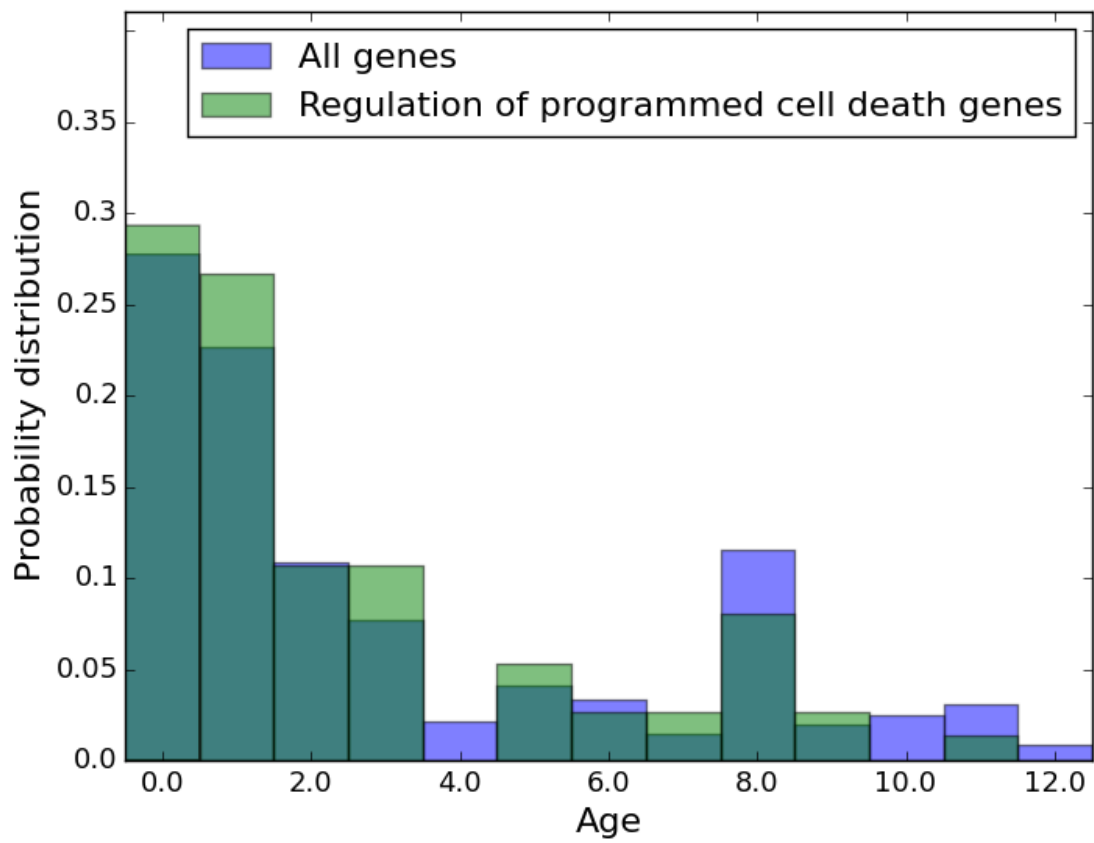

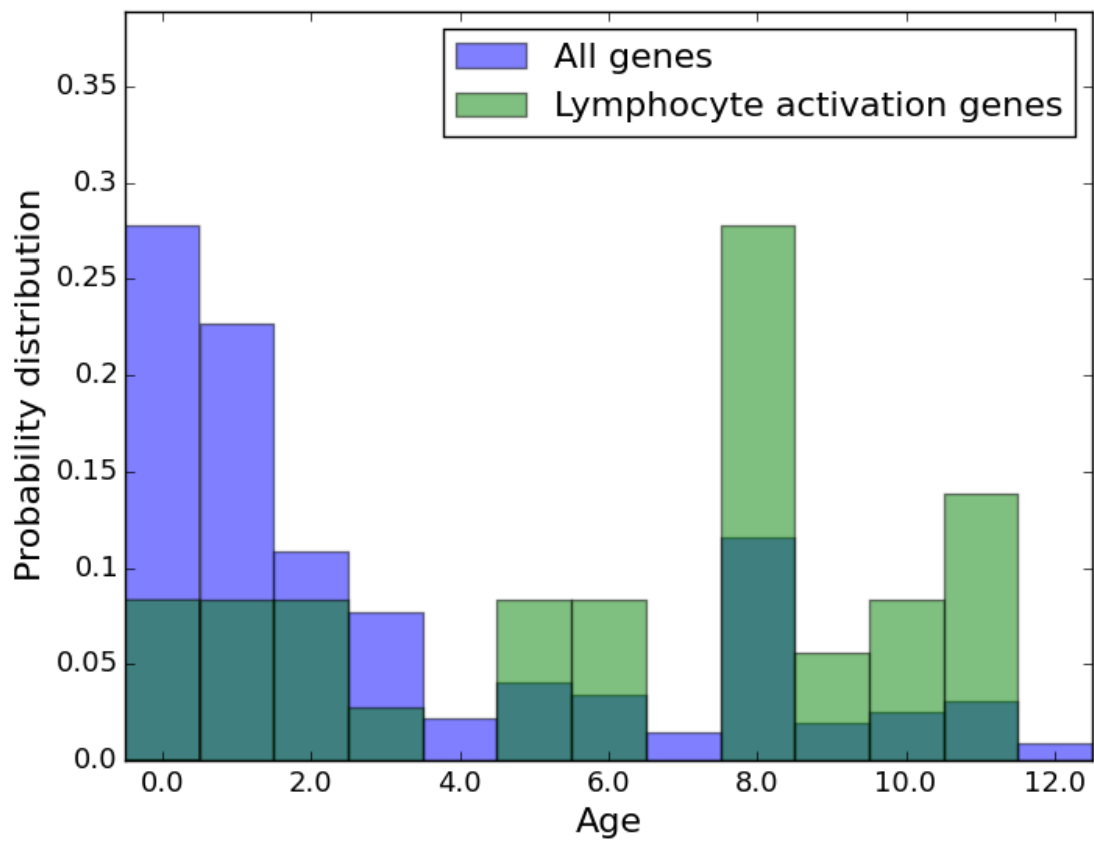

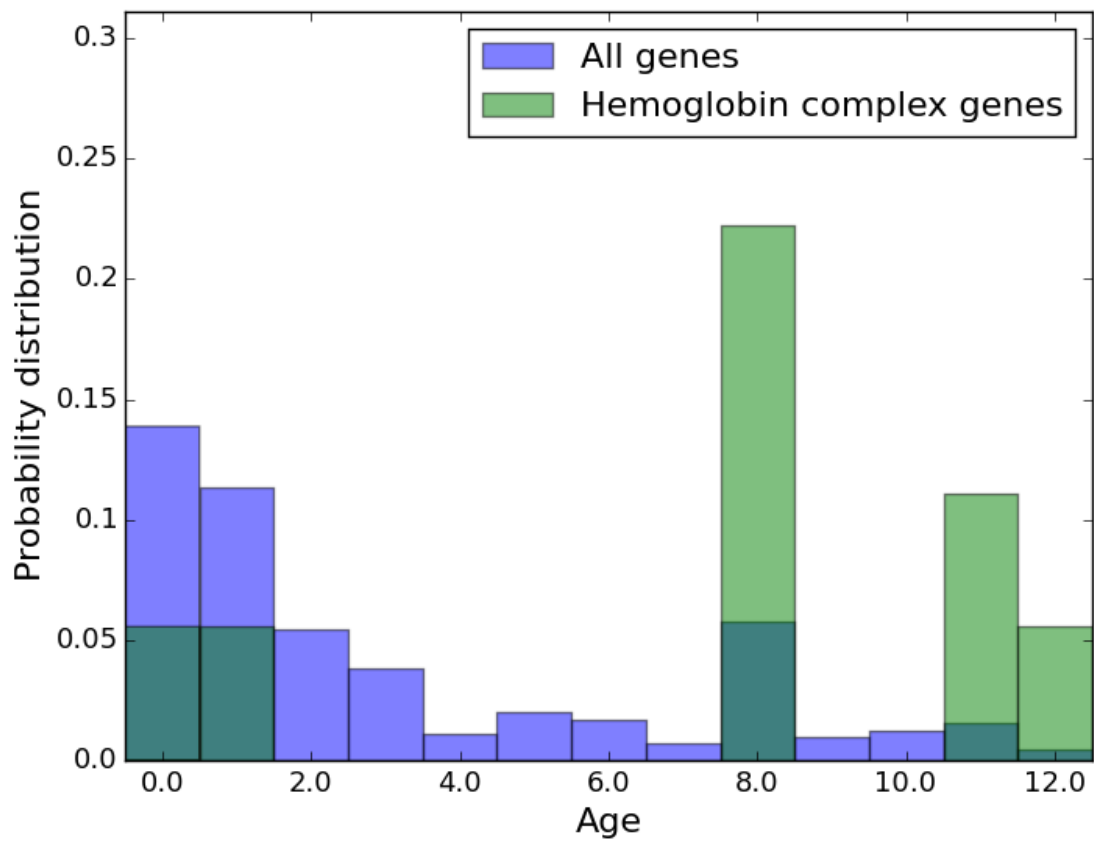

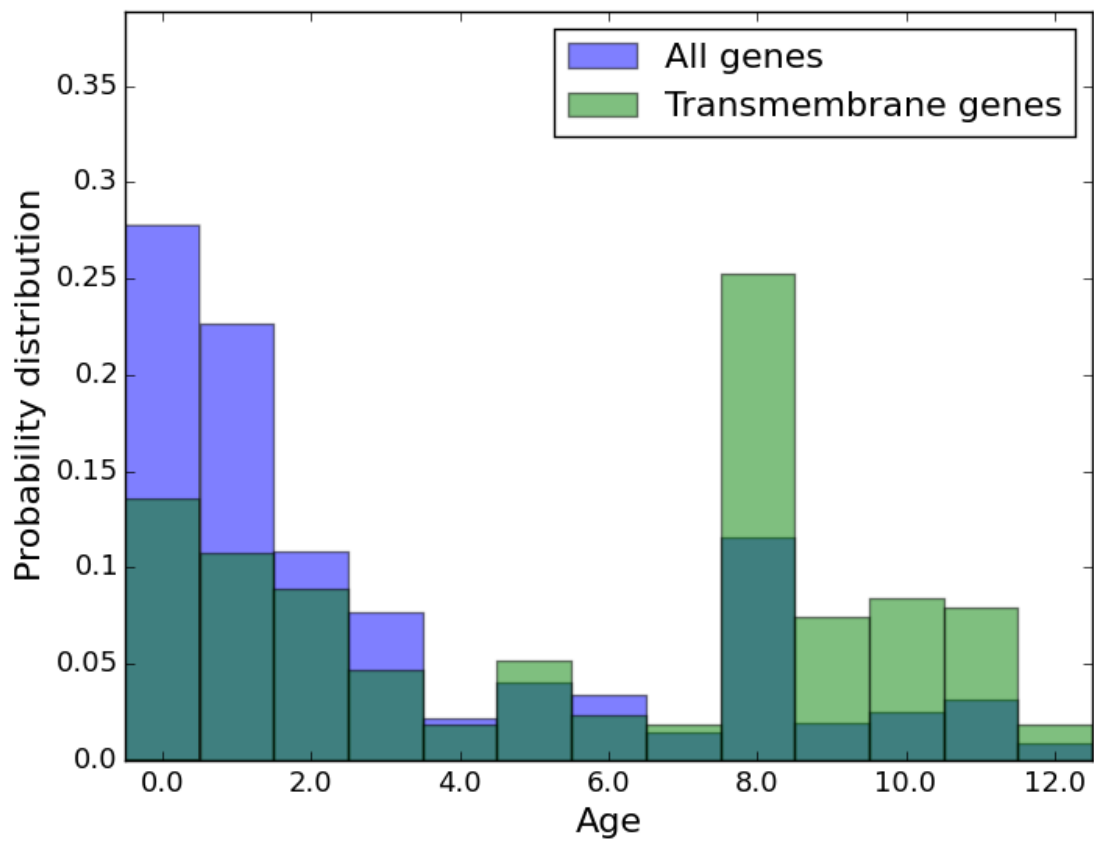

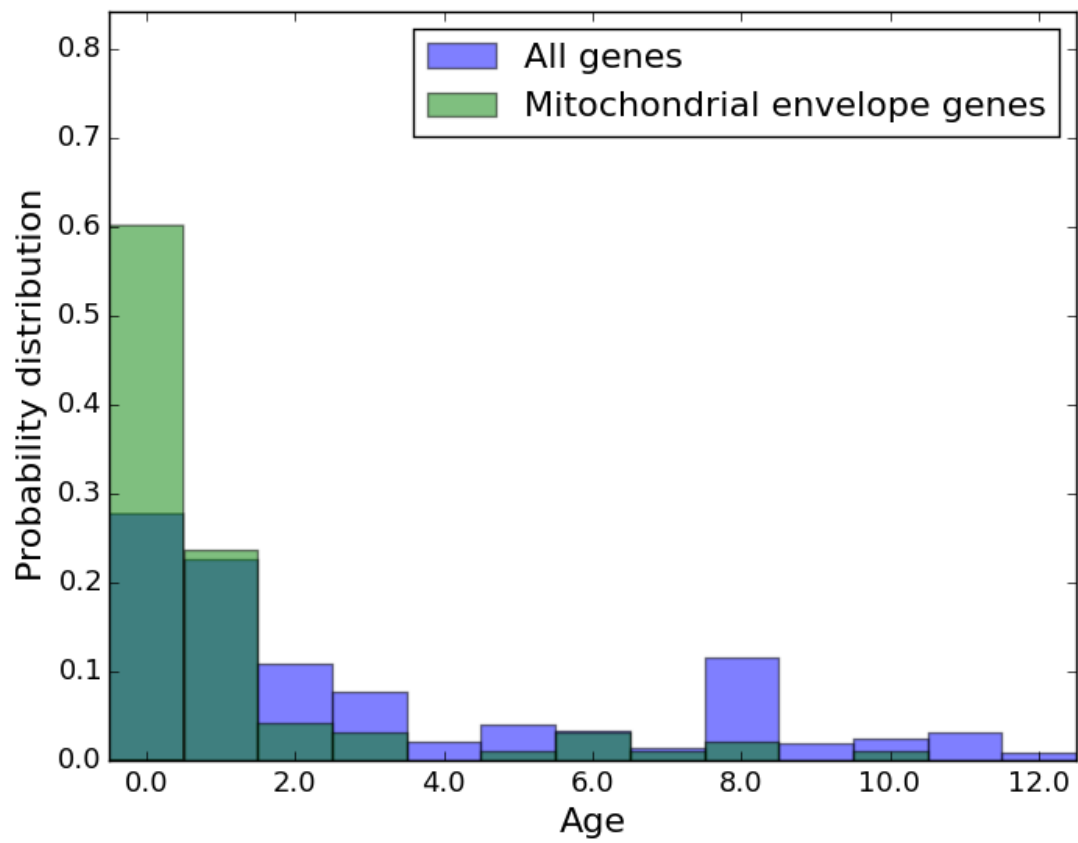

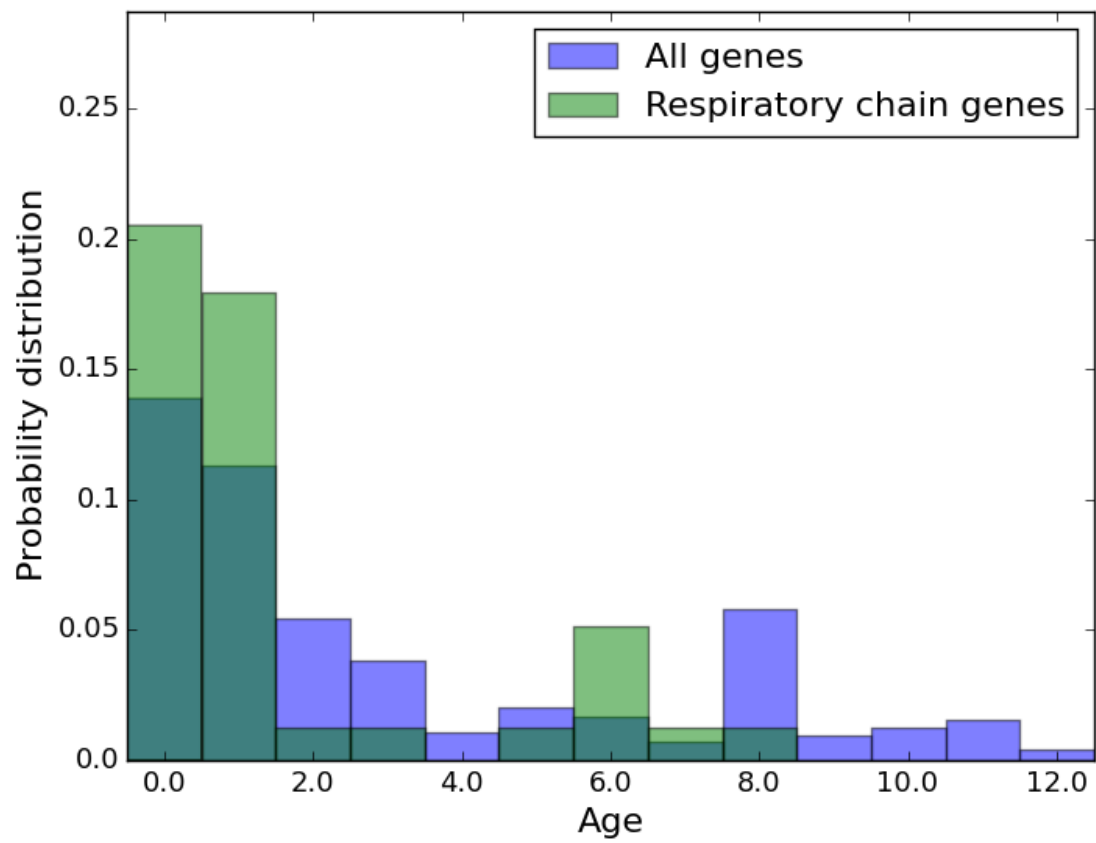

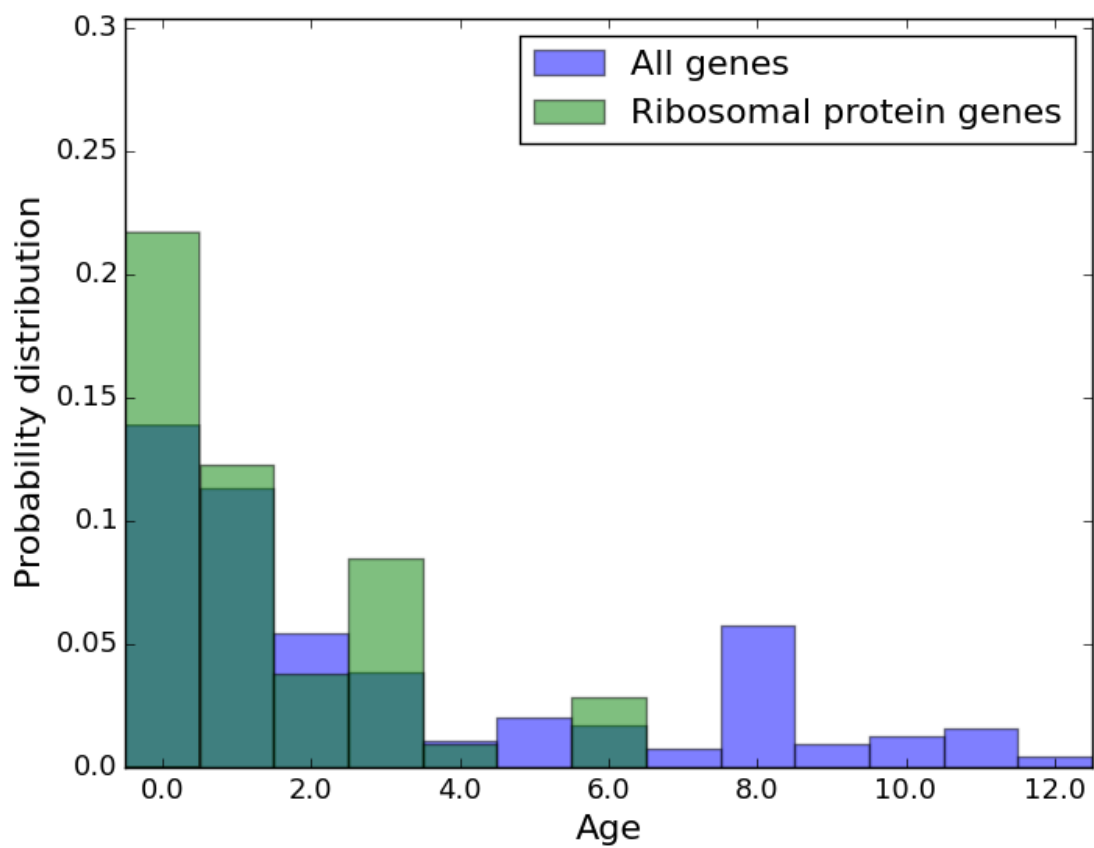

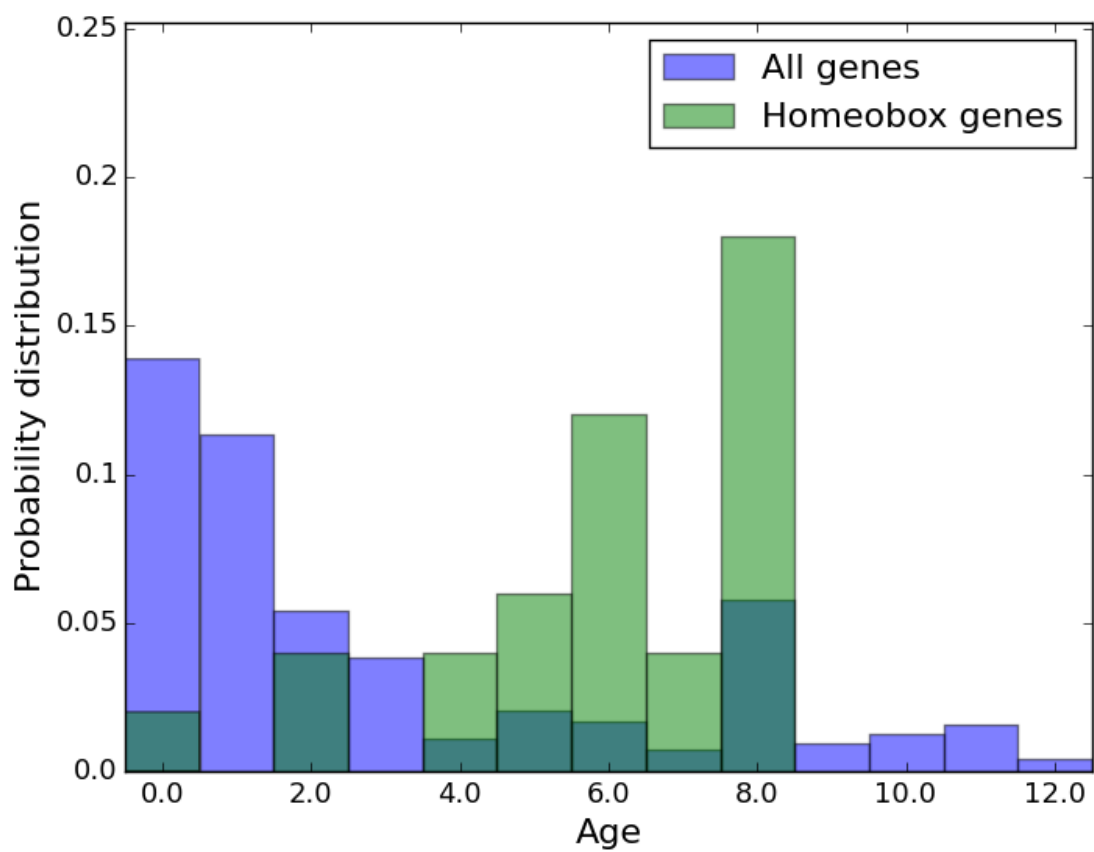

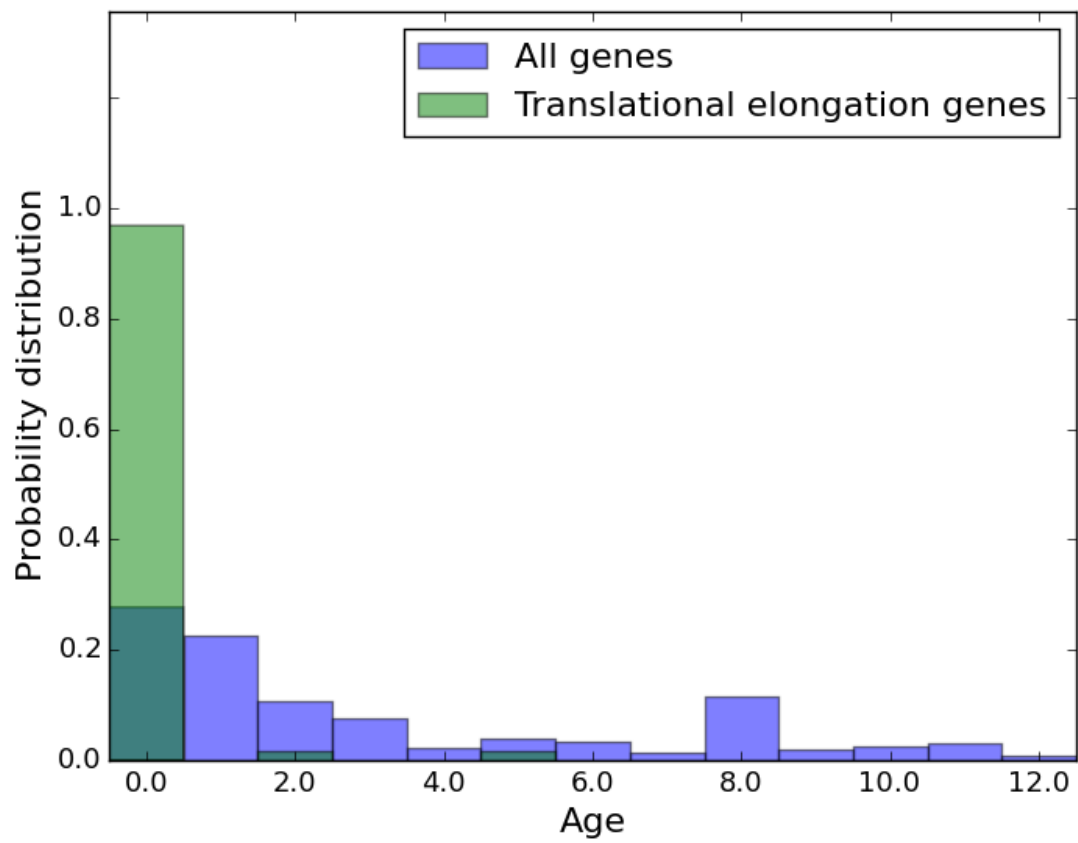

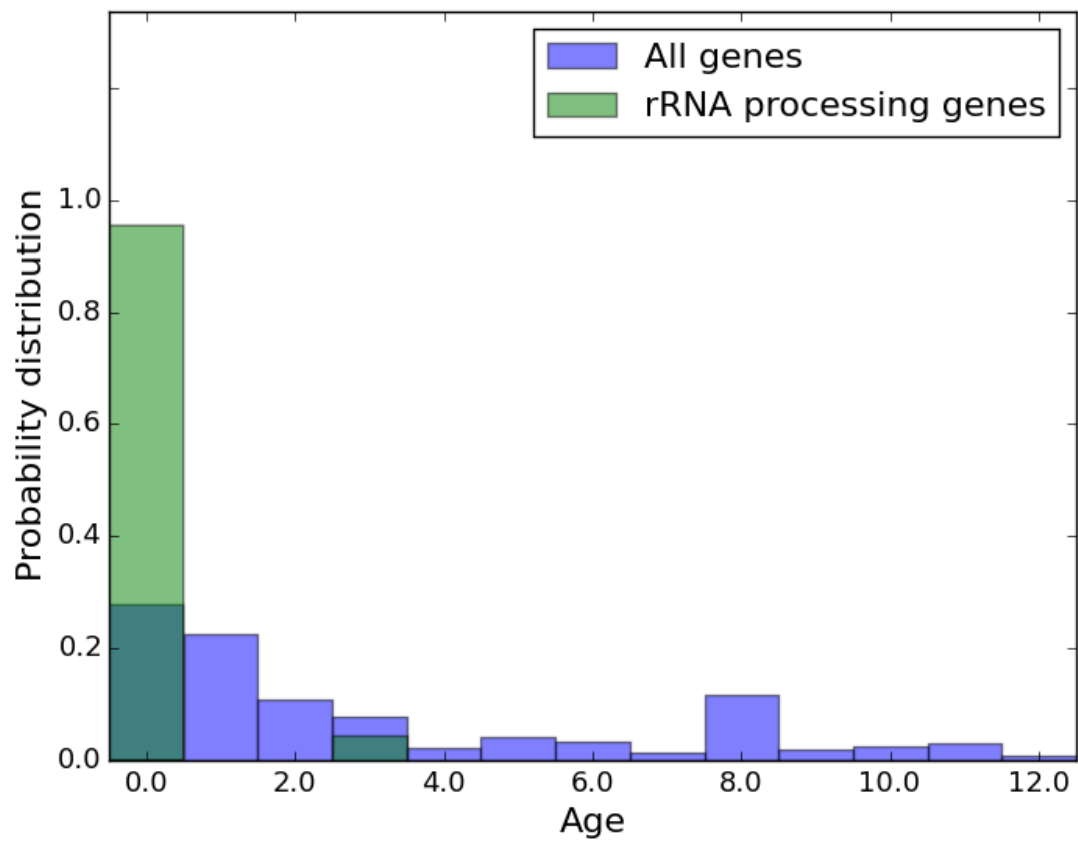

Supplement: S8 Fig — These figures show the distribution of ages for all communities and DAVID groups reported in Table 1. (PDF) [file pcbi.1005009.s008.pdf]
