# Supplementary material for: Evolutionary and Topological Properties of Genes and Community Structures in Human Gene Regulatory Networks
Source: PLoS Comput Biol. 2016 Jun 30;12(6):e1005009. doi: 10.1371/journal.pcbi.1005009 (PMC4928929; doi:10.1371/journal.pcbi.1005009)
Supplement: S4 File — (PDF) [file pcbi.1005009.s012.pdf]

We designed the interset efficiency so that it generalizes the set efficiency; in other words, the set efficiency is a special case of the interset efficiency:

$$E_{II} = E_I$$

(This result is derived on the next page.) $I = J$ is the "self term" or the "diagonal term" of the interset efficiency. It quantifies how efficiently nodes in set I signal to each other.

Definition of set efficiency (how efficiently nodes in set I signal to other nodes in set I):

$$E_I = \frac{1}{|I|(|I|-1)} \sum_{\substack{i \in I, j \in I \\ i \neq j}} \frac{1}{d_{ij}}$$

Definition of interset efficiency (how efficiently nodes in set J signal to nodes in set I):

$$E_{IJ} = \frac{1}{|I||J| - |I \cap J|} \sum_{\substack{i \in I, j \in J, \\ i \neq j}} \frac{1}{d_{ij}}$$

If $J = I$:

$$E_{II} = \frac{1}{|I||I| - |I \cap I|} \sum_{\substack{i \in I, j \in I, \\ i \neq j}} \frac{1}{d_{ij}}$$

$$= \frac{1}{|I|^2 - |I|} \sum_{\substack{i \in I, j \in I, \\ i \neq j}} \frac{1}{d_{ij}}$$

$$= \frac{1}{|I|(|I|-1)} \sum_{\substack{i \in I, j \in I, \\ i \neq j}} \frac{1}{d_{ij}} \equiv E_I$$

Without overlap between sets

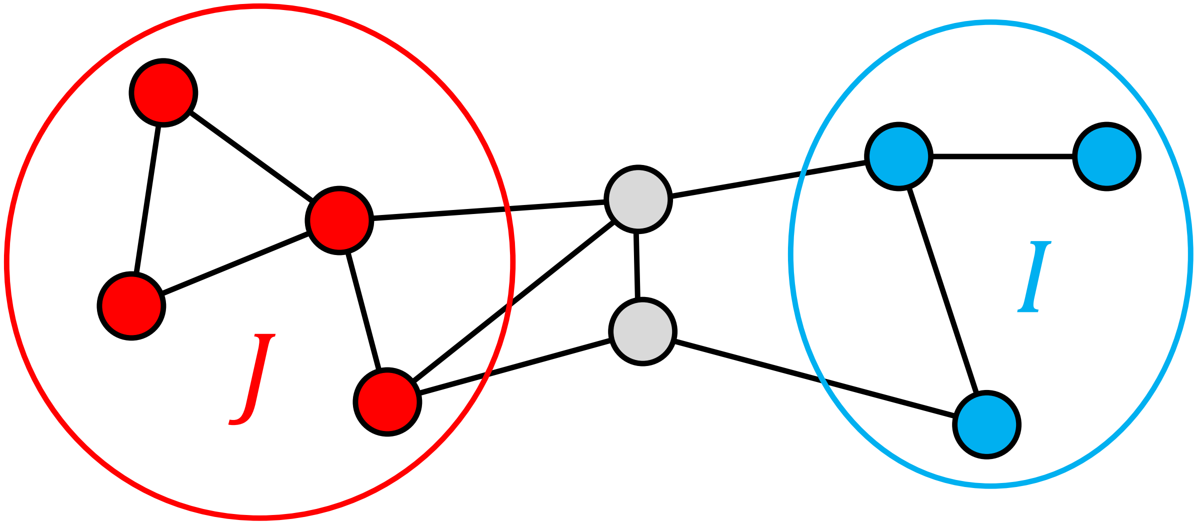

$$E_{IJ} = \frac{1}{|I||J| - |I \cap J|} \sum_{\substack{i \in I, j \in J, \\ i \neq j}} \frac{1}{d_{ij}}$$

Mean inverse distance from each node in J to each node in I. There are $|I||J| - |I \cap J| = 4 \times 3 - 0 = 12$ terms in the summation.

Without overlap between sets

Some of the 12 terms are represented by paths with thick edges below

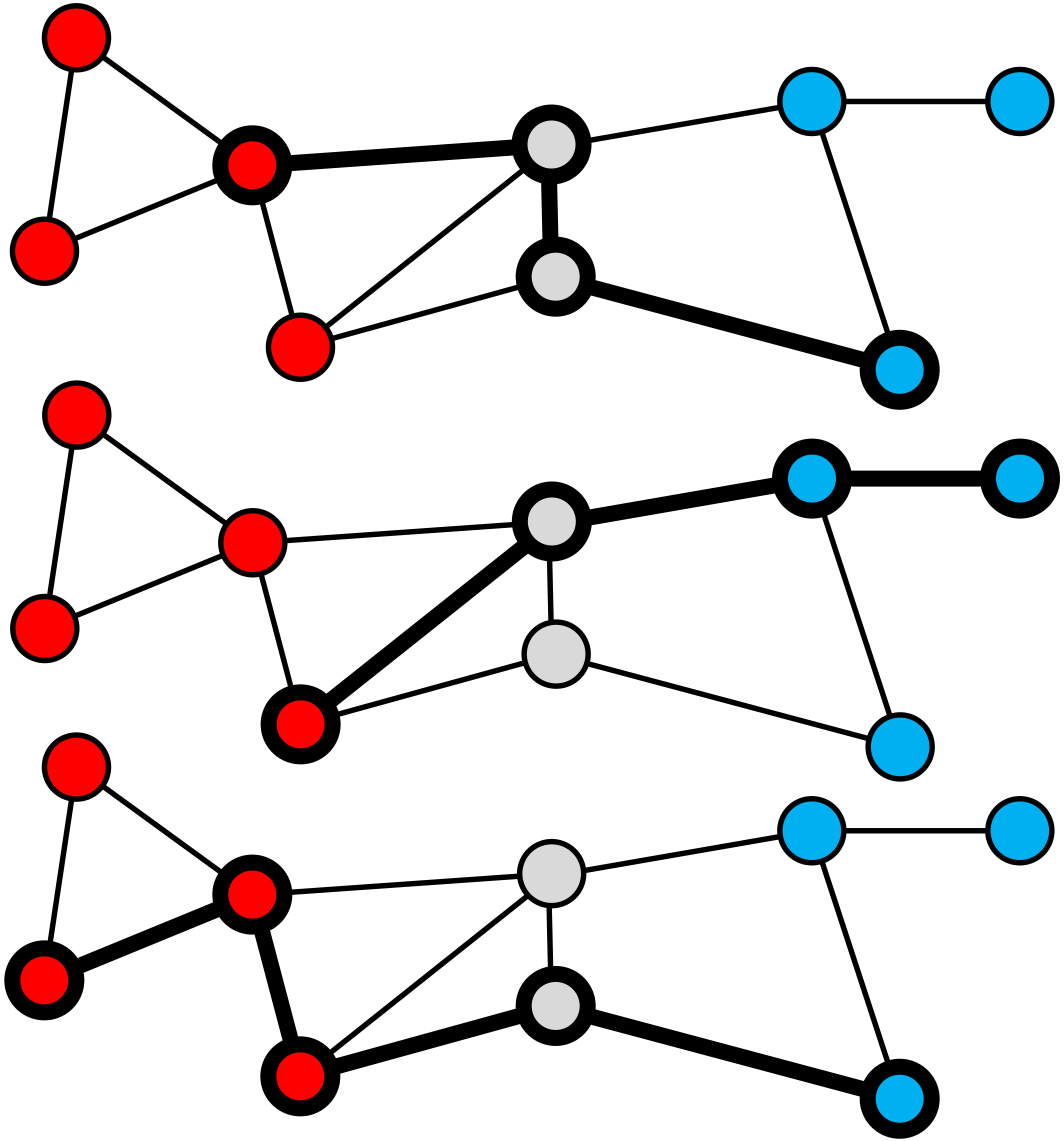

With overlap between sets

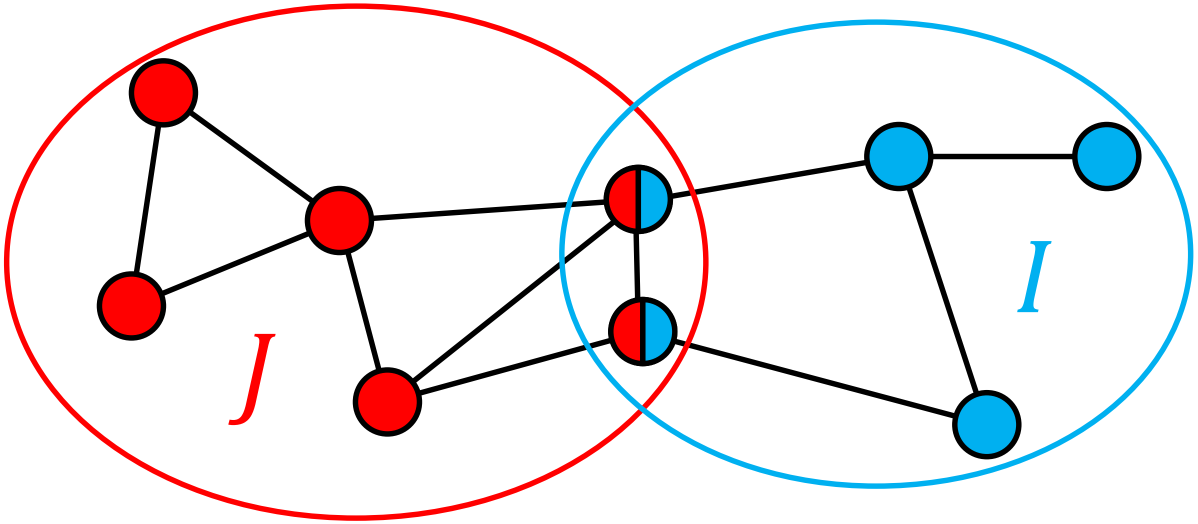

$$E_{IJ} = \frac{1}{|I||J| - |I \cap J|} \sum_{\substack{i \in I, j \in J, \\ i \neq j}} \frac{1}{d_{ij}}$$

Mean inverse distance from each node in J to each node in I. There are $|I||J| - |I \cap J| = 6 \times 5 - 2 = 28$ terms in the summation.

Without overlap between sets

Some of the 28 terms are represented by paths with thick edges below

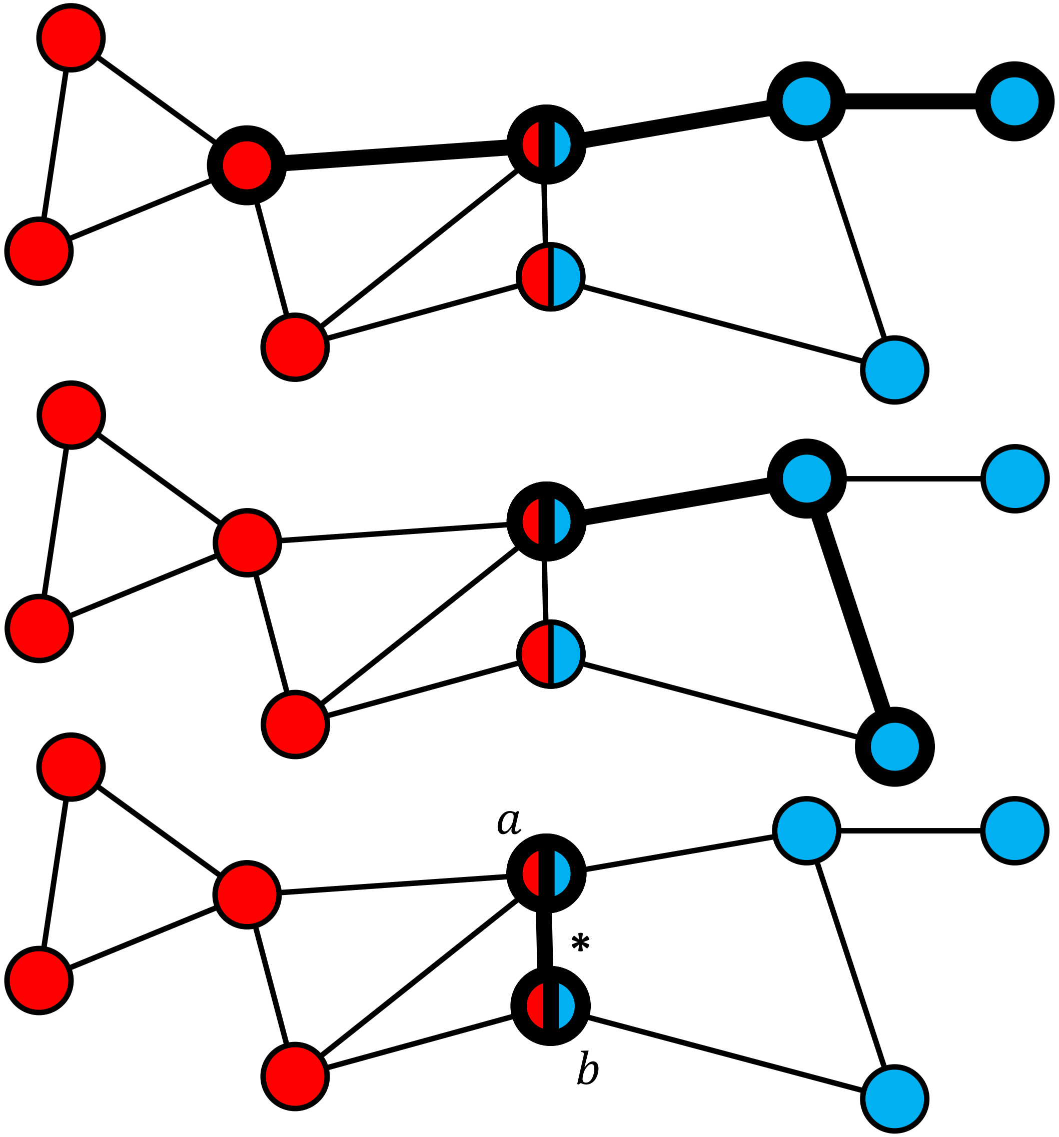

*Note: As with all pairs of nodes in the intersection of the sets I and J, both the $a \rightarrow b$ term and the $b \rightarrow a$ term appear in the interset efficiency equation. A larger overlap between sets I and J implies a stronger relationship between the function of the sets, and we designed the interset efficiency to reflect this.

With $I = J$

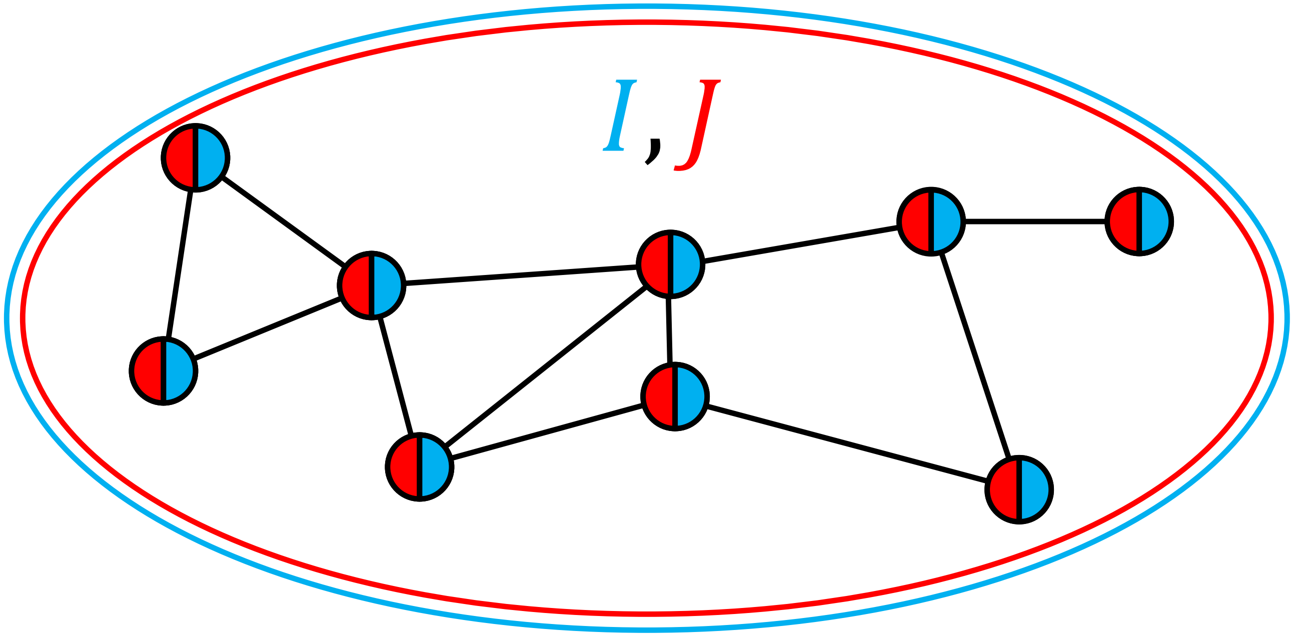

$$E_{IJ} = \frac{1}{|I||J| - |I \cap J|} \sum_{\substack{i \in I, j \in J, \\ i \neq j}} \frac{1}{d_{ij}}$$

Mean inverse distance from each node in J to each node in I. Since $I = J$, there are $|I||J| - |I \cap J| = |I|(|I| - 1) = 72$ terms in the summation.

Without overlap between sets

Some of the 72 terms are represented by paths with thick edges below

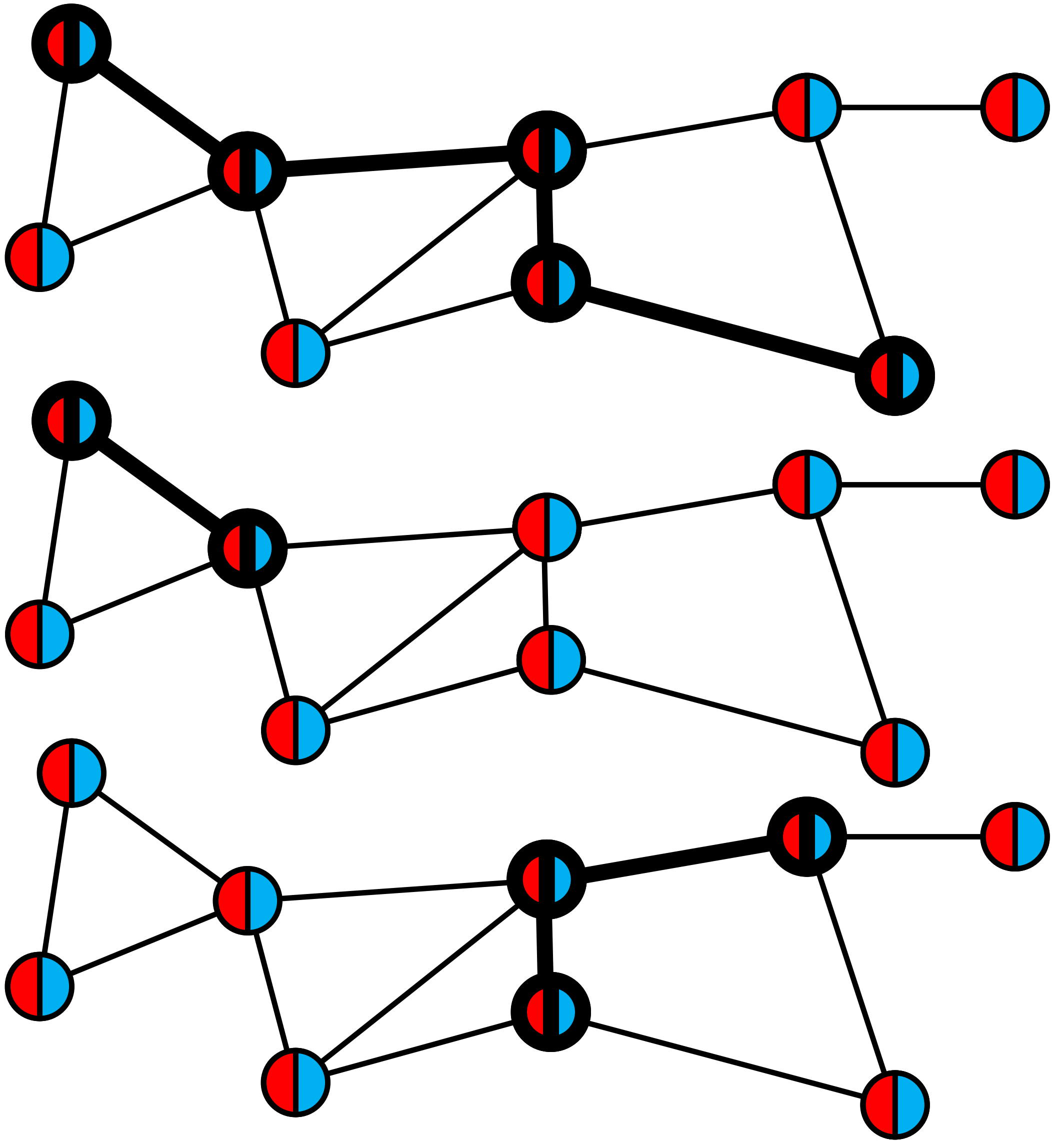

A low set efficiency implies dispersed nodes…

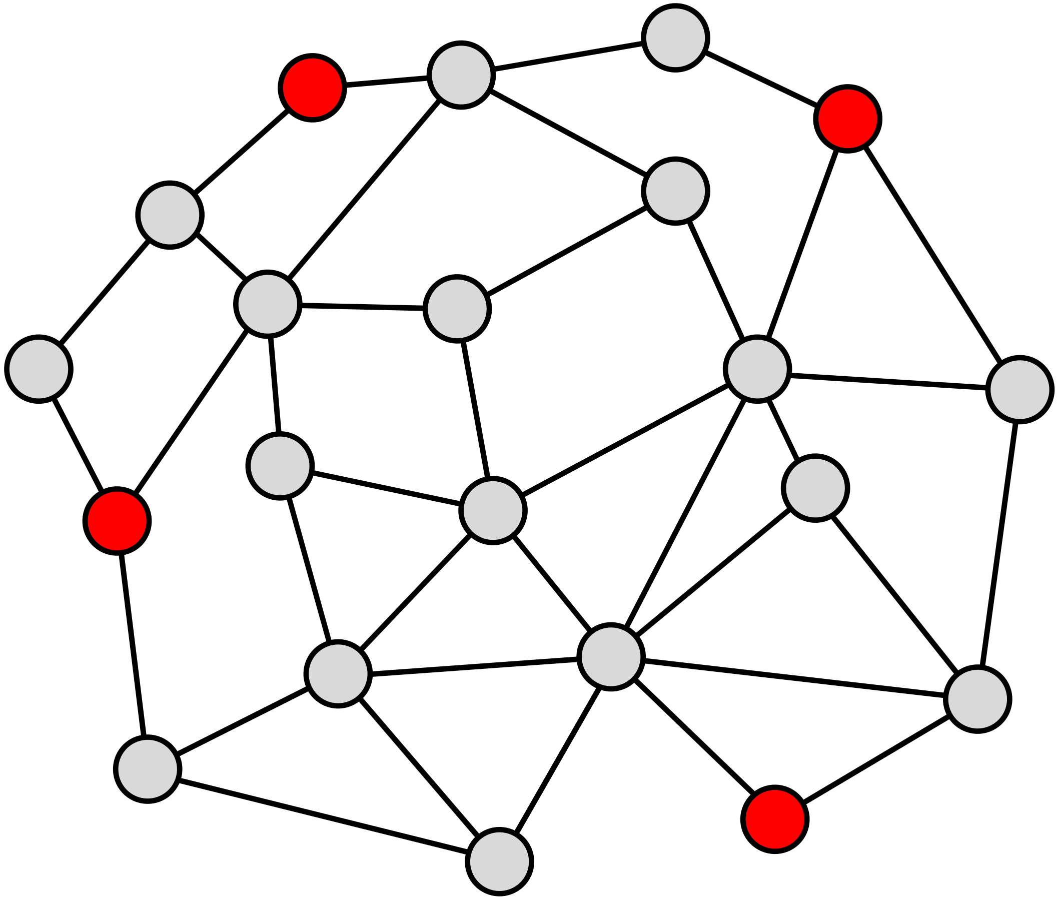

… and a high set efficiency implies concentrated nodes

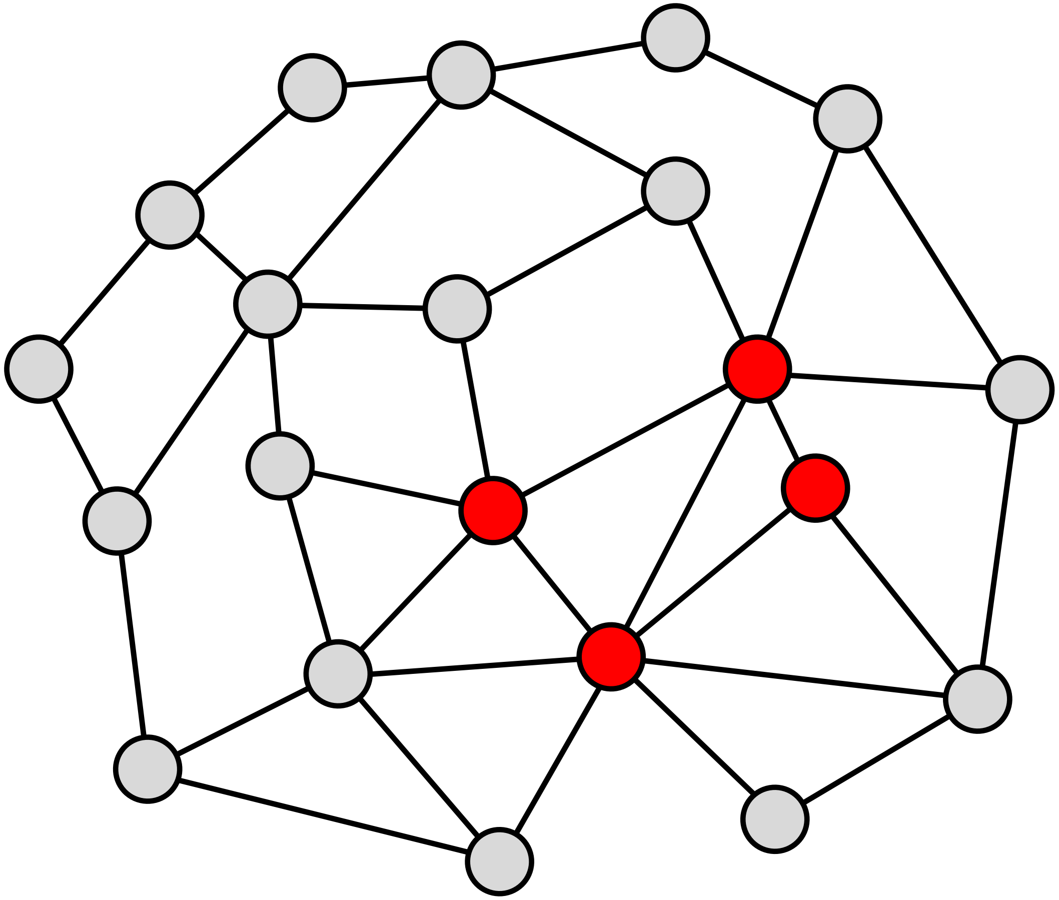

A low interset efficiency implies well-separated sets…

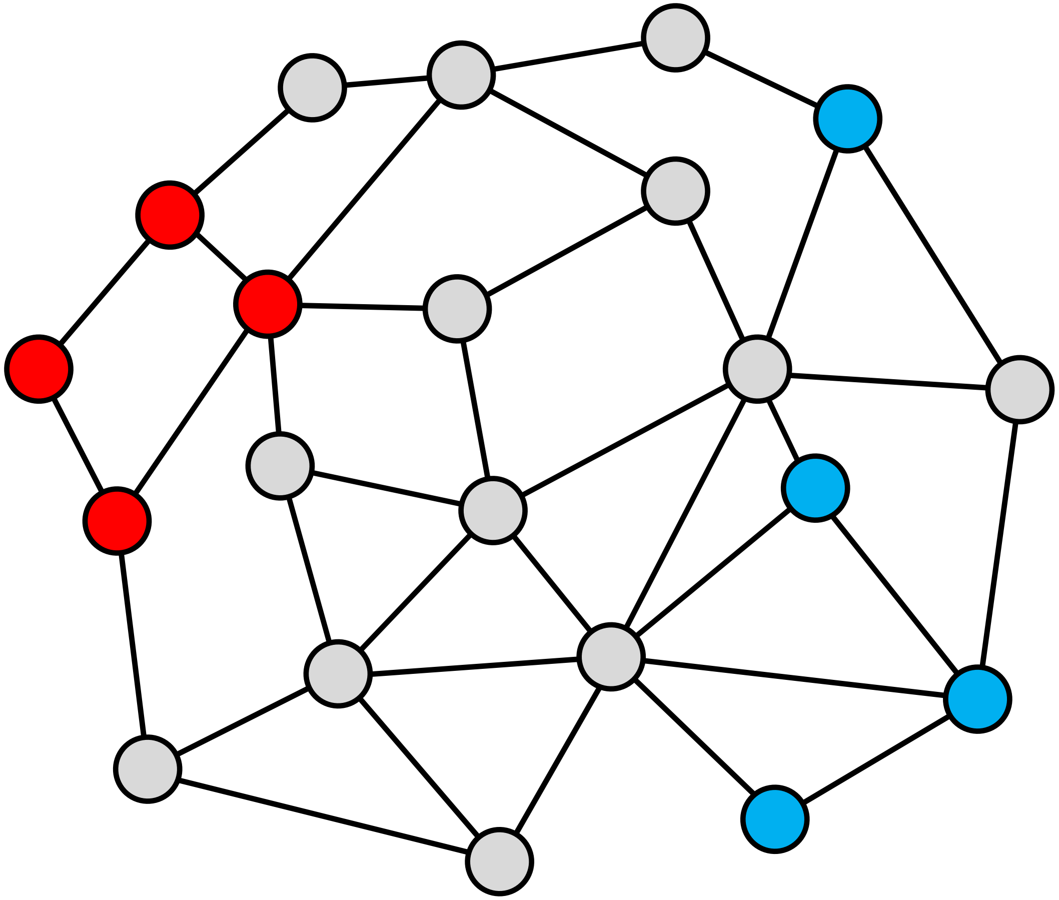

… and a high interset efficiency implies close (and maybe overlapping) sets

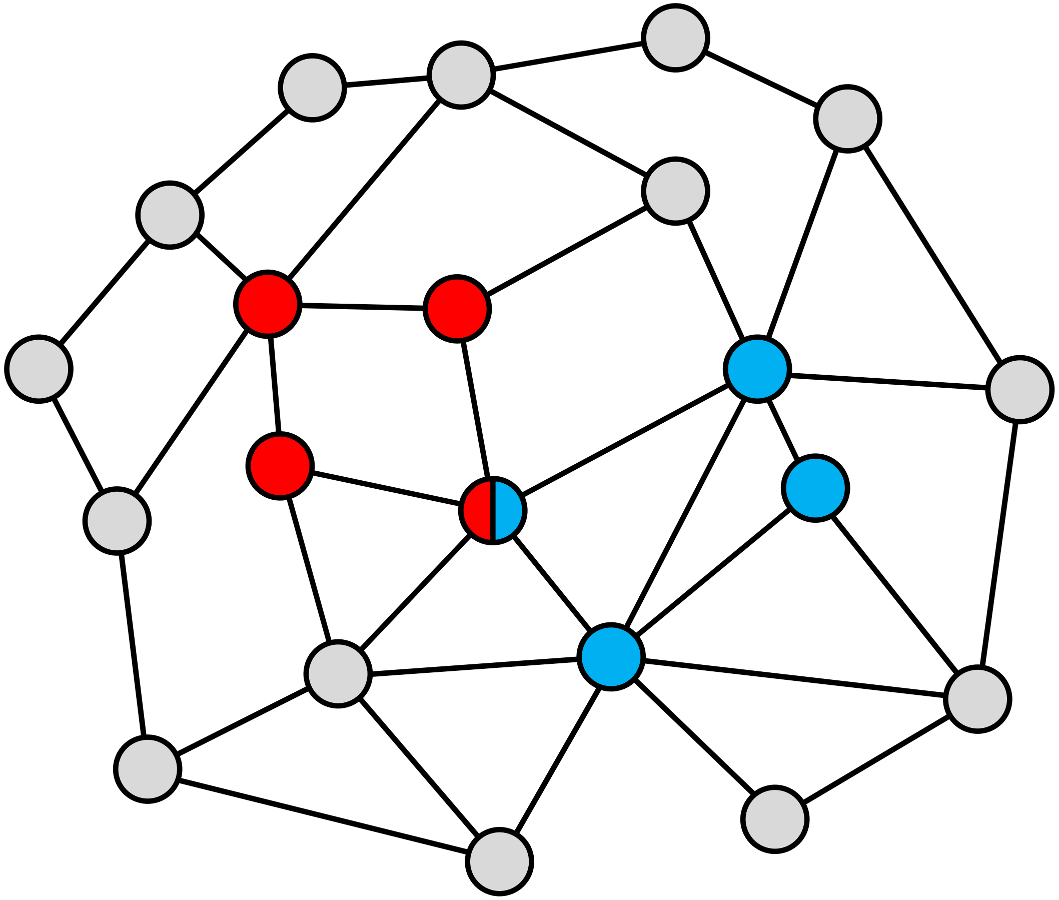

Derivation of normalization

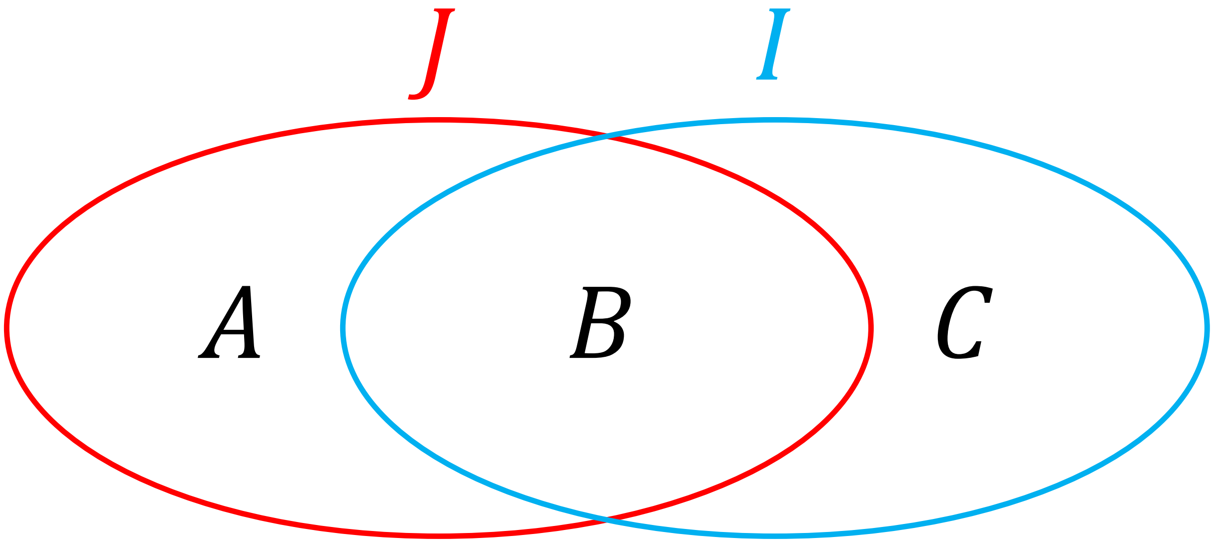

We want to include a term in the summation for each relevant pair of nodes. The pairs are chosen according to the following rule: the source node must belong to set J, and the target node must belong to set I. This means that paths can begin and/or end in the intersection of these sets. For convenience, define the following:

$$A = J \setminus I \qquad B = I \cap J \qquad C = I \setminus J$$

Derivation of normalization

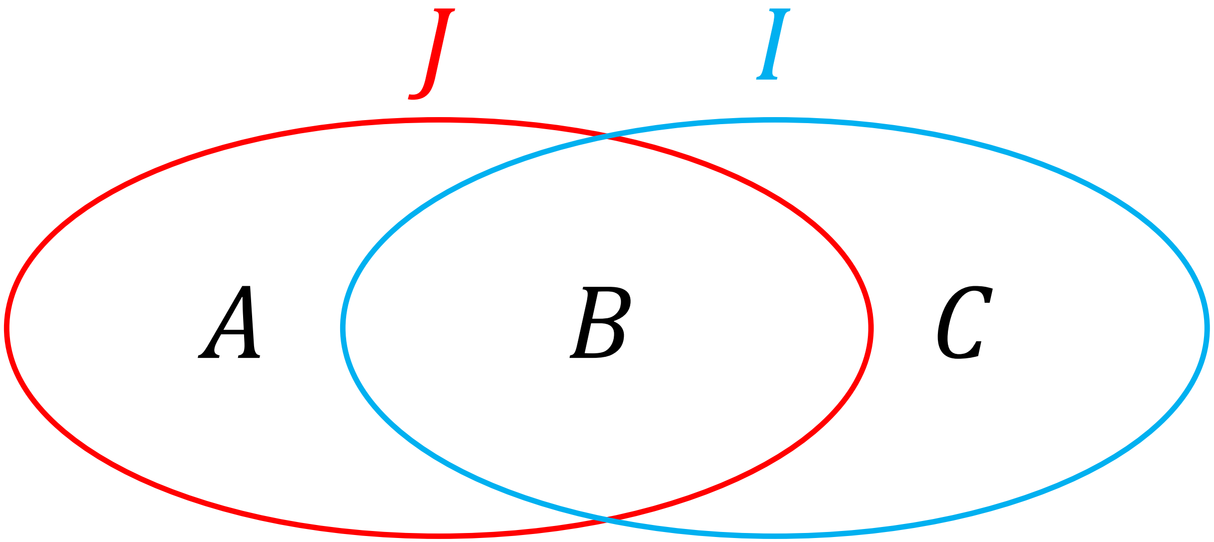

Decomposing sets I and J into nonoverlapping sets A, B, and C makes it easier to count the relevant pairs. We now simply need to count the number of pairs from (1) A to B; (2) B to C; (3) A to C; and (4) B to B.

$$A = J \setminus I \qquad B = I \cap J \qquad C = I \setminus J$$

Derivation of normalization

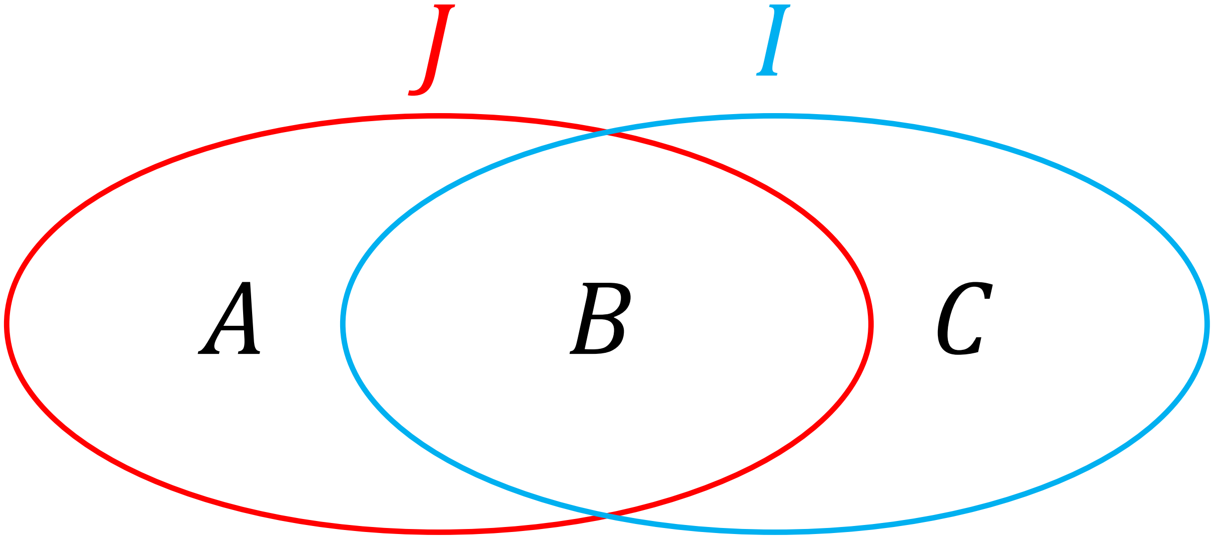

Number of pairs where one node is chosen from A and the other is chosen from B:

$$|A| \times |B| = |J \setminus I| \times |I \cap J|$$

Derivation of normalization

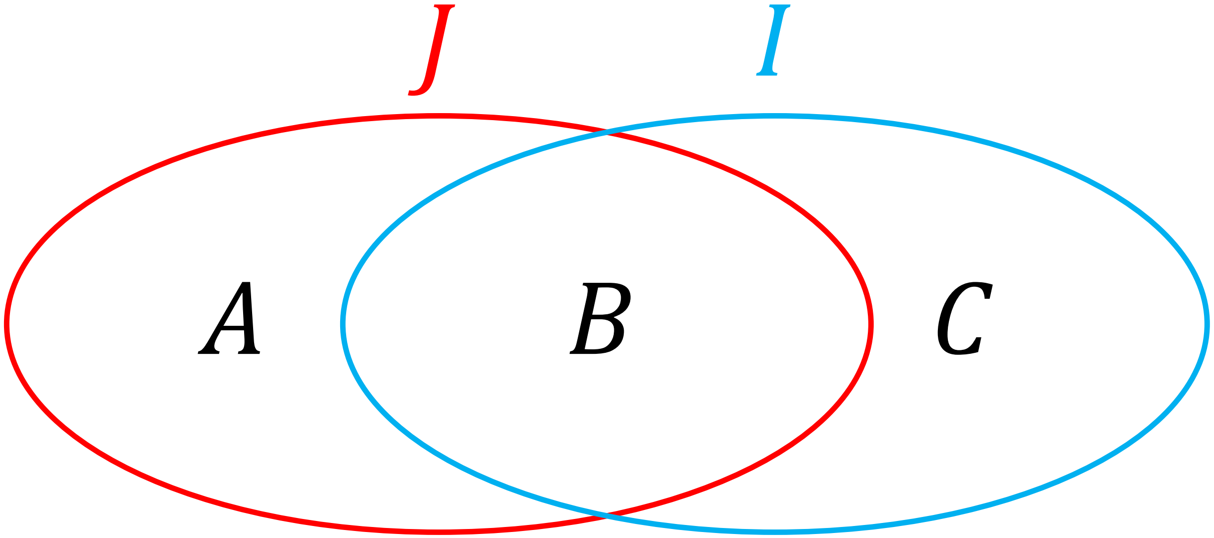

Number of pairs where one node is chosen from B and the other is chosen from C:

$$|B| \times |C| = |I \cap J| \times |I \setminus J|$$

Derivation of normalization

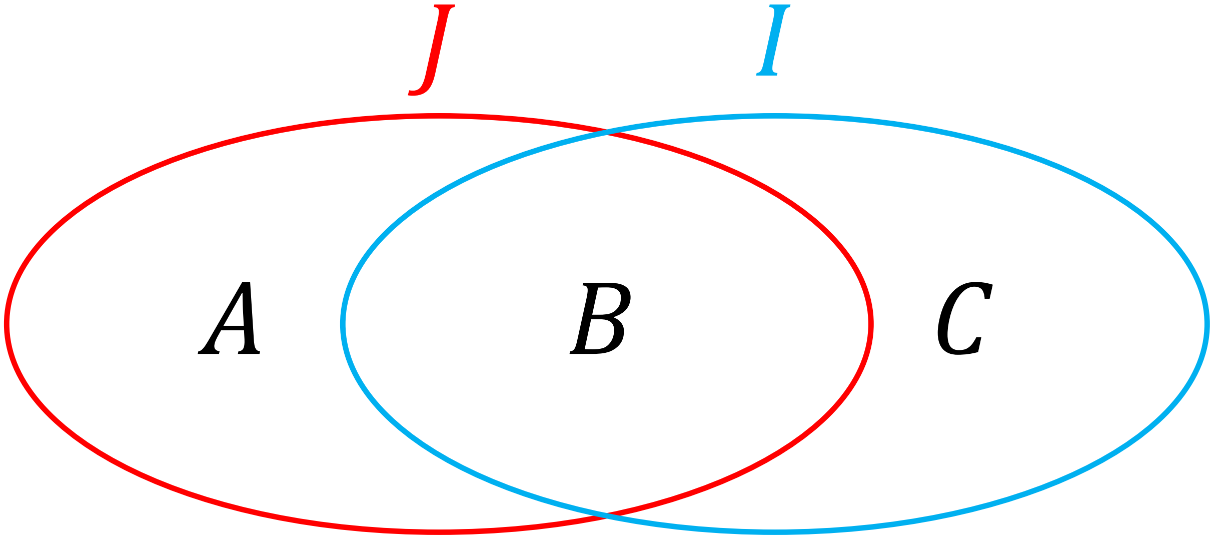

Number of pairs where one node is chosen from A and the other is chosen from C:

$$|A| \times |C| = |J \setminus I| \times |I \setminus J|$$

Derivation of normalization

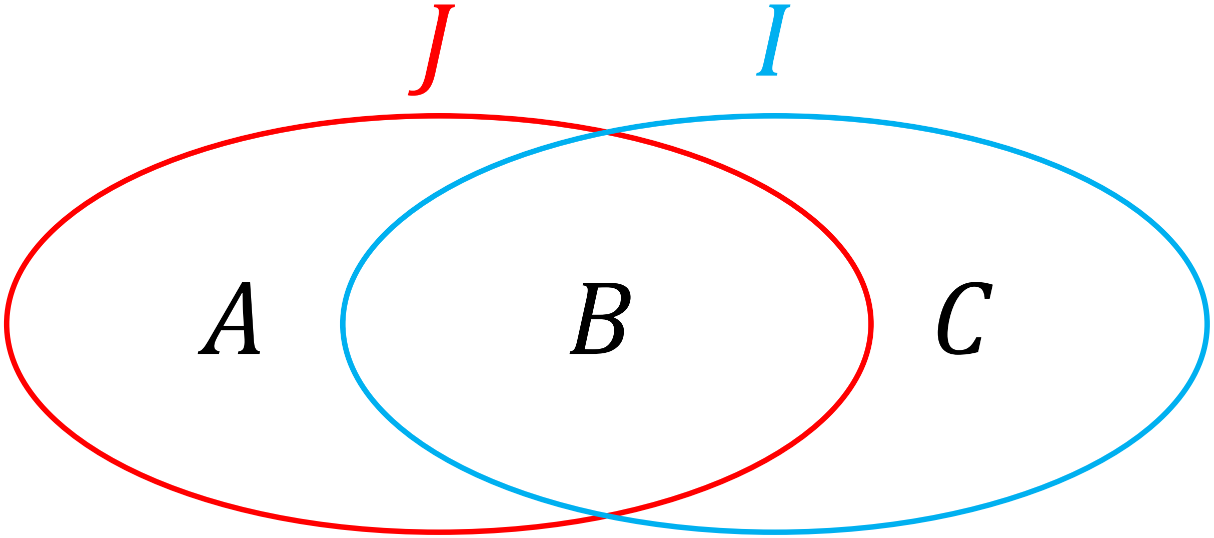

Number of pairs where **both** nodes are chosen from B (and the chosen nodes are different):

$$|B| \times (|B| - 1)$$

$$= |I \cap J| \times (|I \cap J| - 1)$$

= All nodes in the intersection to all *other* nodes in the intersection

Derivation of normalization

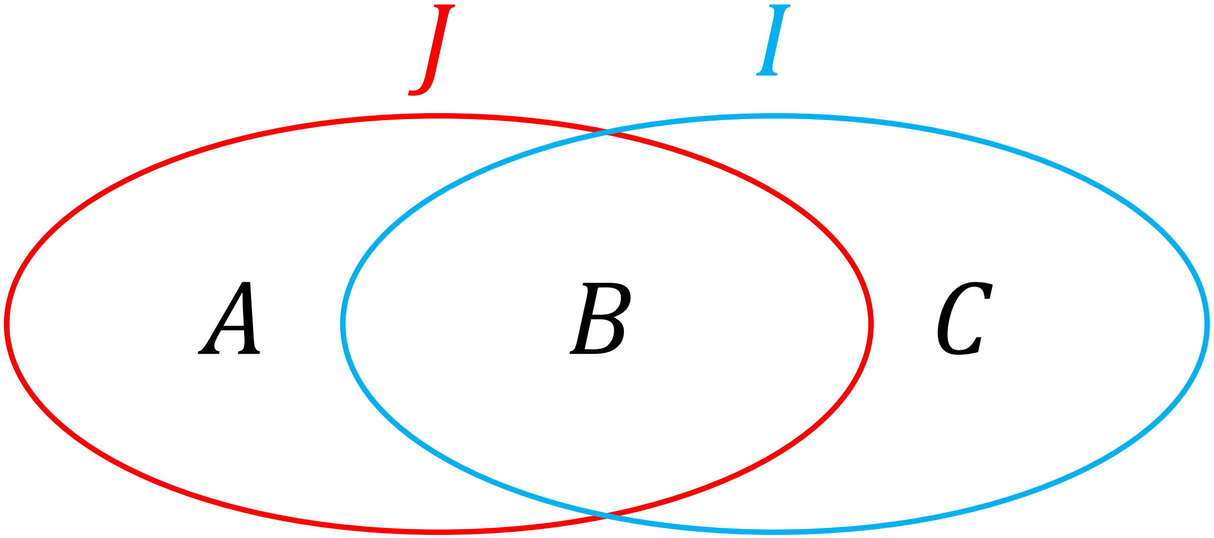

Put it all together:

$$
\begin{aligned}
\text{Normalization} = {} & |J \setminus I| \times |I \cap J| + && (A \text{ to } B) \\
& |I \cap J| \times |I \setminus J| + && (B \text{ to } C) \\
& |J \setminus I| \times |I \setminus J| + && (A \text{ to } C) \\
& |I \cap J| \times (|I \cap J| - 1) && (B \text{ to } B)
\end{aligned}
$$

Derivation of normalization

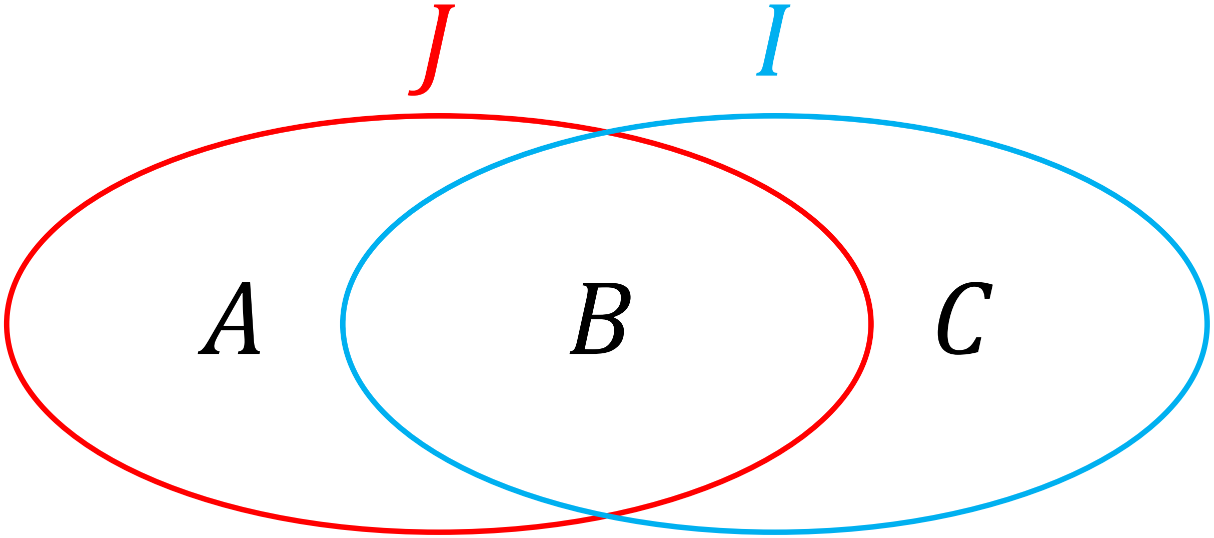

... but this can be simplified. Note that:

$$|I \setminus J| = |I| - |I \cap J|$$
$$|J \setminus I| = |J| - |I \cap J|$$

Derivation of normalization

Substitute and simplify:

$$\text{Normalization} = |J \setminus I| \times |I \cap J| + |I \cap J| \times |I \setminus J| + |J \setminus I| \times |I \setminus J| + |I \cap J| \times (|I \cap J| - 1)$$

$$= |J||I \cap J| + |I||I \cap J| - 2|I \cap J|^2 + |I||J| - |J||I \cap J| - |I||I \cap J| + |I \cap J|^2 + |I \cap J|^2 - |I \cap J|$$

(color-coded cancelation)

$$= |I||J| - |I \cap J|$$ ✓
